# Recognition of single-stranded nucleic acids by small-molecule splicing modulators

**DOI:** 10.1093/nar/gkab602

**Published:** 2021-07-20

**Authors:** Zhichao Tang, Sana Akhter, Ankita Ramprasad, Xiao Wang, Mikhail Reibarkh, Jinan Wang, Sadikshya Aryal, Srinivas S Thota, Junxing Zhao, Justin T Douglas, Philip Gao, Erik D Holmstrom, Yinglong Miao, Jingxin Wang

**Affiliations:** Department of Medicinal Chemistry, University of Kansas, Lawrence, KS 66047, USA; Center for Computational Biology and Department of Molecular Biosciences, University of Kansas, Lawrence, KS 66047, USA; Department of Medicinal Chemistry, University of Kansas, Lawrence, KS 66047, USA; Analytical Research & Development, Merck and Co., Inc., Kenilworth, NJ 07033, USA; Analytical Research & Development, Merck and Co., Inc., Kenilworth, NJ 07033, USA; Center for Computational Biology and Department of Molecular Biosciences, University of Kansas, Lawrence, KS 66047, USA; Department of Medicinal Chemistry, University of Kansas, Lawrence, KS 66047, USA; Department of Medicinal Chemistry, University of Kansas, Lawrence, KS 66047, USA; Department of Medicinal Chemistry, University of Kansas, Lawrence, KS 66047, USA; Nuclear Magnetic Resonance Lab, University of Kansas, Lawrence, KS 66045, USA; Protein Production Group, University of Kansas, Lawrence, KS 66047, USA; Department of Molecular Biosciences and Department of Chemistry, University of Kansas, Lawrence, KS 66045, USA; Center for Computational Biology and Department of Molecular Biosciences, University of Kansas, Lawrence, KS 66047, USA; Department of Medicinal Chemistry, University of Kansas, Lawrence, KS 66047, USA

## Abstract

Risdiplam is the first approved small-molecule splicing modulator for the treatment of spinal muscular atrophy (SMA). Previous studies demonstrated that risdiplam analogues have two separate binding sites in exon 7 of the SMN2 pre-mRNA: (i) the 5′-splice site and (ii) an upstream purine (GA)-rich binding site. Importantly, the sequence of this GA-rich binding site significantly enhanced the potency of risdiplam analogues. In this report, we unambiguously determined that a known risdiplam analogue, SMN-C2, binds to single-stranded GA-rich RNA in a sequence-specific manner. The minimum required binding sequence for SMN-C2 was identified as GAAGGAAGG. We performed all-atom simulations using a robust Gaussian accelerated molecular dynamics (GaMD) method, which captured spontaneous binding of a risdiplam analogue to the target nucleic acids. We uncovered, for the first time, a ligand-binding pocket formed by two sequential GAAG loop-like structures. The simulation findings were highly consistent with experimental data obtained from saturation transfer difference (STD) NMR and structure-affinity-relationship studies of the risdiplam analogues. Together, these studies illuminate us to understand the molecular basis of single-stranded purine-rich RNA recognition by small-molecule splicing modulators with an unprecedented binding mode.

## Graphical Abstract

**Graphical Abstract:**
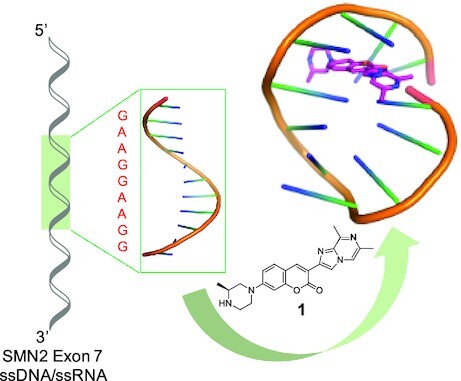
Risdiplam analogues bind to GA-rich DNAs and RNAs in double loop-like conformations.

## INTRODUCTION

Spinal muscular atrophy (SMA) is one of the most common lethal genetic diseases in new-borns (1,2). In the most severe type of SMA (type I), infants usually cannot survive beyond their first two years of life due to progressive hypotonia and respiratory failure (3). The cause of SMA in most type I patients is a recessive homozygous deletion within the survival of motor neuron (SMN) 1 gene in chromosome 5 (1,2). There are two nearly identical SMN genes in humans: SMN1 and SMN2. However, the protein produced by SMN2 cannot fully compensate for the loss of SMN1 in type I SMA patients. Earlier studies demonstrated that a single C-to-T nucleotide (nt) substitution at the +6 position in exon 7 of SMN2 leads to this exon being skipped ∼85% of the time (4), resulting in an inactive SMN isoform. This C-to-T substitution facilitates the preferential binding of a splicing inhibitor, heterogeneous nuclear ribonucleoproteins (hnRNP) A1 (5), over a splicing activator, serine and arginine rich splicing factor 1 (SRSF1) (6). Importantly, the binding of hnRNP A1 shifts the splicing pattern from exon 7 inclusion to a skipped phenotype. With reduced levels of functional SMN protein, the size of motor neurons in the patients’ spine is significantly smaller than those in healthy individuals, eventually causing muscle weakness (1).

**Figure 1.**
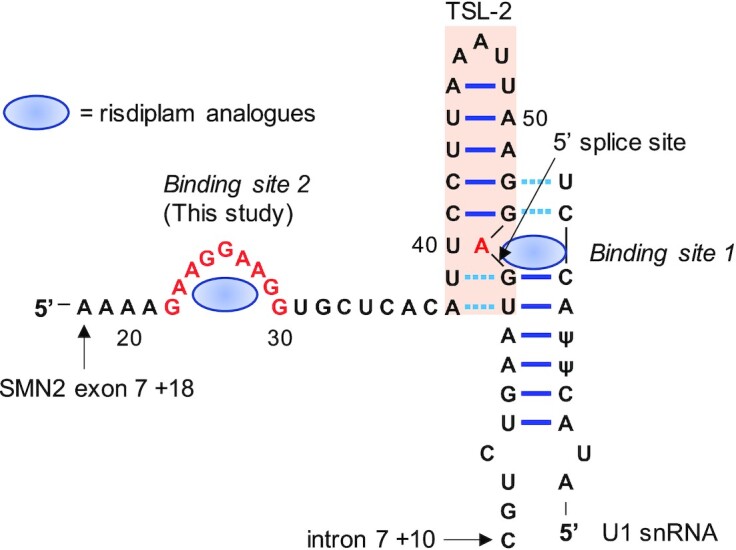
Risdiplam's dual binding sites in exon 7 of SMN2 pre-mRNA. One of the risdiplam analogues, SMN-C5, binds to site 1 by stabilizing a bulged A (exon 7 +54) between the 5′-splice site and U1 snRNA and subsequently enhances the U1 snRNP recruitment (15). Ψ = pseudouridine. Terminal stem-loop (TLS2) is an inhibitory *cis*-acting regulatory element for exon 7 splicing (exonic splicing silencer). The SMN2 pre-mRNA sequence from exon 7 + 18 to intron 7 + 10 is shown.

A promising therapeutic strategy is to restore proper splicing of the SMN2 exon 7 to compensate for the loss of the SMN1 gene in SMA patients (2). Following this strategy, there are two existing FDA-approved therapeutics for SMA, namely nusinersen (Spinraza) (7) and risdiplam (8). Nusinersen is an antisense oligonucleotide (ASO), which acts by direct binding to the SMN2 pre-mRNA with Watson-Crick base-pairing. The binding site for nusinersen (intron 7 +10 to +27 in SMN2) was identified as an intronic splicing silencer (ISS) (9). Blocking this ISS by nusinersen promotes inclusion of exon 7 (9). On the other hand, risdiplam is a first-in-class small-molecule splicing modifier that increases the production of full-length SMN2 mRNA upon oral administration in the SMA mouse model (10) and in humans (11). It was demonstrated that analogues of risdiplam bind directly to exon 7 of the SMN2 pre-mRNA at two separate locations: binding site 1 is located at the 5′ splice site (12,13) and binding site 2 is a GA-rich sequence located ∼24 nts upstream of the 5′ splice site (Figure 1). There is a stable splicing-inhibitory RNA element, terminal stem-loop (TSL) 2 between the two putative risdiplam-binding sequences (12,14). The existence of TSL2 likely makes the two putative binding sites closer in space (Figure 1).

**Figure 2.**
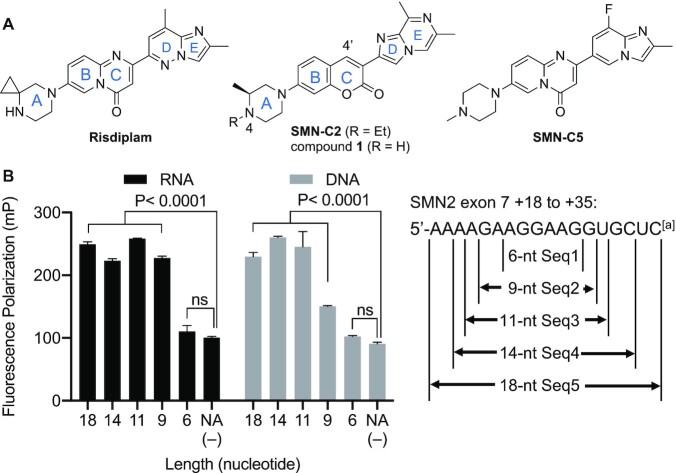
(**A**) The structures of the risdiplam and its active analogues, SMN-C2 and SMN-C5. (**B**) The fluorescence polarization (FP) of SMN-C2 (0.5 μM) and RNA and DNA sequences (50μM) of various lengths. NA (–) = no nucleic acid added. All data were reproduced in triplicates. The adjusted *P* values were calculated by Dunnett's test (ns = not significant). *^a^*In the DNA sequences, deoxyribonucleotides, dA, dT, dG and dC, were used in substitution of A, U, G and C in RNA.

At binding site 1, one of the risdiplam analogues, SMN-C5 (Figure 2A), was found to stabilize the formation of a ternary complex of the 5′ splice site of the SMN2 exon 7 and the U1 small nuclear ribonucleoprotein (snRNP) via binding to a bulged A (15) (Figure 1). Without SMN-C5, the 5′ splice site–U1 snRNA duplex is not stable, and the formation of TSL2 is more favourable (15).

At binding site 2, we previously demonstrated that a 15-nt synthetic single-stranded (ss) RNA that contains the GA-rich sequence selectively binds to SMN-C2 (12). Binding site 2 was also identified as part of the exonic splicing enhancer (ESE) 2 (13). The biological consequences for risdiplam analogue binding to binding site 2 include alterations in binding of trans-acting splicing regulatory proteins. Treatment with a risdiplam analogue, SMN-C3 (10), enhanced binding between a 500-nt pre-mRNA sequence containing the GA-rich sequences and a splicing regulatory protein, far upstream element-binding protein 1 (FUBP1) (12). In surface plasmon resonance (SPR) assays, the presence of SMN-C5 prevents the binding of hnRNP G with ESE2 (13). Reverse-genetic studies showed that deletion of the entire ESE2 sequence in a cell-based minigene system reduces the effectiveness of SMN-C5 at restoring proper splicing of SMN2 (13). The effective concentration at 50% potency (EC_50_) of SMN-C5 significantly increases in ΔESE2 minigene compared to the wildtype sequences, and SMN-C5 failed to induce the complete inclusion of exon7 even at a high concentration (10 μM) (13). However, if only the GA-rich sequence is removed, SMN-C5 can still weakly modulate splicing of the SMN2, indicating that the GA-rich sequence is not strictly required for drug action (13). These findings indicated that the GA-rich sequence plays a role in enhancing the drug potency. GA-rich sequences are also found in some other risdiplam-sensitive genes, such as STRN3 (13,16). The GA-rich sequence in the STRN3 gene also found substantial binding to SMN-C5 by the SPR assay (13).

In view of this, it was hypothesized that the 5′ splice site is the primary target for the modulatory activity, and that ESE2 facilitates the modulator binding through a cooperative tertiary RNA structure with the 5′ splice site (13). However, because biophysical evidence supporting the existence of such tertiary RNA structure has not been found (12), and because it is not uncommon that small molecules are able to bind to several different sites in large RNAs (17–19), we, therefore, suggest that the two small-molecule binding sites should probably be treated separately rather than as parts of a larger more complicated binding site.

In this study, we interrogated the affinity and selectivity of risdiplam analogues that bind to the GA-rich sequence (binding site 2) in SMN2 exon 7 using a range of biochemical and biophysical tools. Instead of deleting the whole ESE2 sequence, we made single-point mutations within the GA-rich sequence that could disrupt the binding between risdiplam analogues and this GA-rich sequence. Molecular dynamics (MD) has proven useful in simulations of the structural dynamics of nucleic acid (20,21), notably nucleic acid–ligand interactions (22–25). We performed all-atom simulations using a robust Gaussian accelerated MD (GaMD) (26,27) method to obtain new insights into the mechanism of risdiplam analogue binding to the target nucleic acids.

## MATERIALS AND METHODS

### Reagents

All synthetic DNA and RNA oligomers were purchased from Integrated DNA Technologies and reconstituted in nuclease-free water (Invitrogen #AM9932) at 1 mM. The concentration of the oligonucleotides at ∼0.1 mM was calibrated with the A260 value measured by a NanoDrop ND-1000 (Thermo, Waltham, MA, USA) and the predicted extinction coefficient (http://www.oligoevaluator.com) using Lambert–Beer's Law. The risdiplam analogues (1 mM in DMSO) were diluted in 2× assay buffer (40 mM HEPES, 200 mM NaCl for RNA; 40 mM HEPES, 200 mM NaCl, 2 mM MgCl_2_ for DNA) to make a 1 μM 2× working solution.

### Fluorescence polarization binding assay

A 1:2 dilution series (8 points) of each oligonucleotide was prepared in 20–30 μl water to desired concentrations (i.e. 1–128 μM). 20–30 μl 2× working solution containing the 2× assay buffer and the small-molecule ligand was added to each oligonucleotide sample in 1:1 (v/v) and mixed by pipetting for 4 times. For fluorescence polarization measurement, 20 μl of the above 1× working solution was transferred into a 384-well, black, flat-bottom microplates (Greiner Bio-One) in duplicates or triplicates. The plate was equilibrated at room temperature for 5 min before being read using a microplate reader (SYNERGY H1, BioTek; excitation/emission = 360/460 nm) at 25°C. For compound SMN-C5, a competitive binding assay was employed to measure an apparent *K*_d_. SMN-C5 was 1:2 serial diluted for 10 concentration points starting at 750 μM in DMSO. A mixture of SMN-C2 (0.5 μM) and DNA Seq6 (25 μM) in 1× assay buffer was prepared and incubated at room temperature for 10 min. 3 μl of SMN-C5 solution at each concentration was added to a 42 μl SMN-C2/DNA Seq6 mixture and incubated at room temperature for 10 minutes. The mixture was then transferred into a microplate (20 μl each well) in duplicates for fluorescence polarization readout. The experimental data were analysed using Prism 8 software (Graphpad Software, San Diego, CA, USA). The dissociation constant (*K*_d_) was calculated with 95% confidence interval after nonlinear curve fitting (Sigmoidal, 4 parameters).

### Gaussian accelerated molecular dynamics (GaMD) simulations

GaMD works by adding a harmonic boost potential to smooth the potential energy surface and reduce system energy barriers. GaMD provides unconstrained enhanced sampling without the requirement of pre-defined collective variables. Moreover, because the boost potential exhibits a Gaussian distribution, biomolecular free energy profiles can be properly recovered through cumulant expansion to the second order (27). GaMD has been demonstrated to accelerate biomolecular simulations by orders of magnitude, especially for ligand binding to proteins (28,29). Here, we have applied GaMD to explore the binding of the risdiplam analogue to the putative target nucleic acids with GA-rich sequence. The simulation structures of nucleic acids were built using NAB in the AmberTools package (30). The nucleotide sequence was used to construct the Arnott B-Right handed DNA and RNA duplexes. One of the strands from each duplex was extracted to generate the starting nucleic acid structure (Seq6) of DNA and RNA. A ligand molecule of compound **1** was placed randomly at >15 Å away from the nucleic acid. Each simulation system was then prepared using the solution builder plug-in with the CHARMM-GUI web server (31). Each system was solvated in 0.1 M NaCl solution at 298.15 K. The AMBER force field, BSC1 was used for DNA, OL3 for the RNA, GAFF2 for ligand and TIP3P for water in the system.

Initial energy minimization, equilibration, and conventional molecular dynamics (cMD) of compound **1** binding to the DNA and RNA Seq6 were performed using the output files from CHARMM-GUI. Specifically, the system was energy minimized using the steepest descent for 2500 steps and conjugate gradient for another 2500 steps. After minimization, the system was heated from 0 to 298.15 K in 125 ps simulation by applying 1.0 kcal/(mol•Å^2^) harmonic position restraints to DNA/RNA and ligand heavy atoms with a constant number, volume and temperature (NVT) ensemble. The system was further equilibrated for 125 ps at 298.15 K using constant number, pressure and temperature (NPT) ensemble with the same restraints as in the NVT run. Then cMD without any constrains was performed to further relax the system for 1 ns at 1 atm pressure and 298.15 K temperature.

The simulations proceeded with GaMD (see method details in SI), which was implemented in the GPU version of AMBER18 (32). A short cMD run of 2 ns was performed to collect potential statistics (including the maximum, minimum, average and standard deviation (SD)). Then 40 ns GaMD equilibration after applying the boost potential was performed. All GaMD simulations were run at the ‘dual-boost’ level by setting the reference energy to the lower bound; i.e., E = V_max_ (26,27). One boost potential is applied to the dihedral energetic term and another to the total potential energetic term. The average and SD of the system potential energies were calculated every 200 000 steps (400 ps) for both systems. The upper limit of the boost potential SD, σ0 was set to 6.0 kcal/mol for both the dihedral and the total potential energetic terms for DNA–ligand binding system. While the σ0 was set to 3.0 kcal/mol and 6.0 kcal/mol for the total and dihedral potential energetic terms in the RNA-ligand binding system, respectively. Finally, five independent 500 ns dual-boost GaMD production simulation runs were conducted with randomized initial atomic velocities. The boost potential was applied to the total and dihedral potential energies of the system. The GaMD production simulations are summarized in Supplementary Table S1. In all simulations, the hydrogen-heavy atom bonds were constrained using the SHAKE algorithm and the simulation time step was set to 2.0 fs. The particle mesh Ewald (PME) method was employed to compute the long-range electrostatic interactions and a cutoff value of 9.0 Å was applied to treat the non-bonded atomic interactions. The temperature was controlled using the Langevin thermostat with a collision frequency of 1.0 ps^–1^.

Trajectory analysis was carried out using VMD (33) and CPPTRAJ (34). Hierarchical Agglomerative clustering in CPPTRAJ was performed on the whole system consisting of both the DNA/RNA and ligand. Representative conformations of each system were identified from the top 10 structural clusters. The centre-of-mass distance between the ligand and DNA/RNA and the DNA/RNA radius of gyration *(Rg)* were calculated using CPPTRAJ. They were also used as reaction coordinates for calculating a 2D PMF free energy profile by reweighting all five GaMD simulations combined (see method details for energetic reweighting of GaMD simulations in SI). The PyReweighting toolkit (35) was used for reweighting the GaMD simulations. A bin size of 1 Å was used for distance and *Rg* and cutoff set to 500 frames in a bin or cluster for reweighting.

### NMR studies

NMR spectra were acquired on a Bruker 600 MHz Avance III HD spectrometer equipped with a TCI cryoprobe, and a Varian INOVA 600 MHz spectrometer equipped with a cryogenic HCN probe. To a 1 ml Eppendorf tube was added 500 μM compound **1** solution (20 μl for RNA samples, 4 μl or 40 μl for DNA samples), followed by 20 μl 10× PBS pH 5.0 buffer, 10 μl D_2_O, 2 μl DMSO-d_6_ and H_2_O. The solution was then heated at 60°C for 1 min before 1 mM RNA Seq6 or DNA Seq4 solution was added to form a 200 μl small molecule-nucleic acid solution. The amount of the RNA Seq6 or DNA Seq4 solution and H_2_O were varied to form solutions of different ratios of RNA Seq6 or DNA Seq4 to compound **1**. The series of solutions with 5 , 10, 20 mol% RNA Seq6, or 2.5, 5, 10, 20, 50, 100, 200, 400, 1000, 2000, 5000 and 8200 mol% DNA Seq4 were then transferred to 3 mm NMR tubes for analysis. ^1^H NMR spectra were acquired at 25°C with excitation sculpting (ES) or presaturation to suppress the water signals. The STD experiment was performed with saturation at 5.80 ppm for DNA and at 5.70 ppm for RNA (in both cases the middle of the spectral region of nucleotides’ anomeric protons was chosen). A train of selective gaussian pulses with a bandwidth of 125 Hz and 2 s mixing time were utilized, with 16k scans acquired for DNA and 32k scans for RNA samples.

## RESULT

### The minimum required sequence for SMN-C2 binding

We previously reported that a risdiplam analogue, SMN-C2 (Figure 2A), binds to a 15-nt GA-rich sequence in SMN2 exon 7 (12). SMN-C2 was observed to bind to GA-rich DNAs similarly to RNAs (Figure 2B). Here, we first investigated the minimum length of sequence required for SMN-C2 binding. Fortunately, the fluorescent coumarin moiety of SMN-C2 (ring B/C) permits the use of a fluorescence polarization (FP) assay to interrogate said binding. Applying different-length RNAs and DNAs at 50 μM, we found that a 9-nt sequence (i.e. GAAGGAAGG) is the minimum length required for SMN-C2 binding (Figure 2B). To explore the tolerable and preferential binding sequences for SMN-C2 binding, we added a single overhang nucleotide at both the 5′ and the 3′ ends of a cohort of 9-nt RNA/DNA sequences (Table 1), because the overhang nucleotides may provide extra stability to the nucleic acid–SMN-C2 complex (Supplementary Table S2, Supplementary Figure S4). The U/T was chosen to flank the 9-nt sequence because we previously reported that SMN-C2 has the least binding affinity to the pyrimidines (12). Replacing either end of the 9-nt core sequence with one or two ‘inactive’ Us in RNA (or Ts in DNA), significantly weakened the SMN-C2-binding affinities (Table 1, Seq6 versus Seq7–9), thus validating that the 9-nt GA-rich sequence is the minimum length required for SMN-C2 binding.

**Table 1.** Binding affinities^a^ of SMN-C2 and ssDNAs^b^ or ssRNAs, which harbour a 9-nucleotide GA-rich sequence

Seq	Sequence^b,e^	RNA *K*_d_ (μM)	DNA *K*_d_ (μM)
6^b^	UGAAGGAAGGU	17.5 ± 2.2	3.1 ± 0.7
7^b^	UUAAGGAAGGU	42.3 ± 13.3	>100
8^b^	UGAAGGAAGUU	>100	14.7 ± 0.7
9^b^	UUAAGGAAGUU	>100	>100
10^b^	UAAAGGAAGGU	>100	>100
11^b^	UGGAGGAAGGU	N.T.^c^	57.3 ± 20.0
12^b^	UGAAAGAAGGU	>100	17.6 ± 3.4
13^b^	UGAAGAAAGGU	>100	>100
14^b^	AGAAGGUAGGU	9.9 ± 0.8	>100
15^b^	UGAAGGAUGGU	9.4 ± 0.5	41.1 ± 4.4
16^b^	UGAAGGAAAGU	77.0 ± 26.0	27.4 ± 6.0
17^b^	UGAAGGAAGAU	48.2 ± 9.0	56.4 ± 10.4
18^d^	Binding site 1 sequence	60 ± 36	N.T.^c^

^a^Determined by the fluorescence polarization assay with 0.5 μM SMN-C2. The *K*_d_ range is the 95% confidence interval of the calculated 50% response concentration from the Sigmoidal interpolation. All dose titrations were reproduced in three replicates.

^b^In DNA sequences, deoxyribonucleotides, dA, dT, dG and dC, were used in substitution of A, U, G and C for RNA.

^c^N.T. = not tested.

^d^For comparison, a 1:1 annealed mixture of the U1 snRNA binding sequence, 5′-AUACΨΨACCU (Ψ = pseudouridine) and the 5′ splice site, 5′-GGAGGUAAGUCU to resemble binding site 1 (15).

^e^The underlined positions indicate the single-point nucleotide differences from the 9-nt putative binding sequence.

Next, we mutated the 9-nt core nucleotides to investigate the sequence specificity of SMN-C2 binding. Importantly, some single-point mutations of the core sequence as shown in Table 1 (Supplementary Figure S1) resulted in a 3-fold decrease in binding affinity even by merely replacing some single G into A or vice versa, highlighting the specificity of SMN-C2 binding (Table 1, Seq10–13, 16, 17). After investigating the binding of SMN-C2 with an additional 25 sequences in the context of 18-nt DNA, we identified the complete consensus sequence for SMN-C2 as GARGGARGG (R = A/G) for DNA (Supplementary Table S3). The sequence requirement in RNA for SMN-C2 seems less stringent than that in DNA, as replacing some single A into U retains the binding affinity (Table 1, Seq14, 15). Double-stranded (ds) RNA Seq18 represents the duplex formed between the 5′ splice site of SMN2 exon 7 and U1 snRNA, and was previously used in NMR studies for binding site 1 (15). Comparing the two risdiplam putative binding sites side-by-side, the binding affinity (*K*_d_) of SMN-C2–RNA Seq6 (binding site 2) is 3.4-fold stronger than that observed with RNA Seq18 (Table 1). The binding affinity of SMN-C2–dsRNA Seq18 determined by the FP assay (*K*_d_ = 60 ± 36 μM, Table 1) is generally consistent with the literature, where the affinity of this dsRNA to another risdiplam analogue, SMN-C5, was determined as *K*_d_ = 28 ± 9 μM in an NMR chemical shift assay (15).

### Mutations in the GA-rich sequence reduced the drug effect in cells

To evaluate if the GA-rich sequence facilitates the activity of risdiplam analogues, two single-point mutations from Table 1 were individually introduced in the cell-based minigene system (modified from pCI-SMN2 plasmid (4), see Supporting Information): (i) exon 7 +22G>T (M1, mutation in Seq7) and (ii) +22G>A (M2, mutation in Seq10). In 293T cells that were transfected with the mutated minigenes, SMN-C2 can still significantly rescue exon 7 inclusion (> 90%) at 1 μM (Supplementary Figure S6). However, the EC_50_ was ∼2–3-fold higher than that observed with the minigene with a wildtype exon 7 sequence, indicating that GA-rich sequence is relevant to the mechanism of SMN-C2 and is pivotal to maintaining the potency of SMN-C2. Compared to the previous reverse genetic studies that deleted the whole ΔESE2 sequence (13), these results demonstrated that even a single-point mutation in the GA-rich sequence is sufficient to lessen the drug effect. This is consistent with the hypothesis that the GA-rich sequence serves as a secondary RNA target that facilitates SMN2 exon 7 splicing (13).

### SMN-C2 and ssDNA formed a 1:1 complex

**Figure 3.**
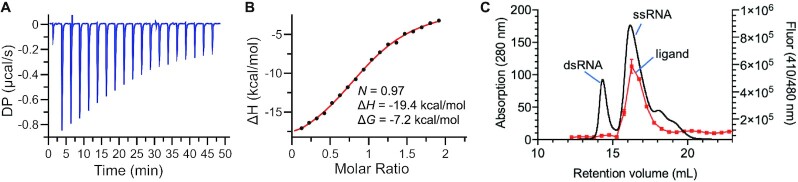
ITC (**A**) raw differential power (DP) and (**B**) integrated data for SMN-C2 (250 μM) and DNA Seq6 (25 μM) in a buffer that contains 30 mM 2-(*N*-morpholino)ethanesulfonic acid (MES, pH 6.1), 5% DMSO, and 100 mM NaCl. (**C**) Size-exclusion chromatography with a Superdex 75 column for an annealed mixture of RNA Seq4 (150 μM), RNA Seq4_RC (50 μM) and SMN-C2 (100 μM) in 1× phosphate buffered saline (PBS) with the absorption (black, absorption at 280 nm for RNA) and fluorescence readout (red, fluorescence at excitation/emission = 410/480 nm for SMN-C2). The figures are the representation of three independent experiments.

We first measured the stoichiometry and reconfirmed the binding affinity of DNA Seq6 and SMN-C2 by isothermal calorimetry (ITC). They showed a 1:1 binding stoichiometry with a calculated *K*_d_ of 4.8 μM (Figure 3A and B), which is close to the *K*_d_ observed with the FP assay (Table 1, DNA Seq6). Interestingly, a dsDNA formed by annealing Seq6 and its reverse complement, Seq6_RC, abrogated most of the binding affinity (Supplementary Figure S7). In the same experimental settings, RNA Seq6 generated significant solvation heat, which made us unable to obtain an accurate ITC measurement. Second, the possibility of RNA or DNA oligomerization (e.g. G-quadruplex formation) was ruled out by size-exclusion chromatography. The retention volume for the 14-nt RNA or DNA (Seq4) was consistent with its reverse complement (Seq4_RC) and was well resolved from the annealed double-stranded RNA or DNA (Seq4 + Seq4_RC), in which the molecular weight almost doubles. In addition, SMN-C2 did not significantly change the retention volume of the RNA or DNA Seq4 (Figure 3C and Supplementary Figure S8). The results suggested that although Seq4 contains six Gs, it cannot form a G-quadruplex at 100 μM in the presence or absence of SMN-C2. An 11-nt DNA sequence that contains two GGG segments (Seq19 = TGAGGGAGGGT) can however form an intermolecular G-quadruplex at 100 μM as the retention volume in size-exclusion chromatography was similar to that observed with a random 45-nt ssDNA (Supplementary Figure S9). In RNA, replacing a G into an A in Seq6 induced intermolecular G-quadruplex formation (Seq11 = UGGAGGAAGGU, Supplementary Figure S10). Importantly, in the presence of dsRNA Seq4 (annealed Seq4 and Seq4_RC), the elution of SMN-C2 was concurrent with the ssRNA but not the dsRNA peak (Figure 3C). The co-elution of a mixture of SMN-C2 and single-stranded and double-stranded DNA Seq4 in size-exclusion chromatography also showed similar results (Supplementary Figure S8B), reconfirming that SMN-C2 preferentially binds to the single-stranded over the double-stranded Seq4. Collectively, our results suggested that SMN-C2 binds to the monomeric single-stranded GA-rich sequences in a 1:1 ratio.

### GaMD simulations suggested a mechanism for small-molecule–nucleic acid binding

**Figure 4.**
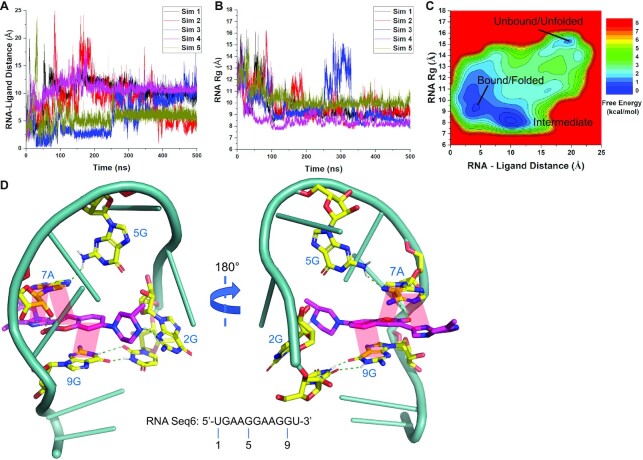
GaMD simulations revealed spontaneous binding of compound **1** to RNA Seq6. (**A**) The center-of-mass distance between RNA–ligand and (**B**) the RNA radius of gyration (*R_g_*) plotted as a function of simulation time. (**C**) 2D free energy profile calculated with all five GaMD simulations combined, in which three low-energy states were identified, namely the Unbound/Unfolded, Intermediate and Bound/Folded. (**D**) Representative conformation of compound **1**-bound RNA Seq6 in the folded state. Magentas = compound **1**, yellow = interacting nucleobases, cyan = other nucleotides, green dashed line = polar interaction, light red shade = π–π stacking.

All-atom GaMD simulations were used to study the molecular interactions responsible for the binding of compound **1** (Figure 2A) to the putative nucleic acid target sequence. RNA and DNA Seq6 were chosen because they represent one of the best nucleic acid binding receptors for risdiplam analogues (Table 1). Compound **1**, a more water-soluble analogue of SMN-C2, is used in simulations for being consistent with the NMR experiments (see below). During five independent 500 ns GaMD production simulations, compound **1** appeared to spontaneously bind to both RNA (Supplementary Movie S1) and DNA Seq6 (Supplementary Movie S2). When bound to RNA Seq6, the centre-of-mass distance between RNA and ligand reduced to ∼6 Å (Figure 4A) and RNA radius of gyration (*R*_g_) reduced to ∼8.0 Å (Figure 4B). A 2D free energy profile was then calculated through reweighting of the five GaMD simulations combined. Three low-energy conformational states of the system were identified (Figure 4C), for which structural clustering was performed to obtain the representative system conformations, including the ‘Unbound/Unfolded’ (Supplementary Figure S11B), ‘Bound/Intermediate’ (Supplementary Figure S11A) and ‘Bound/Folded’ states (Figure 4D). Compound **1** was able to interact with RNA Seq6 in two bound states. In the Bound/Folded state, compound **1** bound to folded RNA, forming a compact structure (Figure 4D). Two successive GAAG loop-like structures (36,37) were identified in the folded RNA for binding of the compound **1**.

**Figure 5.**
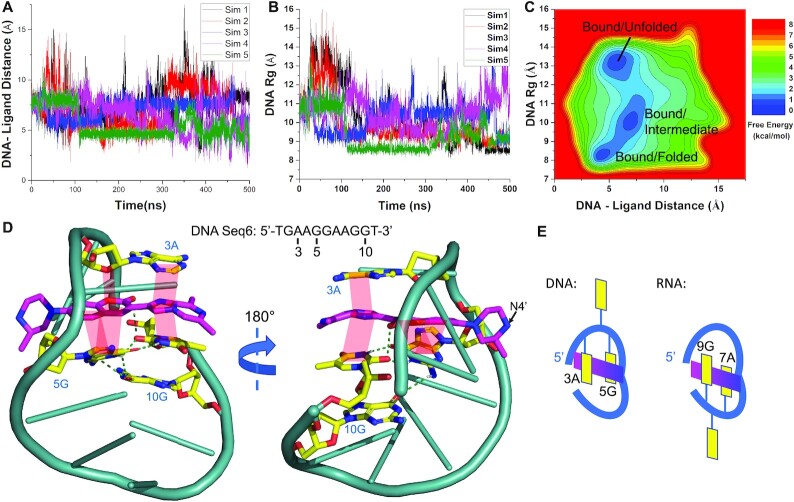
GaMD simulations revealed spontaneous binding of compound **1** to DNA Seq6. (**A**) The centre-of-mass distance between DNA–ligand and (**B**) the DNA radius of gyration (*R*_g_) plotted as a function of simulation time. (**C**) 2D free energy profile calculated with all five GaMD simulations combined, in which three low-energy states were identified, namely the Bound/Unfolded, Bound/Intermediate and Bound/Folded. (**D**) Representative conformation of compound **1**–bound DNA Seq6 in the folded state. Magentas = compound **1**, yellow = interacting nucleobases, cyan = other nucleotides, green dashed line = polar interaction, light red shade = π–π or lone pair-π stacking. (**E**) A sketch illustrating the different binding locations of the small molecule (magentas) in DNA and RNA Seq6.

In GaMD simulations of the interaction of compound **1** and DNA Seq6, the location of the bound small molecule was slightly different from that observed in RNA, as demonstrated by the centre-of-mass distance between DNA and ligand being reduced to ∼4 Å in the bound states (Figure 5A). In these states, the DNA *R*_g_ reduced to ∼8.0 Å, similar to that observed for RNA (Figure 5B). Three low-energy conformational states of the DNA-ligand system were also identified for DNA Seq6 (Figure 5C, Supplementary Movie S2), including the ‘Bound/Unfolded’ (Supplementary Figure S12A), ‘Intermediate’ (Supplementary Figure S12B) and ‘Bound/Folded’ states (Figure 5D). In the Bound/Folded state, a similar binding mode of compound **1** was observed in DNA as in the RNA with subtle differences. In DNA, the coumarin core (ring B/C) of compound **1** intercalated between the second and fourth bases of the first GAAG motif, whereas in RNA, the intercalation occurred between the second and fourth bases of the second GAAG motif (Figure 5E). In both RNA and DNA, it appeared that the AAG trinucleotide in the GAAG motif is thus important for binding of compound **1** through π-stacking interactions. Even though both RNA and DNA Seq6 contained two GAAG motifs, the nucleic acids formed compact sequential loop-like structures (Figures 4D and 5D), which could accommodate only one small molecule. Furthermore, the unfolded structure of RNA Seq6 appeared to be more flexible than the unfolded DNA, and compound **1** did not spontaneously bind to the unfolded RNA (Supplementary Figure S11B).

### NMR studies of compound 1 binding to single-stranded nucleic acids

**Figure 6.**
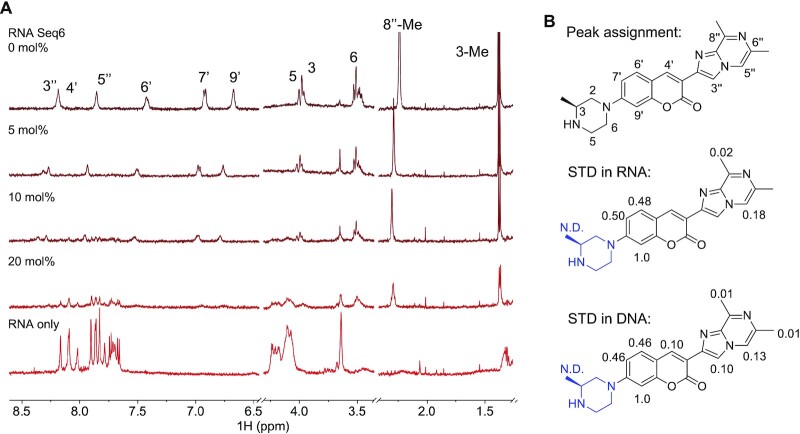
(**A**) ^1^H NMR titration of compound **1** (50μM) with RNA Seq6 at various concentrations, from top to bottom: 0 mol% (no RNA), 5 mol% RNA, 10 mol% RNA, 20 mol% RNA, and a control spectrum of RNA (50 μM) without compound **1**. (**B**) The numbering of compound **1** (top) and the relative saturation-transfer difference (STD) at different position of compound **1** in a mixture of RNA Seq6 (2.5 μM) and compound **1** (50 μM) solution (middle) or DNA Seq4 (0.5μM) and compound **1** (10 μM) solution (bottom). STD was not detected (N.D.) in the whole piperazine ring (ring A, blue). STDs at 4′, 3’’ and 6’’-Me in the RNA Seq6 sample were not measured because the line-broadening effects.

We performed a series of NMR experiments to validate the simulated binding modes of compound **1** to the target nucleic acid. First, RNA Seq6 was titrated into a solution containing 50 μM compound **1** and the ^1^H NMR spectra were measured. The peaks from compound **1** shift upon the addition of 5 mol% RNA and broaden as the concentration of RNA increases to 20 mol% (Figure 6A, Supplementary Figure S13). The line-broadening effect is similar to the reported observation for the binding of SMN-C5 and the dsRNA of the 5′-splice site of SMN2 exon 7 and U1 snRNA (Seq18) (15). Specifically, aromatic signals of compound **1** (6.6–8.5 ppm) are below the detection limit when 20 mol% of the RNA Seq6 is present, whereas the aliphatic signals are still observed (1.3–4.1 ppm, Figure 6A). The peak width at half maximum plus the *J* coupling constant (FWHM + *J*) of the doublet for 3-CH_3_ of compound **1** (∼1.4 ppm) only increases from 9.1 Hz at 0 mol% RNA to 11.8 Hz at 20 mol% RNA (Figure 6A). The reduced attenuation of the aliphatic signals relative to aromatic signals upon addition of RNA Seq6 suggested that the piperazine ring (ring A) retains dynamics similar to the free ligand, whereas the rings B/C and D/E exhibit behaviour associated with nucleic acid binding. In titration of DNA Seq4, similar line-broadening effects were observed (Supplementary Figure S14). At 20 mol% DNA Seq4, the piperazine ring also showed the least attenuation of the NMR signals (Supplementary Figure S14), consistent with the RNA data. The NMR titration also suggested that a heterogeneous binding conformation exists, because there were no bound-form compound **1** peak reappearing even if an 82-fold excess DNA Seq4 was added (38) (Supplementary Figures S15 and S16).

Next, saturation transfer difference (STD) experiments were carried out to further interrogate the interaction between compound **1** and RNA Seq6 or DNA Seq4 (Figure 6B) (39). The anomeric proton resonances of the nucleotides in RNA Seq6 and DNA Seq4 were selectively saturated at 5.70 ppm and 5.80 ppm, respectively, by a train of gaussian pulses with a bandwidth of 125 Hz, and STD signals of compound **1** were measured (Supplementary Figures S17 and S18). The STD results in RNA and DNA are highly consistent. The aromatic rings B/C and D/E in compound **1** demonstrated strong STD, while the piperazine moiety yielded no observable STD peaks. The 6′- and 8′- methyl groups (ring E) also showed small STD signals compared to those observed with the aromatic protons. Thus, both the ^1^H NMR titration and the STD experiments strongly indicated that the interaction between compound **1** and the DNA Seq4 is mainly driven by the aromatic moieties, specifically rings B, C, and D, of this small molecule. This result is also consistent with the fact that SMN-C2 prefers to bind with purine-rich sequences since the latter engage in π-interactions more effectively than pyrimidine-rich sequences (40).

### Structure–affinity-relationship studies of risdiplam analogues

**Scheme 1.**
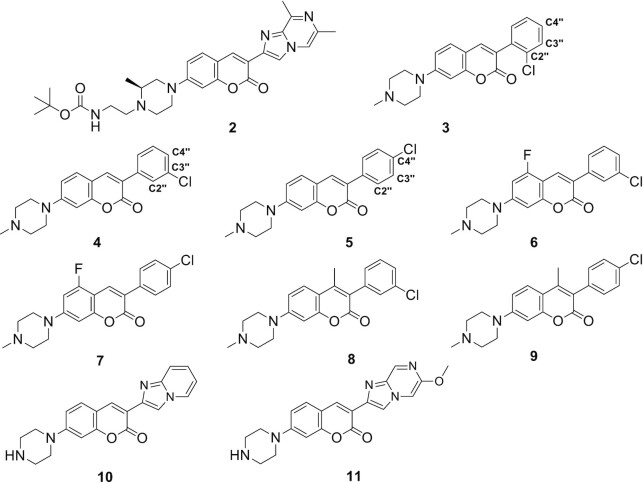
The structures of new risdiplam analogues.

To further probe the nucleic acid interactions with risdiplam analogues, we set out to alter the small-molecule structures. Specifically, we compared two known active risdiplam analogues, SMN-C2 and -C5 (Figure 2A) (41), and synthesized additional 11 risdiplam analogues containing the coumarin core (Scheme 1). In this cohort of compounds, only SMN-C5 contains a pyridopyrimidinone core (ring B/C), instead of a coumarin core, which is not fluorescent. Utilizing competitive FP and surface plasmon resonance (SPR) assays (Supplementary Figure S19), we observed that SMN-C5 demonstrated similar binding affinities to those observed with SMN-C2 (Table 2, entries 2 and 3). In the newly synthesized collection of risdiplam analogues (Table 2, Scheme 1, Supplementary Figure S2), removing the substituents on the N4 position of the piperazine ring (compound **1**, ring A) does not substantially alter the binding affinity with RNA and DNA Seq6 (Table 2). Extending N4 with a bulky butyloxycarbonyl (Boc) group (compound **2**) did not reduce the binding affinity to DNA Seq6 significantly (Table 2). Interestingly, the Boc group reduced the binding of compound **2** to RNA Seq6 by ∼3-fold, suggesting that ligand binding mode between DNA and RNA Seq6 may have some differences (Table 1, RNA Seq6 versus Table 2, entry 6).

**Table 2.** Binding affinities of the risdiplam analogues^a^ and the GA-rich DNA and/or RNA sequences

#	Compounds	*K*_d_ (μM) RNA	*K*_d_ (μM) DNA	SMN2 EC_50_ (μM)^e^
1	SMN-C2	5.4^b^	3.1 ± 0.7^c^	0.26 ± 0.13
2	SMN-C5	8.0^b^	5.8 ± 2.5^c^	0.059 ± 0.009
4	compound **1**	18.5 ± 5.4^d^	2.9 ± 0.3^c^	0.014 ± 0.003
5	compound **2**	51.2 ± 4.9^d^	4.0 ± 0.3^c^	>8
7	compound **3**	>100^d^	>100^c^	>8
8	compound **4**	35.9 ± 14.8^d^	13.9 ± 1.1^c^	>8
9	compound **5**	30.6 ± 12.3^d^	8.2 ± 0.7^c^	>8
10	compound **6**	32.8 ± 5.6^d^	18.7 ± 4.0^c^	>8
11	compound **7**	30.9 ± 6.7^d^	9.9 ± 0.9^c^	>8
12	compound **8**	>100^d^	>100^c^	>8
13	compound **9**	>100^d^	>100^c^	>8
14	compound **10**	14.5 ± 2.3^d^	2.8 ± 0.5^c^	>8
15	compound **11**	11.4 ± 0.7^d^	1.4 ± 0.2^c^	>8

^a^For structures of entries 4–15, see Scheme 1. For FP experiments, the *K*_d_ range is the 95% confidence interval of the calculated 50% response concentration from the Sigmoidal 4PL interpolation. All dose titrations were reproduced in three replicates.

^b^The *K*_d_ values were measured using the SPR assay for RNA Seq4.

^c^The *K*_d_ values were measured using the FP assay for DNA Seq6.

^d^The *K*_d_ values were measured using the FP assay for RNA Seq6.

^e^The EC_50_ of the cell-based SMN2 splicing assay in 293T cells (56).

We also truncated the bicyclic ring D/E (see Figure 2A for assignment) in SMN-C2 into monocyclic structures with various substituents. The position of the Cl substituent on the monocyclic ring D is crucial for the binding affinity to DNA Seq6 (compounds **3–5**). Changing the Cl position from C4" into C2" reduces the binding affinity by more than 12-fold (compounds **3–5**, Table 2), probably because the 2"-Cl forces ring D out of coplanarity. The binding affinity to RNA Seq6 is less sensitive to the positions of Cl substituents (compounds **3–5**, Table 2). In general, the analogues with a monocyclic ring D have weaker binding affinities to both RNA and DNA Seq6 than that observed with SMN-C2 (Table 1, DNA Seq6 vs Table 2, compounds **3–9**). Unlike these compounds with a monocyclic ring D, the new risdiplam analogues with a bicyclic ring D/E (compounds **10** and **11**) both showed high binding affinity to the GA-rich sequence RNA and DNA Seq6 when the ring D is unchanged from SMN-C2 (compounds **10** and **11**, Table 2).

### RNA secondary structures enhanced the loop-like conformation and SMN-C2 binding

**Scheme 2.**
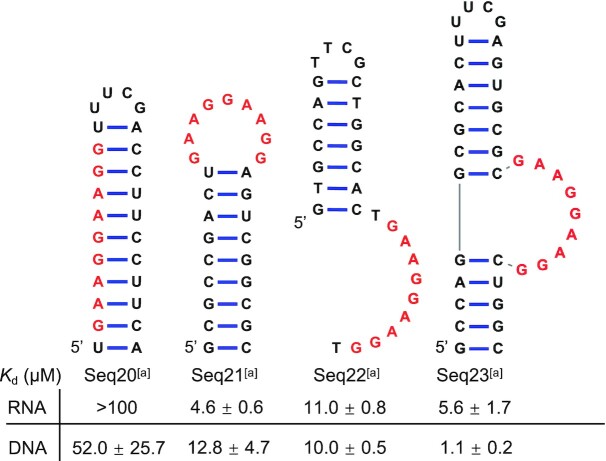
Various RNA or DNA secondary structures that harbour the SMN-C2 putative binding site (highlighted in red). ^a^In DNA sequences, deoxyribonucleotides, dA, dT, dG and dC, were used in substitution of ribonucleotides A, U, G and C for RNA. The *K*_d_ range is the 95% confidence interval of the calculated 50% response concentration from the Sigmoidal 4PL interpolation. All dose titrations were reproduced in three replicates.

To further validate the double loop-like conformation in SMN-C2 binding, we synthesized several oligonucleotides that can stabilize or destabilize the folded conformation of the core sequence by base-pairing (Scheme 2, Supplementary Figure S3). As expected, when additional nucleotides complementary to the core sequence are appended to the 3′-end (DNA and RNA Seq20), the putative binding sequence in the stem region cannot form the double loop-like conformation and, therefore, Seq20 has the lowest binding affinity for SMN-C2. On the contrary, the binding affinity is much higher when the core sequence is contained within a single-stranded RNA loop (Seq21) or DNA bulge (Seq23). The conformationally constrained sequences arising from the complementary base-pairing interactions at the ends of the GA-rich sequences in RNA Seq21 and DNA Seq23 likely stabilize the double loop-like structures, resulting in more favourable ligand binding. These results further support the simulation models where the distance between the 5′ and 3′ ends of the GA-rich sequences is quite short in the bound/folded states (Figures 4D and 5D).

## DISCUSSION

We previously demonstrated that the GA-rich sequence in exon 7 is duplexed with the 3′-end of intron 6 and forms a bulged stem-loop structure (TSL1) *in vitro* and in cells (12). The binding of SMN-C2 only makes subtle changes in the conformation judged by selective 2′-hydroxyl acylation analysed by primer extension (SHAPE) experiments (12). The TSL1 structure is, however, clearly different from the unpaired double loop-like structures uncovered in this report. Although TSL1 may form stably *in vitro* and *in vivo*, this structure must be linearized to ssRNA to be recognizable by some splicing regulatory proteins, such as hnRNP A1 (5) and Tra2β1 (42). During this linearization process, the GA-rich sequence will transiently become single-stranded. The results in this report show unambiguously that SMN-C2 binds to the single-stranded GA-rich sequence, indicating that the single-stranded conformation in this GA-rich region is functionally relevant for both the potency of SMN-C2 and trans-acting regulatory proteins.

Unlike antisense oligonucleotides that bind to specific RNA sequences, small molecules usually recognize RNA secondary or tertiary structures (43–45). These structures can be simple internal bulges that contain 2–6 unpaired nucleotides (e.g. (46)) or complex riboswitches that contain a small-molecule binding cavity, which cannot be discerned from primary sequences (e.g., (47)). In the past few years, RNA–small molecule interaction databases have been built based on RNA structural patterns, including Inforna (48,49) and R-BIND (50). It is difficult, however, to discern likely structures within the short 9-nt primary GA-rich sequence. Nevertheless, the simulation results predicted a plausible and novel aptamer conformation, i.e., a double loop-like structure. GNRA (R = A or G, N = A, U, G or C) is a common tetraloop turning sequence in RNA stem-loops (51). GAAG tetraloop is a naturally occurring variation of the GNRA tetraloop (36,52). In single GAAG tetraloops, a closing base pair (e.g. G–C) is often located at the 5′ and 3′ ends to conformationally constrain and thus stabilize the tetraloop structure (36,51). In the 9-nt GA-rich sequence that consists of two loop-like structures, the compact structure is probably stabilized by the intercalation of compound **1**. As an analogy of GNRA tetraloop in RNA, GNNA and GNAB (B = C, G or T) tetraloops can also stably form in DNA (53), which is consistent with the simulation findings that the double GAAG loop-like structures formed similarly in both DNA and RNA in the presence of compound **1**. Constraining the ends of the GA-rich sequence using complementary base-pairs (Seq21 and Seq23, Scheme 2) strengthened the small-molecule binding, generally supporting the bound/folded simulation states where the distance between the ends of the GA-rich sequence was predicted to be short (Figures 4D and 5D).

The MD simulation results are highly consistent with the NMR and structure-affinity-relationship studies. In the NMR titration experiment, aromatic NMR peaks in compound **1** almost all disappeared when the RNA Seq6 or DNA Seq4 concentration reached 20 mol%, suggesting a fast equilibrium among heterogenous binding states. This is consistent with the free energy surfaces arising from the GaMD simulations, which depict at least three ligand-bound states separated by relatively shallow barriers (Figures 4C and 5C). Compound **1** formed a key π-π stacking interaction between ring D and the 2nd and 4th nucleobases in the 5′ GAAG loop-like structure in DNA Seq6 (3A and 5G in Seq6). Since the dipole moments of aromatic rings are important for π-stacking (54), this binding model was consistent with experimental findings that the ligand-binding was sensitive to the A-to-G or G-to-A substitutions in 3A and 5G (Table 1, Seq11 and 12). The simulated binding mode is also consistent with the relatively strong NMR STD in ring B/C of compound **1**, which, in simulations, formed π-π and lone pair-π stacking interactions with 5G and 3A in DNA Seq6, respectively (Figures 5D and 6B). In addition, simulations demonstrated that the edges of the 3-methyl in the piperazine ring (ring A) and 6′- and 8′-methyl groups in ring D2 were solvent accessible, consistent with the low STD in NMR in both rings (Figures 5D and 6B).

The DNA binding mode is also consistent with the observation that N4-substitution did not hamper the ligand binding to DNA Seq6 (compound 2, Table 2, entry 5). However, in RNA Seq6, simulation revealed that the piperazine ring in the compound interacted with the RNA aptamer via a polar bond between N4 of compound **1** and N3 of the 2G nucleobase (Figure 4D). This is consistent with the finding that the GA-rich RNA was more sensitive to N4 alkylation in the SMN-C2 scaffold than that observed with DNA. A bulky Boc group reduced the binding affinity for RNA Seq6 by 3-fold (Table 1, RNA Seq6 vs Table 2, entry 6). It was also shown in simulation results that the double loop-like ligand-binding pocket was confined by 2G and 5G in DNA Seq6 (Figure 5D, Supplementary Movie S2). Methyl substitution at C4′ of the coumarin (compounds 8–9, Table 2) likely resulted in a steric clash with the 5G nucleobase and therefore the binding affinity was reduced, while a smaller F group at C6′ retained favourable binding (compound 7, Table 2).

All-atom GaMD simulations successfully captured spontaneous binding of the risdiplam analogues to the GA-rich DNA and RNA sequences. However, it is important to note that the GaMD free energy profiles were not fully converged, because still only few ligand binding events (insufficient sampling) were observed in the GaMD simulations. Nevertheless, relatively low-energy conformational states of each system could be identified from the simulations, which uncovered a folded double loop-like conformation induced by small-molecule binding in both DNA and RNA. The induction of the conformational change is consistent with the observation of peak shifts in circular dichroism (CD) spectra of RNA or DNA Seq6 in the presence of compound **1** (Supplementary Figures S20 and S21).

We also discovered that the binding to the GA-rich sequence is not sufficient to correct SMN2 splicing in cellular assays. Several risdiplam analogues retain the ssDNA or ssRNA-binding ability without showing any observable splicing modulation in cells (e.g. compounds 2, 5, 10, 11; Supplementary Table S4, Supplementary Figure S5). This is not unexpected because the GA-rich sequence is not the primary target for splicing modulation (15).

It is important to note that the dissociation constants (*K*_d_) for the binding of the small molecules to either the GA-rich sequence in this study or to the 5′ splice site–U1 snRNP complex in the previous report (15) are in micromolar range, orders of magnitude higher than the EC_50_ values of some of the active splicing modifiers in cell-based splicing assay (e.g. Table 2, entries 1–4). The discrepancy between the high cellular activity and relatively low binding affinity is actually quite common in this type of action-dependent drugs. Compared to the traditional occupation-dependent drugs (e.g. kinase inhibitors), risdiplam analogues are not required to remain bound to the mRNA once exon 7 splicing is complete. Therefore, the effective dose of risdiplam analogues in cells can be much smaller than the *K*_d_. In addition, the small-molecule binding to the GA-rich sequence in SMN2 exon 7 was demonstrated to induce the recruitment or displacement of splicing-regulatory proteins in the pre-mRNA (12,13). The involvement of these RNA-binding proteins may also enhance the cellular activity and selectivity of the small-molecule splicing modifiers (55).

Our studies demonstrated that SMN-C2, in general, binds with higher affinity to ssDNA sequences relative to ssRNA (Table 1). In cells, most of the DNA is doubly stranded in the genome. However, ssDNAs transiently form during DNA replication. Therefore, there is a concern that the DNA-binding ability may associate with genotoxicity (41). Although one of the strongest GA-rich sequence ligands, SMN-C5, is negative in the Ames test (41), further studies are required to correlate genotoxicity and DNA-binding.

In the presence of a stable TSL2 in SMN2 exon 7, the two binding sites of risdiplam analogues on SMN2 pre-mRNA exon 7, while not close together in sequence, are probably close together in space (Figure 1). Although the GA-rich sequence is crucial in maintaining the drugs’ potency for regulating splicing, binding to the GA-rich alone is not sufficient to induce SMN2 exon 7 inclusion. It is, therefore, possible that the GA-rich sequence serves as an auxiliary binding site and facilitates ligand binding to the 5′ splice site, i.e., a small-molecule delivery relay.

As previously hypothesized (13), binding to both the GA-rich sequence and to the 5′ splice site contributes to the selectivity for the risdiplam analogues. Compared to another structurally unrelated splicing modulator for SMN2 exon 7, branaplam, which only acts through binding to the 5′ splice site of the exon (56) without detectable binding to the GA-rich sequence, risdiplam analogue SMN-C3 only significantly affects splicing in 13 genes, while branaplam affects the splicing of 36 genes (13). The forkhead box M1 (FoxM1) gene is one of the 13 risdiplam-sensitive genes but lacks a GA-rich sequence (16). Compared to FoxM1, the SMN2 gene is ∼10 times more sensitive to a risdiplam analogue, RG-7800 (8), consistent with the hypothesis that the GA-rich sequence enhances the drug potency. Recently, it was discovered that another risdiplam analogue, TEC-1, with a modified ring B/C (57), has even fewer off-target effects. By changing the risdiplam's pyridopyrimidinone core in ring B/C into a quinazolinone, the FoxM1 gene splicing becomes even less sensitive to TEC-1 than risdiplam. This result underscored the possibility that sequence recognition of the risdiplam analogue can be changed by modification of the ring B/C.

## CONCLUSION

Our results reveal a new type of small molecule–RNA recognition mechanism that is relevant to the mechanism of action of a recently approved drug, risdiplam. Through molecular dynamic simulations, we revealed a new drug-inducible GAAG double loop-like structure for both DNAs and RNAs, which can be simply represented by a consecutive primary sequence of 9 nts, i.e., GAAGGAAGG. In the literature, long RNA sequence recognition by the small molecules is often associated with G-quadruplex formation (e.g. (GGGGCC) repeats (58)). To our knowledge, this double loop-like structure is the first example of a consecutive RNA sequence of 9 or more nts that a small molecule can selectively recognize in the absence of G-quadruplexes (50).

## DATA AVAILABILITY

All software packages used in this report are available in public repositories, including AMBER18 (http://ambermd.org/), CPPTRAJ (https://amber-md.github.io/cpptraj/CPPTRAJ.xhtml), and PyReweighting (https://github.com/MiaoLab20/PyReweighting). Representative bound conformations generated by GaMD simulations are available in PDB files in Model Archive repository (https://modelarchive.org): RNA–compound 1 complex, Bound/Folded state (https://modelarchive.org/doi/10.5452/ma-fxj31), Bound/Intermediate state (https://modelarchive.org/doi/10.5452/ma-l2fj8); DNA–compound 1 complex, Bound/Folded state (https://modelarchive.org/doi/10.5452/ma-aghox), Bound/Intermediate state (https://modelarchive.org/doi/10.5452/ma-ut0vm), Bound/Unfolded state (https://modelarchive.org/doi/10.5452/ma-royyf).

## Supplementary Material

gkab602_Supplemental_Files
