# Supplementary material for: Recognition of single-stranded nucleic acids by small-molecule splicing modulators
Source: Nucleic Acids Res. 2021 Jul 20;49(14):7870–83. doi: 10.1093/nar/gkab602 (PMC8373063; doi:10.1093/nar/gkab602)
Supplement: gkab602_Supplemental_Files [file gkab602_supplemental_files.zip › NAR_SI_Rev_2021-06-09.pdf]

Supporting Information For

“ Recognition of single-stranded nucleic acids by small-molecule splicing modulators”

Table of Contents

1. Minigene SMN2 Splicing Assays in 293T cells 2
2. Isothermal Titration Calorimetry (ITC) Assay 4
3. Size Exclusion Chromatography 4
4. Surface Plasmon Resonance (SPR) Assay 4
5. Molecular Dynamic Simulation 5
6. Chemistry 6
7. Tables and Figures 11
8. ^{1}H and ^{13}C NMR Spectra and HPLC 26

Methods

1. Minigene SMN2 Splicing Assays in 293T cells

The Gaussia luciferase minigenes for SMN2 exon 7 skipping were transfected into 293T cells following the Lipofectamine 2000 protocol (Thermo) in a 6-well plate. After 6 h of incubation, cells were disassociated with 0.5 mL TrypLE (Gibco, # 12605036) for 5 min. The trypsinization was stopped by adding 1.5 mL of full growth medium (DMEM + 10% FBS) to the wells. The cell number was counted using Countess II Automated Cell Counter (Thermo, # AMQAX1000) and were diluted in low serum medium (DMEM + 3% FBS). For PAGE, the cells were seeded into a 24-well plate (0.2 million per well) in 0.35 mL medium and incubate for 2 h at 37 °C. The cells were then treated with SMN-C2 or nusinersen at various concentrations. In the wells containing nusinersen, 1 μL Endo-Porter delivery reagent (GeneTools, Philomath, OR, USA; ordered through Fisher Scientific, # NC1501848) was added and mixed by gently swirling the plate. The cells were incubated for another 24 h at 37 °C before being harvested by aspirating the medium and adding 300 μL RLT buffer (RNeasy mini kit) in each well. The total RNA was extracted using the RNeasy mini kit (Qiagen, Hilden, Germany #74104) according to the manufacturer's manual. The RNA was reversely transcribed using M-MLV reverse transcriptase (Promega, # M1701) and a poly(dT) primer. The spliced products were amplified by PCR using the primer set, pCI-FW: 5' GGCTAGAGTACTTAATACGACTCAC, and GLuc-RV: 5'-CAGCGATGCAGATCAGG-GC. The PAGE is performed with 8 % TBE gels (180 V, 30 min). The gels were stained with 0.003 % SYBR Safe DNA Gel Stain (Thermo, # S33102) in 0.5× TBE buffer for 10 min (for full gel images for Figure 3, see Figure S15).

For luciferase readout, the transfected cells were transferred into a 384-well plate (Greiner #784075) at 15,000 cells per well in 27 μL medium. The compounds were 1:2 serial diluted for 10 concentration points starting at 1 μM (final concentration) and added into the wells in triplicates (3 μL). The plate was incubated for 48 h at 37 °C with 5% CO_2 after the addition of the compounds. The Gaussia luciferase reading agent was prepared by diluting coelenterazine Gaussia luciferase substrate (Thermo #1862575) at 1:500 ratio into the Gaussia luciferase buffer containing 50 mM Tris-Cl pH 7.5, 10 mM $MgCl_2$, 1 mM DTT, 1 mM ATP, and 0.2% BSA in M-PER Mammalian Protein Extraction Reagent (Thermo #78501). 15 μL of the Gaussia luciferase reading reagent was added to each well and incubated for 5 min at room temperature followed by luminescence measurements (Cytation 5, BioTek). A non-linear regression dose response curve (four parameters) was used for curve-fitting and the EC_{50} values were determined using GraphPad Prism 8.

The minigene reporter plasmid was constructed by inserting the below sequence between T7 promoter and SV40 poly(A) sequences in pCI vector:

CTAGCCTCGAGATGGCTTTGGGAAGTATGTTAATTTCATGGTACATGAGTGGCTAT
CATACTGGCTATTATATGGTAAGTAATCACTCAGCATCTTTTCCTGACAATTTTTTT

GTAGTTATGTGACTTTGTTTTGTAAATTTATAAAATACTACTTGCTTCTCTCTTTATAT
TACTAAAAAATAAAAATAAAAAAATACAACTGTCTGAGGCTTAAATTACTCTTGCAT
TGTCCCTAAGTATAATTTTAGTTAATTTTAAAAAGCTTTCATGCTATTGTTAGATTATT
TTGATTATACACTTTTGAATTGAAATTATACTTTTTCTAAATAATGTTTTAATCTCTGA
TTTGAAATTGATTGTAGGGAATGGAAAAGATGGGATAATTTTTCATAAATGAAAAAT
GAAATTCTTTTTTTTTTTTTTTTTTTTGAGACGGAGTCTTGCTCTGTTGCCCAGGC
TGGAGTGCAATGGCGTGATCTTGGCTCACAGCAAGCTCTGCCTCCTGGATTCAC
GCCATTCTCCTGCCTCAACCTCCCAAGTAGCTGGGATTAGAGGTCCCCACCACC
ATGCCTGGCTAATTTTTTGTACTTTCAGTAGAAACGGGGTTTTGCCATGTTGGCC
AGGCTGTTCTCGAACTCCTGAGCTCAGGTGATCCAACTGTCTCGGCCTCCCAAA
GTGCTGGGATTACAGGCGTGAGCCACTGTGCCTAGCATGAGCCACCACGCCGG
CCTAATTTTTAAATTTTTTGTAGAGACAGGGTCTCATTATGTTGCCCAGGGTGGTG
TCAAGCTCCAGGTCTCAAGTGATCCCCCTACCTCCGCCTCCCAAAGTTGTGGGA
TTGTAGGCATGAGCCACTGCAAGAAAACCTTAACTGCAGCCTAATAATTGTTTTCT
TTGGGATAACTTTTAAAGTACATTAAAAGACTATCAACTTAATTTCTGATCATATTTT
GTTGAATAAAATAAGTAAAATGTCTTGTGAAACAAAATGCTTTTTAACATCCATATA
AAGCTATCTATATATAGCTATCTATATCTATATAGCTATTTTTTTTAACTTCCTTTATTT
TCCTTACAGGGTTTCAGACAAAATCAAAAAGAAGGAAGGTGCTCACATTCCTTAA
ATCTAAGGAGTAAGTCTGCCAGCATTATGAAAGTGAATCTTACTTTTGTAAAACTT
TATGGTTTGTGGAAAACAAATGTTTTTGAACATTTAAAAAGTTCAGATGTTAGAAA
GTTGAAAGGTTAATGTAAAACAATCAATATTAAAGAATTTTGATGCCAAAACTATTA
GATAAAAGGTTAATCTACATCCCTACTAGAATTCTCATACTTAACTGGTTGGTTGTG
TGGAAGAAACATACTTTCACAATAAAGAGCTTTAGGATATGATGCCATTTTATATCA
CTAGTAGGCAGACCAGCAGACTTTTTTTTATTGTGATATGGGATAACCTAGGCATA
CTGCACTGTACACTCTGACATATGAAGTGCTCTAGTCAAGTTTAACTGGTGTCCA
CAGAGGACATGGTTTAACTGGAATTCGTCAAGCCTCTGGTTCTAATTTCTCATTTG
CAGGAAATGCTGGCAAGAGCAGCACTAAAGGAGTCAAAGTTCTGTTTGCCCTGA
TCTGCATCGCTGTGGCCGAGGCCAAGCCCACCGAGAACAACGAAGACTTCAAC
ATCGTGGCCGTGGCCAGCAACTTCGCGACCACGGATCTCGATGCTGACCGCGG
GAAGTTGCCCGGCAAGAAGCTGCCGCTGGAGGTGCTCAAAGAGATGGAAGCCA
ATGCCCGGAAAGCTGGCTGCACCAGGGGCTGTCTGATCTGCCTGTCCCACATC
AAGTGCACGCCCAAGATGAAGAAGTTCATCCCAGGACGCTGCCACACCTACGAA
GGCGACAAAGAGTCCGCACAGGGCGGCATAGGCGAGGCGATCGTCGACATTCC
TGAGATTCCTGGGTTCAAGGACTTGGAGCCCATGGAGCAGTTCATCGCACAGGT
CGATCTGTGTGTGGACTGCACAACTGGCTGCCTCAAAGGGCTTGCCAACGTGC
AGTGTTCTGACCTGCTCAAGAAGTGGCTGCCGCAACGCTGTGCGACCTTTGCC
AGCAAGATCCAGGGCCAGGTGGACAAGATCAAGGGGGCCGGTGGTGACTAACC
CGGGCGGCCGCTTCGAGCAGACATGA

Single-point mutations in the plasmid were made in exon 7 (underlined) using Phusion Site-Directed Mutagenesis Kit (Thermo, # F541) with the following target sequences:

WT: AAAAGAAGGAAGGTGCTC

M1: AAAATAAGGAAGGTGCTC
M2: AAAAGGAGGAAGGTGCTC
M3: AAAAAAAGGAAGGTGCTC
M4: AAAAGAAGAAAGGTGCTC

2. Isothermal Titration Calorimetry (ITC) Assay

Isothermal calorimetric titrations were carried out on a Malvern Analytical MicroCal PEAQ-ITC at 25 °C. The DNA (see above for the calibration procedure) and the SMN-C2 water solutions containing the desired amount of the materials were freeze-dried overnight (Labconco FreeZone 4.5 Liter Benchtop Freeze Dry System) and then re-suspended in appropriate volume of buffer containing 5 % DMSO, 100 mM NaCl and 30 mM MES buffer at pH 6.0 (e.g., 350 µL for DNA Seq6, 80 µL for SMN-C2). Each ITC titration had an initial injection of 0.4 µL followed by 18 injections of SMN-C2 (e.g., 250 µM for DNA Seq6 titration) of 2 µL for 4 s at 150 s intervals into the DNA (e.g. Seq6 at 25 µM) in sample cell. The analysis was performed using the instrument's MicroCal PEAQ-ITC Analysis Software (Malvern Analytical). The ITC data was fit using the One Set of Sites mode to calculate the dissociate constant (K_d), binding stoichiometry, and the changes in enthalpy and Gibbs free energy. In differential power (DP) trace, the baseline was subtracted for better visualization, which has no impact on calculations.

3. Size Exclusion Chromatography

The size exclusion chromatography was performed on a ÄKTA pure protein purification system (Cytiva, Thousand Oaks, CA, USA) equipped with a Superdex 75 Increase 10/300 GL column (Cytiva #29148721). The samples in 0.1 mL PBS buffer were injected and eluted with PBS buffer (pH. 7.4, 0.5 mL/min, 25 mL). The absorption at 280 nm was monitored in real-time and the chromatography data (A280 over the retention volume) were generated and exported using the instrument analytical software, UNICORN, and replotted with Prism 8. For the annealed mixture of SMN-C2 and DNAs, the eluent was constantly collected in 0.5 mL fractions. The SMN-C2 content in each fraction was subsequently measured using a fluorescence microplate reader (Cytation 5, BioTek, Ex/Em = 410/480 nm).

4. Surface Plasmon Resonance (SPR) Assay

SPR experiments were performed on a Biacore T200 (GE Healthcare) instrument at 25 °C using streptavidin pre-coated SA sensor chips (GE Healthcare). The running buffer composed of 10 mM HEPES, 100 mM NaCl, 0.05% Tween 20 (w/v), 5 mM EDTA, 0.1% (v/v) DMSO at pH 6.8 was prepared freshly, filtered through the 0.22 µm PVDF membrane prior to use. 5'-biotinylated RNA Seq4 was purchased from GenScript and dissolved in nuclease-free water to a concentration of 100 µM. For immobilization of the biotinylated RNA, the sensor chip was firstly conditioned with 3 consecutive 1 min injections of high salt solution (50 mM NaOH, 1M NaCl) at a flow rate of 10 µL/min. Next, the biotinylated RNA was diluted 1000× in running buffer (100 nM) and applied over the streptavidin sensor chip surface at a flow rate of 10 µL/min to achieve immobilization level of about 800 RU. Finally, alkyne-PEG-biotin (50 µM in running

buffer) was injected (1 min, 10 μL/min) to block remaining streptavidin surface binding sites. The kinetics analysis was performed following the BiaControl Software Wizard Kinetics protocol. The small molecules (HCl salt form, 10 mM in water) were diluted in the running buffer to six concentrations (0, 0.1, 1, 5, 10, 20 μM for SMN-C2; 0, 1, 5, 10, 20, 40 μM for SMN-C5; 0, 1, 10, 20, 40, 80 μM for SMN-C3) and titrated over the immobilized RNA Seq4 (contact time: 1 min, flow rate: 30 μL/min). The data analysis was performed using the instrument BiaEvaluation Software. All monitored resonance signals were subtracted with signals from a non-binding reference channel. Kinetic values (K_d, k_a, k_d) were calculated using the BiaEvaluation Software Binding Affinity protocol with 1:1 fitting. Figures were plotted using TraceDrawer 1.9.1 (Generic Biophysics Version).

5. Molecular Dynamic Simulation

Gaussian Accelerated Molecular Dynamics (GaMD)

GaMD is an enhanced sampling approach wherein a harmonic boost potential is added to smooth the potential energy surface and reduce energy barriers(1). GaMD provides efficient unconstrained enhanced sampling without the need for predefined collective variables. A brief summary of the method is described here.

Consider a system with *N* atoms at positions $r \equiv \{\vec{r}_1, \cdots, \vec{r}_N\}$. When the system potential $V(r)$ is lower than a reference energy *E*, the modified potential $V^*(r)$ of the system is calculated as:

$$V^*(\vec{r}) = V(\vec{r}) + \Delta V(\vec{r}), \quad (1)$$

$$\Delta V(\vec{r}) = \frac{1}{2}k(E - V(\vec{r}))^2, V(\vec{r}) < E, \quad (2)$$

where *k* is the harmonic force constant. The parameters *E* and *k* can be determined by applying three principles of enhanced sampling. The reference energy should fall in range as follows:

$$V_{max} \leq E \leq V_{min} + \frac{1}{k}, \quad (3)$$

Where V_{max} and V_{min} are the system maximum and minimum potential energies. To ensure that Eq. (3) is valid, *k* has to satisfy: $k \leq \frac{1}{V_{max} - V_{min}}$. Let us define $\leq \frac{1}{V_{max} - V_{min}}$, then $0 < k_0 \leq 1$. The standard deviation of ΔV needs to be small enough (i.e., narrow distribution) to ensure precise reweighting using cumulant expansion to the second order: $\sigma_{\Delta V} = k(E - V_{avg})\sigma_V \leq \sigma_0$, where V_{avg} and σ_V are the average and standard deviation of ΔV with as a user-specified upper limit (e.g. $10k_BT$) for accurate reweighting. When *E* is set to the lower bound $E = V_{max}$, k_0 can be calculated as:

$$k_0 = \min(1.0, k_0') = min\left(1.0, \frac{\sigma_0}{\sigma_V} . \frac{V_{max} - V_{min}}{V_{max} - V_{avg}}\right) \quad (4)$$

Alternatively, when the threshold energy *E* is set to its upper bound $E = V_{min} + \frac{1}{k}$, k_0 is set to:

$$k_0 = k_0'' \equiv \left(1 - \frac{\sigma_0}{\sigma_V}\right) . \frac{V_{max} - V_{min}}{V_{avg} - V_{min}} \quad (5)$$

If k_0'' is calculated between 0 and 1. Otherwise, k_0'' is calculated using Eq. (4).

The original GaMD method provides schemes to add only the total potential boost ΔV_P, only dihedral potential boost ΔV_D, or the dual potential boost (both ΔV_P and ΔV_D). Dual-boost GaMD provides higher acceleration than the other two types of simulations. The simulation parameters comprise of the threshold energy *E* for applying boost potential and effective harmonic force constants, k_{0P} and k_{0D} and for total and dihedral potential boost.

Energetic Reweighting of GaMD Simulations

The GaMD simulations can be reweighted to calculate the original potential mean force (PMF) free energy profiles. The probability distribution along a reaction coordinate is written as $p^*(A)$. Given the boost potential $\Delta V(r)$ of each frame, $p^*(A)$ can be reweighted to recover the canonical ensemble distribution $p(A)$, as:

$$p(A_j) = p^*(A_j) \frac{\langle e^{\beta \Delta V(r)} \rangle_j}{\sum_{i=1}^{M} \langle p^*(A_i) e^{\beta \Delta V(r)} \rangle_i}, \quad j = 1,\ldots, M, \quad (6)$$

where *M* is the number of bins, $\beta = k_B T$ and $\langle e^{\beta \Delta V(r)} \rangle_j$ is the ensemble-averaged Boltzmann factor of $\Delta V(r)$ for simulation frames found in the jth bin. The ensemble-averaged reweighting factor can be approximated using cumulant expansion:

$$\langle e^{\beta \Delta V(r)} \rangle_j = \exp\left\{ \sum_{k=1}^{\infty} \frac{\beta^k}{k!} C_k \right\}, \quad (7)$$

where first two cumulants are given by

$$C_1 = \langle \Delta V \rangle,$$
$$C_2 = \langle \Delta V^2 \rangle - \langle \Delta V \rangle^2 = \sigma_V^2. \quad (8)$$

The boost potential derived from GaMD simulations usually follows near-Gaussian distribution. Cumulant expansion to the second order thus provides a good approximation for computing the reweighting factor(1, 2). The reweighted free energy $F(A) = -k_B T \ln p(A)$ is calculated as:

$$F(A) = F^*(A) - \sum_{k=1}^{2} \frac{\beta^k}{k!} C_k + F_c, \quad (9)$$

where $F^*(A) = -k_B T \ln p^*(A)$ is the modified free energy obtained from GaMD simulation and F_c is a constant.

6. Chemistry

Reagents and solvents were purchased from commercial sources (Fisher, Sigma-Aldrich and Combi-Blocks) and used as received. Reactions were tracked by TLC (Silica gel 60 F_{254}, Merck) and Waters ACQUITY UPLC-MS system (ACQUITY UPLC H Class Plus in tandem with Qda Mass Detector). Intermediates and products were purified by a Teledyne ISCO Combi-Flash system using prepacked SiO_2 cartridges.

NMR spectra were acquired on a Bruker AV400 instrument (400 MHz for ^{1}H NMR, 100 MHz for ^{13}C NMR) or Bruker AV500 instrument (500 MHz for ^{1}H NMR, 125 MHz for ^{13}C NMR). Data were recorded as follows: chemical shift (δ) in ppm, coupling constant (J) in Hz, multiplicity (s = singlet, d = doublet, t = triplet, m = multiplet or overlap of nonequivalent resonances, brs = broad singlet). ^{13}C shifts were obtained with ^{1}H decoupling. MS-ESI spectra were recorded on Waters Qda Mass Detector. HPLC was performed on Waters ACQUITY UPLC H Class Plus system using Waters BEH C18 (2.1 mm × 50 mm, 1.7 μm) column and peak detection at 254nm with UV.

SMN-C2, **SMN-C3**, **SMN-C5**, compound **1** were synthesized following procedures reported in literature(3).

*(S)-tert-butyl(2-(4-(3-(6,8-dimethylimidazo[1,2-a]pyrazin-2-yl)-2-oxo-2H-chromen-7-yl)-2-methylpiperazin-1-yl)ethyl)carbamate. (**2**)*

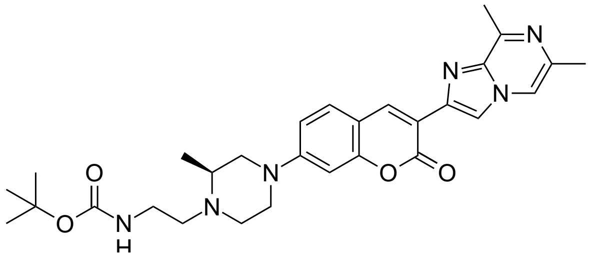

To a solution of compound **1** (10 mg, 0.025 mmol) and tert-butyl (2-bromoethyl) carbamate (11 mg, 0.05 mmol) in acetonitrile was added Cs_2CO_3 (24 mg, 0.075 mmol), the reaction mixture was heated to 70 °C and stirred overnight. TLC and LC-MS showed completion of reaction. The reaction mixture was cooled to room temperature, filtered and concentrated under vacuum. The residue was purified by column chromatography (0 - 5% CH_3OH in CH_2Cl_2) to afford 6 mg yellow solid. (yield,43.9%). ^{1}H NMR (400 MHz, $CDCl_3$): δ 8.74 (s, 1H), 8.45 (s, 1H), 7.77 (s, 1H), 7.51 (d, J = 8.8 Hz, 1H), 6.86 (dd, J = 8.8, 2.4 Hz, 1H), 6.75 (d, J = 2.4 Hz, 1H), 4.96 (brs, 1H), 3.59 (t, J = 12 Hz, 2H), 3.31-3.20 (m, 3H), 3.03-2.92 (m, 6H), 2.67 (brs, 1H), 2.49-2.33 (m, 5H), 1.48 (s, 9H), 1.17 (d, J = 6.4 Hz, 3H); ^{13}C NMR (100 MHz, $CDCl_3$): δ 160.7, 156.0, 155.5, 153.4, 151.2, 140.1, 139.3, 138.9, 137.1, 129.3, 115.4, 114.4, 113.6, 111.9, 111.1, 100.3, 79.3, 54.8, 54.3, 52.4, 49.9, 47.5, 28.5, 20.8, 20.5, 15.8. MS-ESI (m/z) $[M + H]^+$ 533.29.

General Procedures for compounds **3** to **9**

Step 1: Synthesis of Coumarin intermediate

A mixture of substituted phenyl acetic acid (1 eq), substituted 2-hydroxylbenzaldehyde (1 eq), triethylamine and acetic anhydride (1 : 5) was heated to 100 °C for 1 h in a Biotage Initiator$^+$ microwave reactor. TLC and LC-MS showed completion of reaction. The mixture was cooled to room temperature, poured into 10 mL ice water and extracted with ethyl acetate. Organic layer was washed with brine, dried with anhydrous sodium sulphate and removed under vacuum. Acetonitrile was added to the residue and the resulted precipitate was filtered and washed with acetonitrile to afford a white or yellow solid which was used without further purification.

Step 2: Synthesis of final product

To a solution of Coumarin intermediate (1 eq) and 1-methylpiperazine (2 eq) in DMSO was added K_2CO_3 (3 eq). The reaction mixture was heated to 120 °C and stirred for 2 h. TLC and LC-MS showed completion of reaction. The mixture was cooled to room temperature, poured into ice water and extracted with ethyl acetate. Organic layer was washed with brine, dried with anhydrous sodium sulphate and removed under vacuum. The residue was purified by column chromatography (0 - 5% CH_3OH in CH_2Cl_2) to afford product as yellow solid.

*3-(2-chlorophenyl)-7-(4-methylpiperazin-1-yl)-2H-chromen-2-one (**3**)*

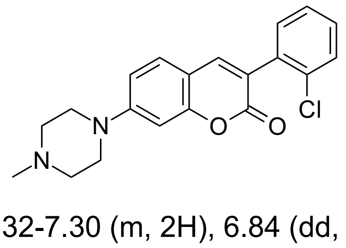

Following General Procedures, from 2-(2-chlorophenyl)acetic acid (85 mg, 0.5 mmol) and 4-fluoro-2-hydroxybenzaldehyde (70 mg, 0.5 mmol), 10 mg of compound **3** was obtained as yellow solid (two steps yield, 9.6%). ^1H NMR (400 MHz, $CDCl_3$): δ 7.62 (s, 1H), 7.48-7.46 (m, 1H), 7.42-7.40 (m, 1H), 7.35 (d, J = 8.8 Hz, 1H), 7.32-7.30 (m, 2H), 6.84 (dd, J = 8.8, 2.4 Hz, 1H), 6.77 (d, J = 2.4 Hz, 1H), 3.40 (t, J = 5.2 Hz, 4H), 2.60 (t, J = 5.2 Hz, 4H), 2.38 (s, 3H); ^{13}C NMR (100 MHz, $CDCl_3$): δ 160.7, 156.2, 153.8, 142.9, 134.6, 134.0, 131.8, 130.0, 129.7, 128.9, 126.9, 122.0, 111.9, 110.9, 101.1, 54.7, 47.6, 46.2. MS-ESI (m/z) $[M + H]^+$ 355.13.

*3-(3-chlorophenyl)-7-(4-methylpiperazin-1-yl)-2H-chromen-2-one (**4**)*

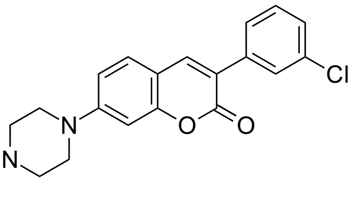

Following General Procedures, from 2-(3-chlorophenyl)acetic acid (85 mg, 0.5 mmol) and 4-fluoro-2-hydroxybenzaldehyde (70 mg, 0.5 mmol), 13 mg of compound **4** was obtained as yellow solid (two steps yield, 12.5%). ^1H NMR (500 MHz, $CDCl_3$): δ 7.72 (s, 1H), 7.69 (t, J = 2.0 Hz, 1H), 7.60 (dt, J = 7.0, 2.0 Hz, 1H), 7.38-7.33 (m, 3H), 6.84 (dd, J = 9.0, 2.5 Hz, 1H), 6.75 (d, J = 2.5 Hz, 1H), 3.40 (t, J = 5.0 Hz, 4H), 2.60 (brs, 4H), 2.39 (s, 3H); ^{13}C NMR (125 MHz, $CDCl_3$): δ 161.1, 155.9, 153.7, 140.8, 137.3, 134.4, 129.7, 129.0, 128.4, 128.3, 126.7, 121.8, 112.0, 111.3, 100.8, 54.7, 47.5, 46.1. MS-ESI (m/z) $[M + H]^+$ 355.15.

*3-(4-chlorophenyl)-7-(4-methylpiperazin-1-yl)-2H-chromen-2-one (**5**)*

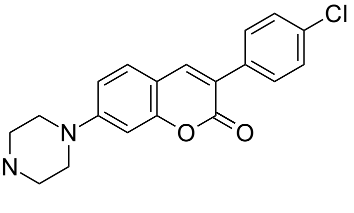

Following General Procedures, from 2-(4-chlorophenyl)acetic acid (85 mg, 0.5 mmol) and 4-fluoro-2-hydroxybenzaldehyde (70 mg, 0.5 mmol), 11 mg of compound **5** was obtained as yellow solid (two steps yield, 10.5%). ^1H NMR (500 MHz, $CDCl_3$): δ 7.71 (s, 1H), 7.65 (dt, J = 9.0, 2.5 Hz, 2H), 7.40-7.37 (m, 3H), 6.84 (dd, J = 8.5, 2.5 Hz, 1H), 6.75 (d, J = 2.5 Hz, 1H), 3.43 (brs, 4H), 2.65 (brs, 4H), 2.42 (s, 3H); ^{13}C NMR (125

MHz, $CDCl_3$): δ 161.2, 155.8, 153.5, 140.2, 134.2, 134.0, 129.8, 128.9, 128.7, 122.2, 112.0, 111.4, 100.9, 54.6, 47.4, 46.0. MS-ESI (m/z) $[M + H]^+$ 355.14.

*3-(3-chlorophenyl)-5-fluoro-7-(4-methylpiperazin-1-yl)-2H-chromen-2-one (**6**)*

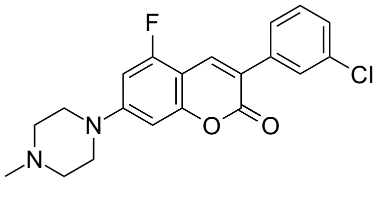

Following General Procedures, from 2-(3-chlorophenyl)acetic acid (51 mg, 0.3 mmol) and 2,4-difluoro-6-hydroxybenzaldehyde (50 mg, 0.3 mmol), 9 mg of compound **6** was obtained as yellow solid (two steps yield, 13.7%). ^1H NMR (500 MHz, $CDCl_3$): δ 7.90 (s, 1H), 7.69 (t, *J* = 2.0 Hz, 1H), 7.59 (dt, *J* = 6.5, 2.0 Hz, 1H), 7.37-7.34 (m, 2H), 6.54-6.51 (m, 2H), 3.37 (t, *J* = 5.0 Hz, 4H), 2.56 (t, *J* = 5.0 Hz, 4H), 2.37 (s, 3H); ^{13}C NMR (125 MHz, $CDCl_3$): δ 161.0, 160.5, 159.0, 156.0, 153.7, 137.0, 134.5, 133.9, 129.8, 128.5, 126.7, 121.5, 101.3, 97.5, 96.4, 54.6, 47.4, 46.2. MS-ESI (m/z) $[M + H]^+$ 373.15.

*3-(4-chlorophenyl)-5-fluoro-7-(4-methylpiperazin-1-yl)-2H-chromen-2-one (**7**)*

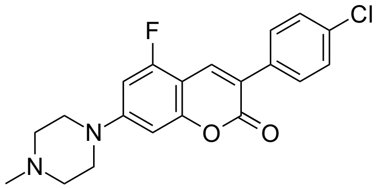

Following General Procedures, from 2-(4-chlorophenyl)acetic acid (51 mg, 0.3 mmol) and 2,4-difluoro-6-hydroxybenzaldehyde (50 mg, 0.3 mmol), 10 mg of compound **7** was obtained as yellow solid (two steps yield, 15.2%). ^1H NMR (500 MHz, $CDCl_3$): δ 7.87 (s, 1H), 7.64 (dt, *J* = 9.0, 2.5 Hz, 2H), 7.39 (dt, *J* = 9.0, 2.5 Hz, 2H), 6.53-6.50 (m, 1H), 3.37 (t, *J* = 5.0 Hz, 4H), 2.56 (t, *J* = 5.0 Hz, 4H), 2.37 (s, 3H); ^{13}C NMR (125 MHz, $CDCl_3$): δ 160.9, 160.7, 159.0, 155.9, 153.6, 134.4, 133.7, 133.3, 129.8, 128.7, 121.7, 101.4, 97.5, 96.4, 54.6, 47.4, 46.2. MS-ESI (m/z) $[M + H]^+$ 373.13.

*3-(3-chlorophenyl)-4-methyl-7-(4-methylpiperazin-1-yl)-2H-chromen-2-one (**8**)*

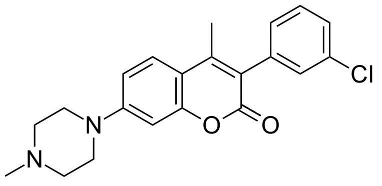

Following General Procedures, from 2-(3-chlorophenyl)acetic acid (85 mg, 1 mmol) and 1-(4-fluoro-2-hydroxyphenyl)ethanone (77 mg, 1 mmol), 12 mg of compound **8** was obtained as light yellow solid (two steps yield, 11.1%). ^1H NMR (500 MHz, $CDCl_3$): δ 7.51 (d, *J* = 9.0 Hz, 1H), 7.39-7.34 (m, 2H), 7.30 (t, *J* = 2.0 Hz, 1H), 7.19 (dt, *J* = 6.5, 2.0 Hz, 1H), 6.86 (dd, *J* = 9.0, 2.5 Hz, 1H), 6.76 (d, *J* = 2.5 Hz, 1H), 3.41 (t, *J* = 5.0 Hz, 4H), 2.64 (brs, 4H), 2.41 (s, 3H), 2.25 (s, 3H); ^{13}C NMR (125 MHz, $CDCl_3$): δ 161.5, 154.7, 153.5, 148.6, 136.9, 134.3, 130.6, 129.7, 128.8, 128.2, 126.1, 121.9, 112.0, 111.7, 101.3, 54.7, 47.5, 46.1, 16.6. MS-ESI (m/z) $[M + H]^+$ 369.17.

*3-(4-chlorophenyl)-4-methyl-7-(4-methylpiperazin-1-yl)-2H-chromen-2-one (**9**)*

Following General Procedures, from 2-(4-chlorophenyl)acetic acid (85 mg, 0.5 mmol) and 1-(4-fluoro-2-hydroxyphenyl)ethanone (77 mg, 0.5 mmol), 11 mg of compound **9** was obtained as light yellow solid (two steps yield, 10.1%). ^{1}H NMR (500 MHz, $CDCl_3$): δ 7.50 (d, J = 8.5 Hz, 1H), 7.41 (dt, J = 8.5, 2.0 Hz, 2H), 7.24 (dt, J = 8.5, 2.0 Hz, 2H), 6.86 (dd, J = 9.0, 2.5 Hz, 1H), 6.76 (d, J = 2.0 Hz, 1H), 3.39 (t, J = 5.0 Hz, 4H), 2.61 (brs, 4H), 2.39 (s, 3H), 2.25 (s, 3H); ^{13}C NMR (125 MHz, $CDCl_3$): δ 161.6, 154.7, 153.4, 148.4, 134.0, 133.5, 132.0, 128.7, 126.1, 122.0, 112.1, 111.7, 101.3, 54.7, 47.6, 46.1, 16.5. MS-ESI (m/z) $[M + H]^+$ 369.15.

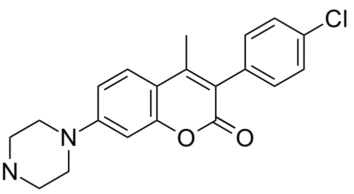

*3-(imidazo[1,2-a]pyridin-2-yl)-7-(piperazin-1-yl)-2H-chromen-2-one. (**10**)*

A mixture of 3-(2-bromoacetyl)-7-fluoro-2*H*-chromen-2-one (50 mg, 0.17 mmol) and 2-aminopyridine (16 mg, 0.17 mmol) in acetonitrile was heated to 120 °C for 20 min in a Biotage Initiator$^+$ microwave reactor. TLC and LC-MS showed completion of reaction. The reaction mixture was filtered and the filter cake was washed with acetonitrile to afford a yellow solid which was used in next step without further purification.

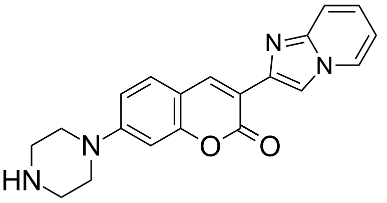

To a solution of the above intermediate (40 mg, 0.14 mmol) and piperazine (24 mg, 0.28 mmol) in DMSO was added K_2CO_3 (58 mg, 0.42mmol). The reaction mixture was heated to 120 °C and stirred for 2 h. The mixture was cooled to room temperature and then poured into ice water. The precipitation was filtered, washed and dried. The residue was purified by column chromatography (0 - 10% CH_3OH in CH_2Cl_2) to afford 32 mg of compound **10** as yellow solid (two steps yield, 52.7%). ^{1}H NMR (500 MHz, DMSO-$_{d6}$): δ 8.72 (s, 1H), 8.62 (d, J = 7.0 Hz, 1H), 8.52 (s, 1H), 7.69 (d, J = 9.0 Hz, 1H), 7.54 (d, J = 9.0 Hz, 1H), 7.29-7.25 (m, 1H), 7.01 (dd, J = 9.0, 2.5 Hz, 1H), 6.90-6.85 (m, 2H), 3.29 (t, J = 5 Hz, 4H), 2.82 (t, J = 5 Hz, 4H); ^{13}C NMR (125 MHz, DMSO-$_{d6}$): δ 159.7, 155.0, 153.8, 144.2, 138.6, 138.5, 129.6, 127.3, 125.6, 116.1, 114.6, 112.2, 111.7, 110.2, 99.2, 48.0, 45.4. MS-ESI (m/z) $[M + H]^+$ 347.14.

*3-(6-methoxyimidazo[1,2-a]pyrazin-2-yl)-7-(piperazin-1-yl)-2H-chromen-2-one. (**11**)*

Following procedures for compound **10**, from 3-(2-bromoacetyl)-7-fluoro-2*H*-chromen-2-one (30 mg, 0.1 mmol) and 5-methoxypyrazin-2-amine (13 mg, 0.1 mmol), 8 mg of compound **11** was obtained as yellow solid (two steps yield, 20.1%). ^{1}H NMR (500 MHz, DMSO-$_{d6}$): δ 8.82 (s, 1H), 8.76 (s, 1H), 8.60 (s, 1H), 8.31 (d, J = 1.5 Hz, 1H), 7.70 (d, J = 9.0 Hz, 1H), 7.02 (dd, J = 9.0, 2.5 Hz, 1H), 6.85 (d, J = 2 Hz, 1H), 3.87 (s, 3H), 3.32 (t, J = 5 Hz, 4H), 2.83 (t, J = 5 Hz, 4H); ^{13}C NMR (125 MHz, DMSO-$_{d6}$): δ 159.7, 155.3, 154.0, 152.7, 141.6, 139.7, 138.8,

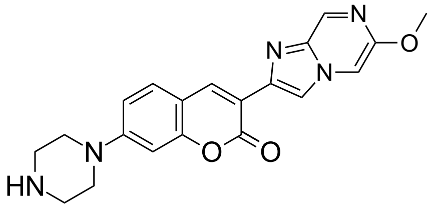

138.3, 129.9, 113.7, 111.7, 109.9, 102.4, 99.1, 55.0, 47.7, 45.3. MS-ESI (m/z) $[M + H]^+$ 378.17.

7. Tables and Figures

Table S1. Summary of GaMD simulations performed on the DNA and RNA Seq6 in the presence of the drug compound **1**.

System	N_{atoms}[a]	ID	Length(ns)	ΔV_{avg}[b] (kcal/mol)	$\sigma_{\Delta V}$[c] (kcal/mol)
DNA : compound **1**	38,192	Sim 1	500	11.54	3.40
		Sim 2	500	10.85	3.29
		Sim 3	500	11.36	3.33
		Sim 4	500	11.12	3.34
		Sim 5	500	11.50	3.45
RNA : compound **1**	38,113	Sim 1	500	12.39	3.44
		Sim 2	500	12.03	3.43
		Sim 3	500	11.89	3.40
		Sim 4	500	11.99	3.39
		Sim 5	500	12.20	3.43

[a] N_{atoms}: number of atoms in the system

[b] ΔV_{avg}: average of the GaMD boost potential

[c] $\sigma_{\Delta V}$: standard deviation of the GaMD boost potential

Table S2. Binding affinities between SMN-C2 and 9- and 10-nt DNAs containing the putative binding sequences in the fluorescence polarization (FP) assay.

Seq#	Sequence	K_d (μM)
Seq2	d(GAAGGAAGG)	34.3 ± 9.6
S26	d(TGAAGGAAGG)	6.2 ± 1.0
S27	d(AGAAGGAAGG)	11.6 ± 1.7

Table S3. Binding affinities between SMN-C2 and 18-nt DNA sequences determined by the fluorescence polarization (FP) assay.

Seq#	DNA sequence (18 nt)	K_d (μM)[a]	Fit SeqCS[b]
S1	AAAAGAAGGAAGGTGCTC	15.3 ± 0.6	Yes
S2	AAAAGAAGGAAGGAGCAC	20.2 ± 1.7	Yes
S3	AAAAGAAGGAAGGTGATA	21.7 ± 2.7	Yes
S4	AAAAAGAAGAAGGTGCTC	> 50	No
S5	AAAAGAAUGAAGGTGCTC	42.3 ± 4.2	No
S6	AAAAGAAGGAGGGTGCTC	33.3 ± 2.3	Yes
S7	AATAGAAGGAAGGTGCTC	17.3 ± 1.7	Yes
S8	AAATGAAGGAAGGTGCTC	12.4 ± 0.4	Yes

S9	AAAATAAGGAAGGTGCTC	56.2 ± 13.9	No
S10	AAAAGTAGGAAGGTGCTC	26.4 ± 2.2	No
S11	AAAAGATGGAAGGTGCTC	26.3 ± 1.2	No
S12	AAAAGAAGTAAGGTGCTC	37.4 ± 6.7	No
S13	AAAAGAAGGTAGGTGCTC	26.4 ± 2.2	No
S14	AAAAGAAGGATGGTGCTC	19.5 ± 2.3	No
S15	AAAAGAAGGAATGTGCTC	31.4 ± 4	No
S16	AAAAGAAGGAAGTTGCTC	48.9 ± 12.2	No
S17	AAAGGAAGGAAGGTGCTC	16.5 ± 1.1	Yes
S18	AAAAGGAGGAAGGTGCTC	> 50	No
S19	AAAAGAGGGAAGGTGCTC	21.0 ± 1.1	Yes
S21	AAAAAAAGGAAGGTGCTC	> 50	No
S22	AAAAGAAAGAAGGTGCTC	44.7 ± 7.2	No
S23	AAAAGAAGAAAGGTGCTC	> 50	No
S24	AAAAGAAGGAAAGTGCTC	36.8 ± 11.4	No
S25	AAAAGAAGGAAGATGCTC	48 ± 7.8	No

[a] The binding affinity was determined using florescence polarization assay (see Methods). The assay was duplicated, and the range was calculated with 95% confidence interval using Prism software after curve-fitting (Sigmoidal, 4 parameters).
[b] The consensus sequence SeqCS = NGARGGARGGN (R = A or G; N = A, T, G, or C). All sequences with a K_d < 25 μM concur with the consensus sequence (SeqSC) except S6 in the table (K_d = 33.3 ± 2.3 μM). All sequences that do not match the SeqSC has K_d > 25 μM.

Table S4. Binding affinities between RNA Seq4 and SMN-C2, compound **1**, and compound **2** in fluorescence polarization (FP) assay.

**Compound**	**Seq4**	**K_d (μM)**
SMN-C2	AAGAAGGAAGGUGC	21.7 ± 4.3
**1**	AAGAAGGAAGGUGC	25.4 ± 7.3
**2**	AAGAAGGAAGGUGC	37.5 ± 14.0

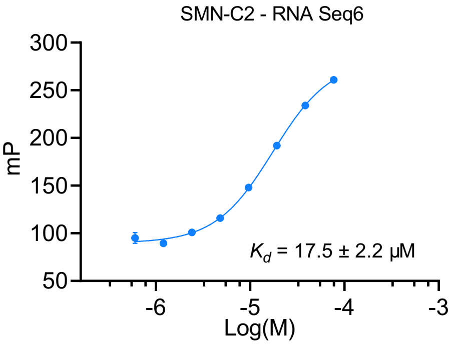
SMN-C2 - RNA Seq6
mP
Log(M)
K_d = 17.5 ± 2.2 μM

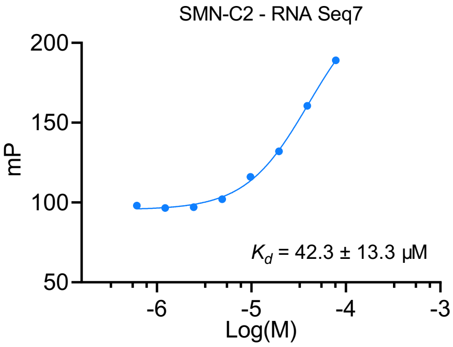
SMN-C2 - RNA Seq7
mP
Log(M)
K_d = 42.3 ± 13.3 μM

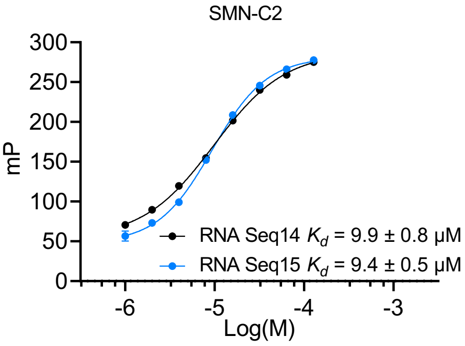
SMN-C2
mP
Log(M)
RNA Seq14 K_d = 9.9 ± 0.8 μM
RNA Seq15 K_d = 9.4 ± 0.5 μM

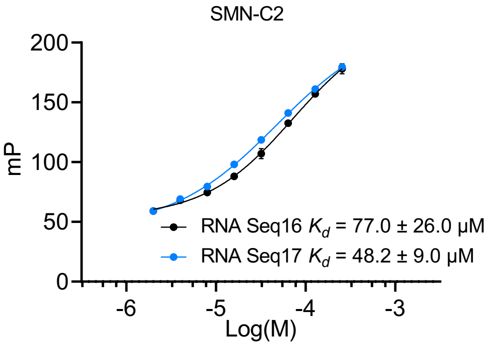
SMN-C2
mP
Log(M)
RNA Seq16 K_d = 77.0 ± 26.0 μM
RNA Seq17 K_d = 48.2 ± 9.0 μM

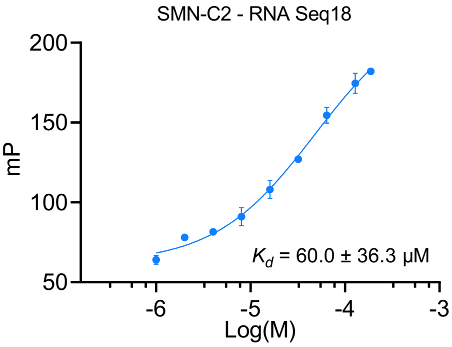
SMN-C2 - RNA Seq18
mP
Log(M)
K_d = 60.0 ± 36.3 μM

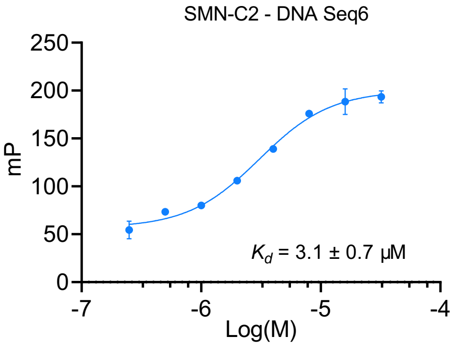
SMN-C2 - DNA Seq6
mP
Log(M)
K_d = 3.1 ± 0.7 μM

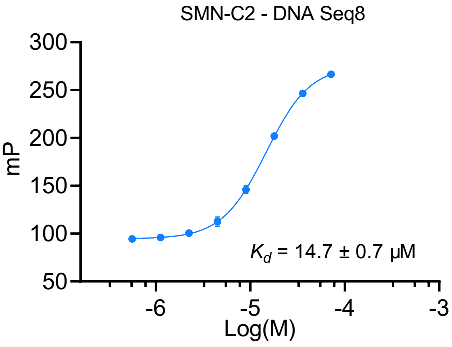
SMN-C2 - DNA Seq8
mP
Log(M)
K_d = 14.7 ± 0.7 μM

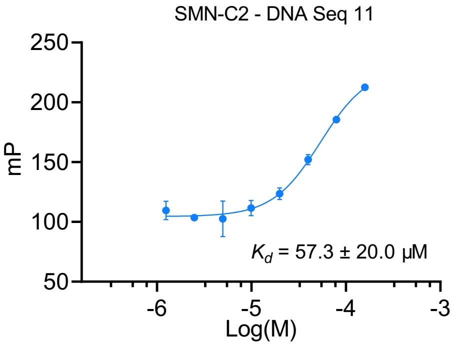
SMN-C2 - DNA Seq 11
mP
Log(M)
K_d = 57.3 ± 20.0 μM

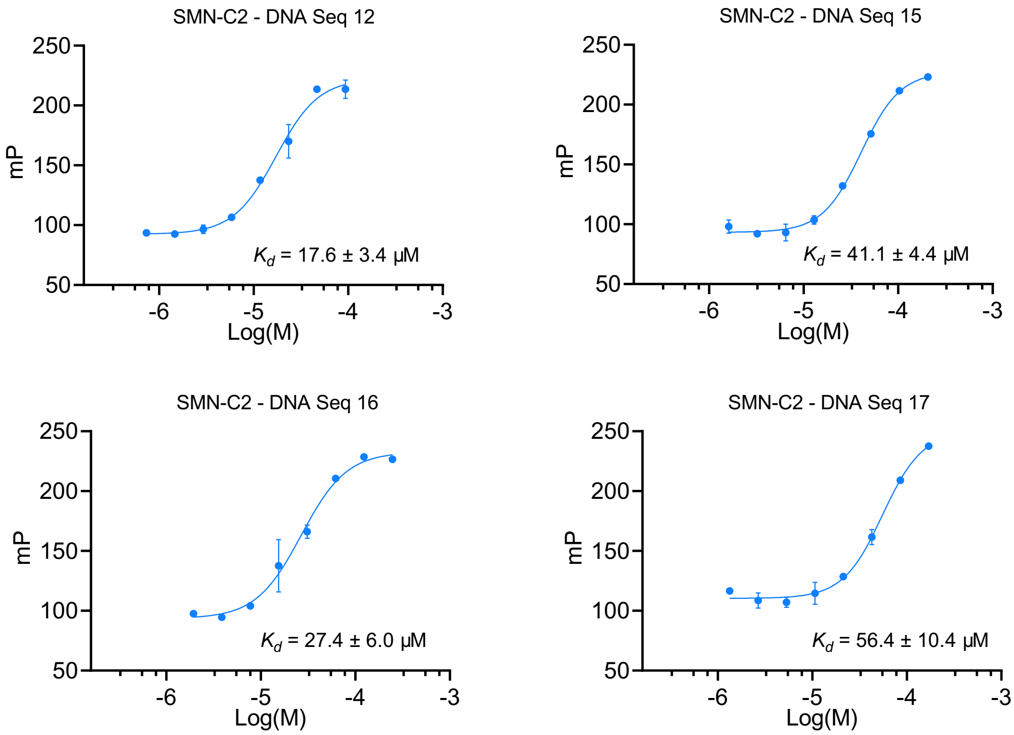

Figure S1. The fluorescence polarization (FP) assay dose-response curves for sequences with K_d values in **Table 1**, plotted using GraphPad Prism 8.

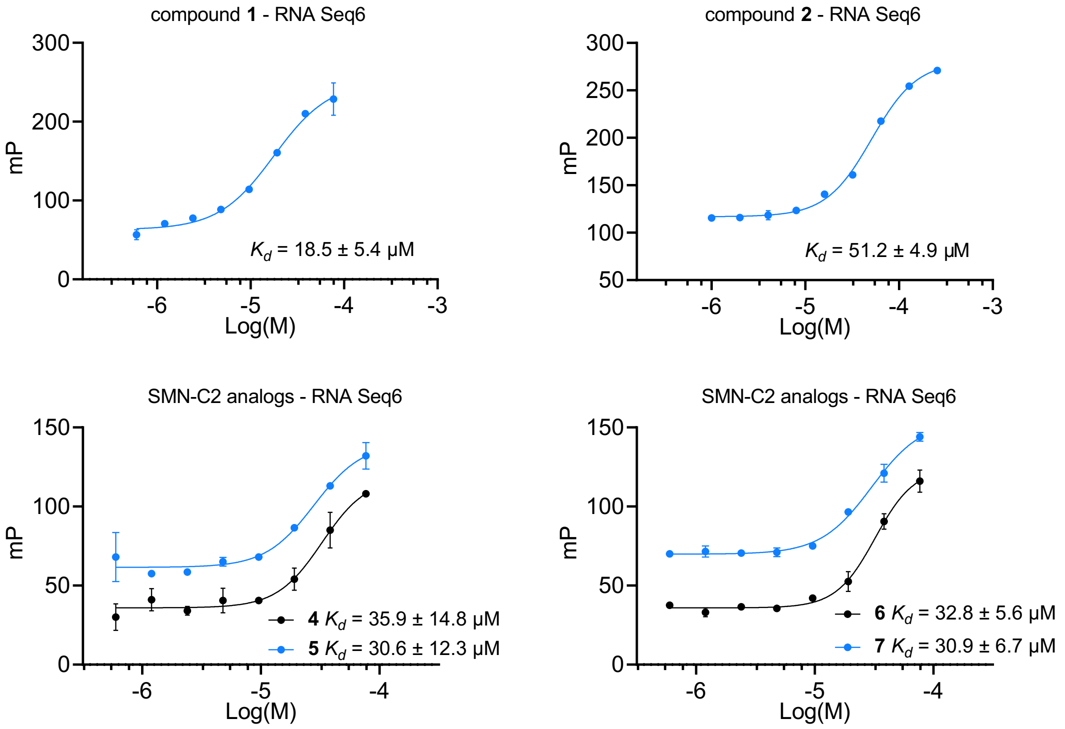

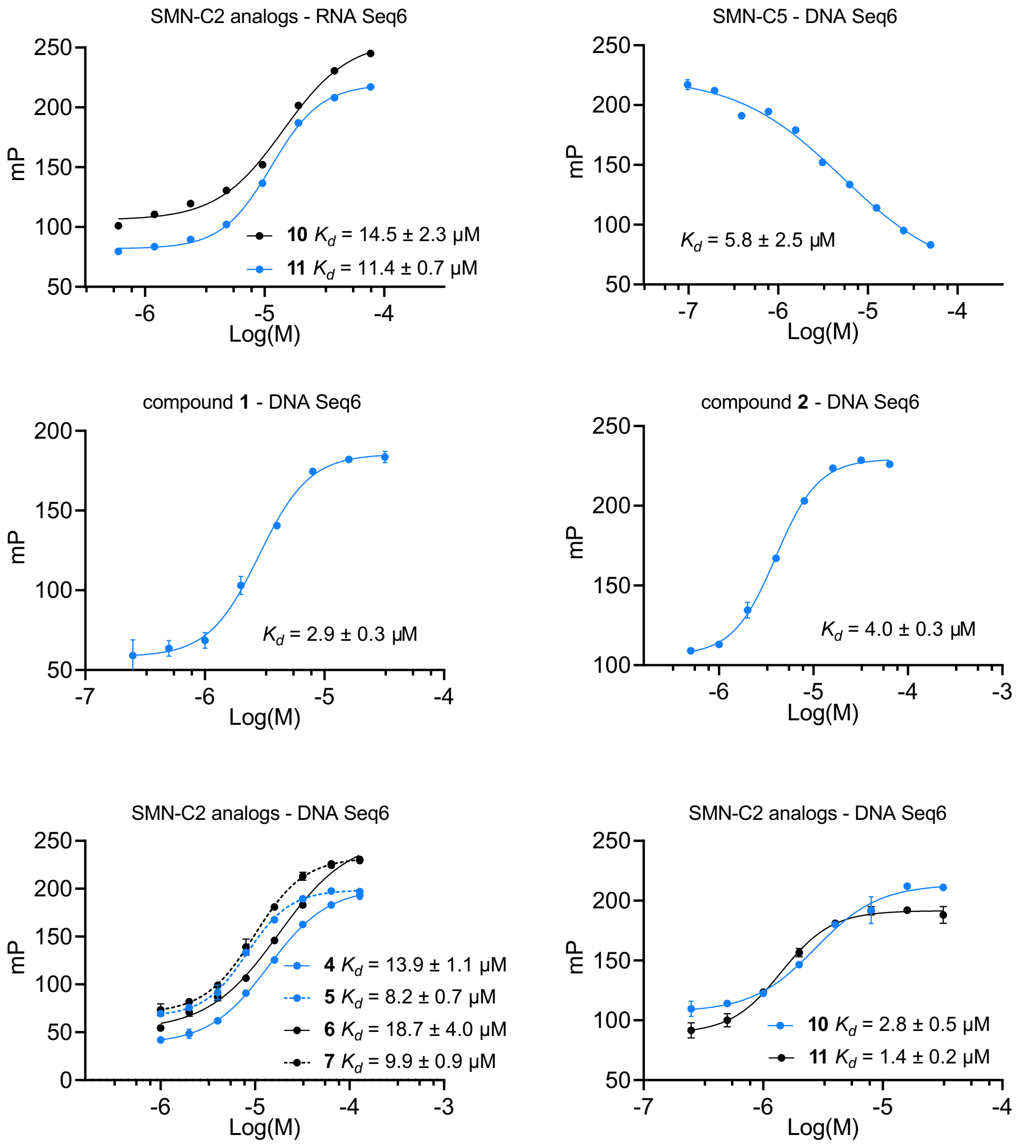

Figure S2. The fluorescence polarization (FP) assay dose-response curves for compounds with K_d values in **Table 2**, plotted using GraphPad Prism 8.

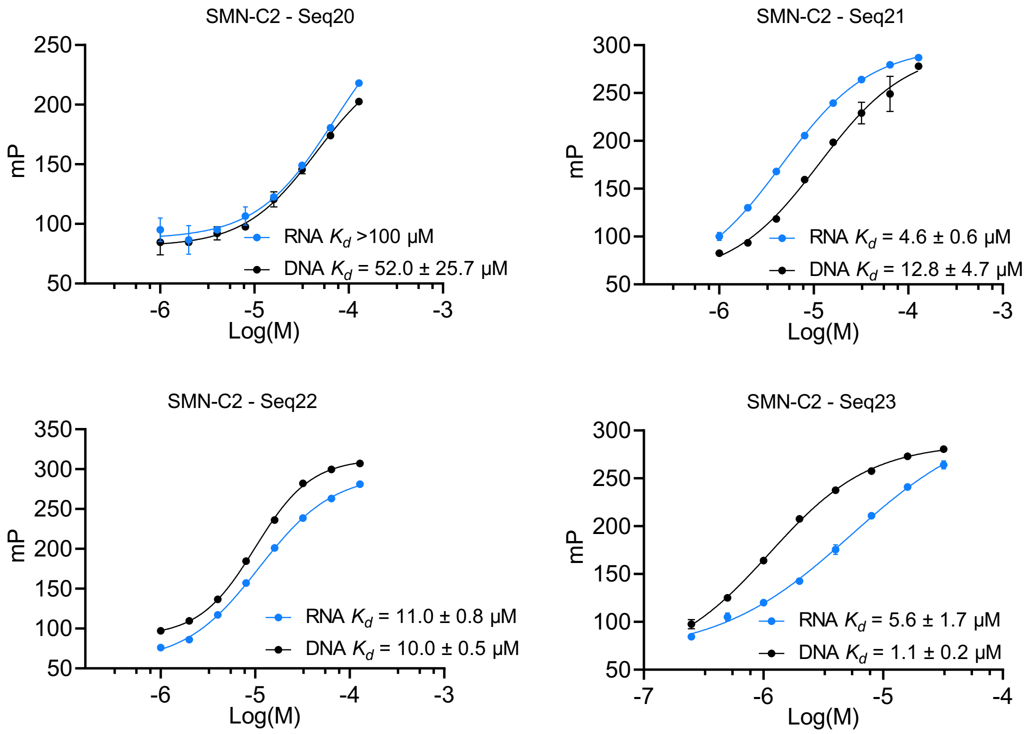

Figure S3. The fluorescence polarization (FP) assay dose-response curves for sequences with K_d values in **Scheme 2**, plotted using GraphPad Prism 8.

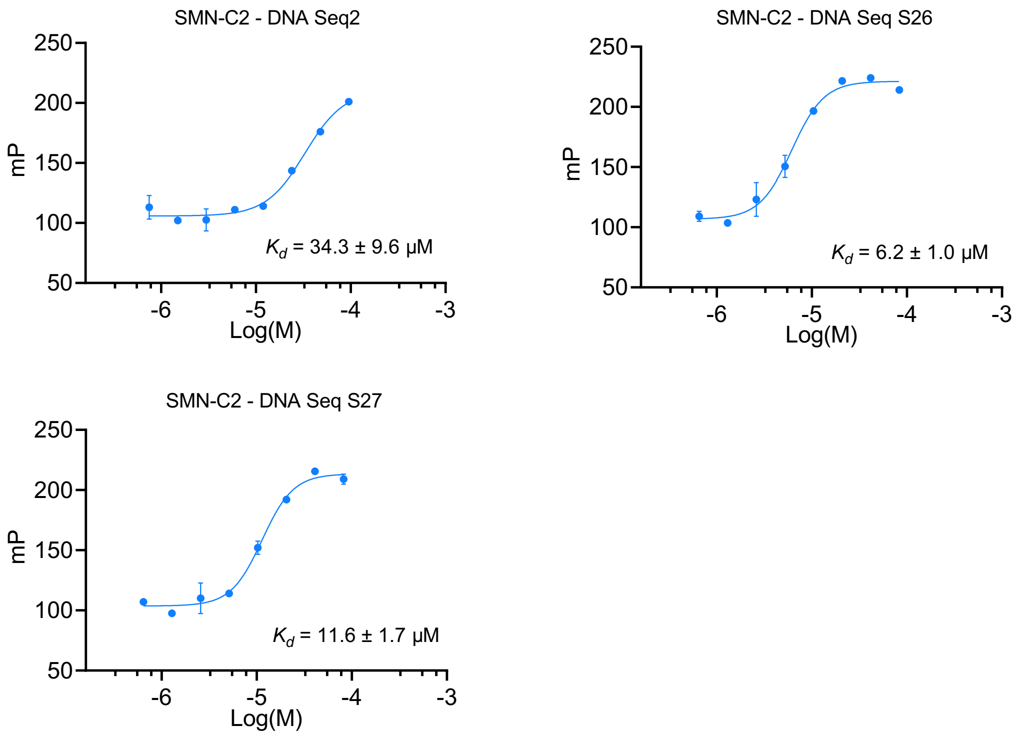

Figure S4. The fluorescence polarization assay dose-response curves for sequences in **Table S2**, plotted using GraphPad Prism 8.

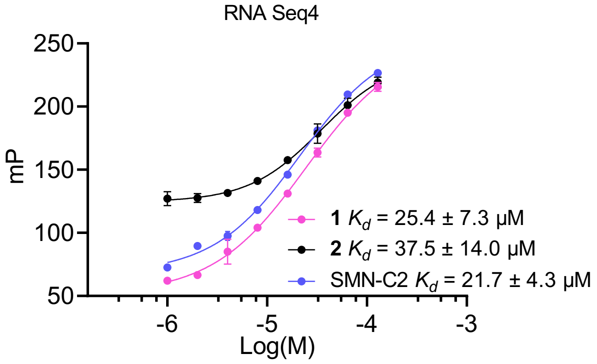

Figure S5. The fluorescence polarization assay dose-response curves for compounds in **Table S4**, plotted using GraphPad Prism 8.

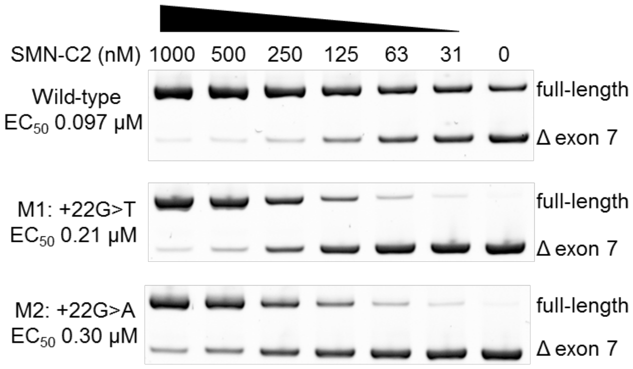

Figure S6. Cell-based SMN2 splicing assay with minigenes that harbor different mutations in the GA-rich sequence. The minigene-transfected 293T cells were treated with different concentrations of SMN-C2 for 24 h. The EC_{50} was calculated using the disappearance of the Δexon 7 band. The figure is a representative of three biological replicates.

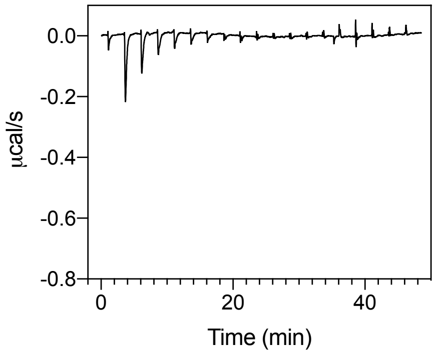

Figure S7. ITC raw data for SMN-C2 (300 μM) and dsDNA (43 μM, annealed Seq6 and its reverse complememt) in a buffer that contains 30 mM 2-(*N*-morpholino)ethanesulfonic acid (MES, pH 6.1), 5% DMSO, and 100 mM NaCl. The figures are representation of three independent experiments.

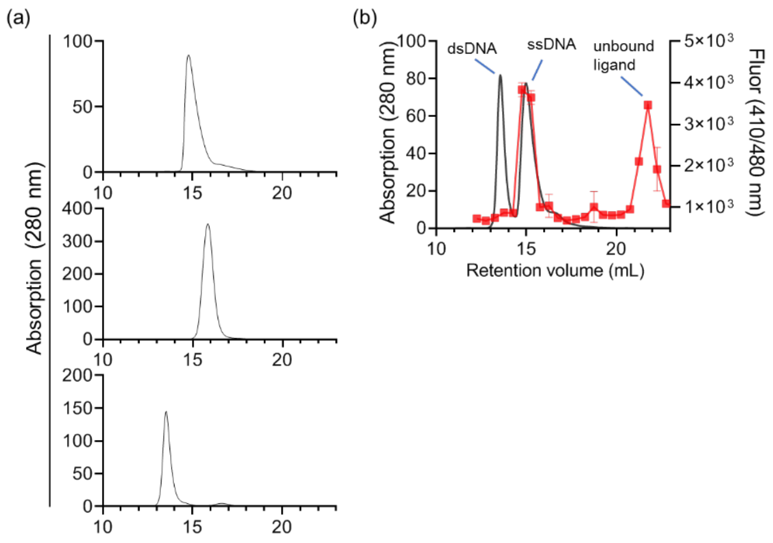

Figure S8. (a) Size-exclusion chromatography with a Superdex 75 column for DNAs Seq4 (50 μM), Seq4_RC (50 μM), and the dsDNA made by annealing equal molar of the DNAs Seq4 and Seq4_RC. (b) Size-exclusion chromatography with a Superdex 75 column for an annealed mixture of Seq4 (100 μM), Seq4_RC (50 μM), and SMN-C2 (100 μM) 1× phosphate buffered saline (PBS) with the absorption (black, absorption at 280 nm for DNA) and fluorescence readout (red, fluorescence at excitation/emission = 410/480 nm for SMN-C2). Excess amount of SMN-C2 was eluted at retention volume ~ 21 mL. The figures are the representation of three independent experiments.

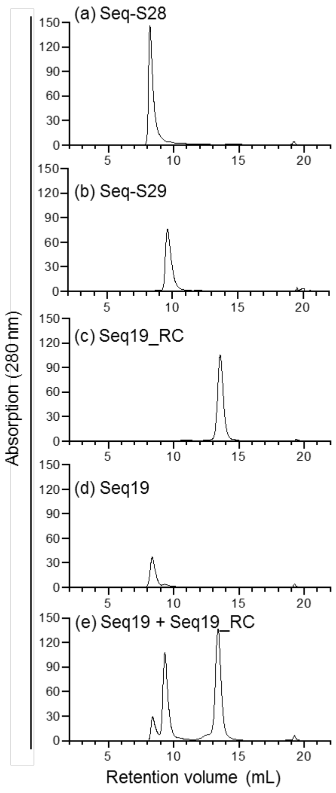

Figure S9. Size-exclusion chromatography with a Superdex 30 column for (a) random 45nt ssDNA, Seq S28 = 5'-TACAGATCTACTAGTGATCTATGACTGATCTGTACA-TGATCTACA, (b) random 22nt ssDNA, Seq S29 = 5'-CAGGTGTCCACTCCCAGT-TCAA, (c) reverse complement of Seq19, Seq19_RC = ACCCTCCCTCA, (d) Seq19 = 5'-TGAGGGAGGGT, and (e) 1:1 annealed mixture of Seq19 and Seq19_RC. All DNA samples were prepared in 1× phosphate buffered saline (PBS) at 100 μM.

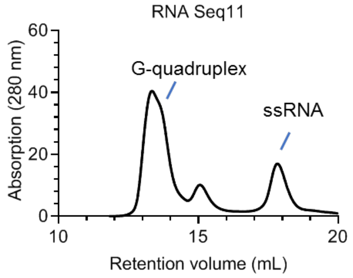

Figure S10. Size-exclusion chromatography with a Superdex 75 column for RNA Seq11.

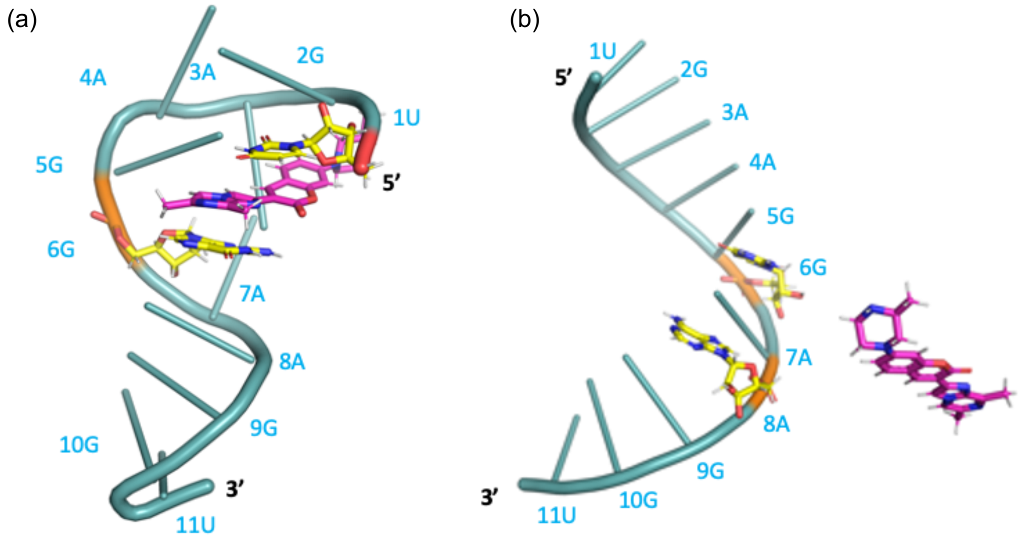

Figure S11. The (a) "Intermediate" and (b) "Unbound/Unfolded" conformational states of RNA-compound **1** obtained from the GaMD simulations.

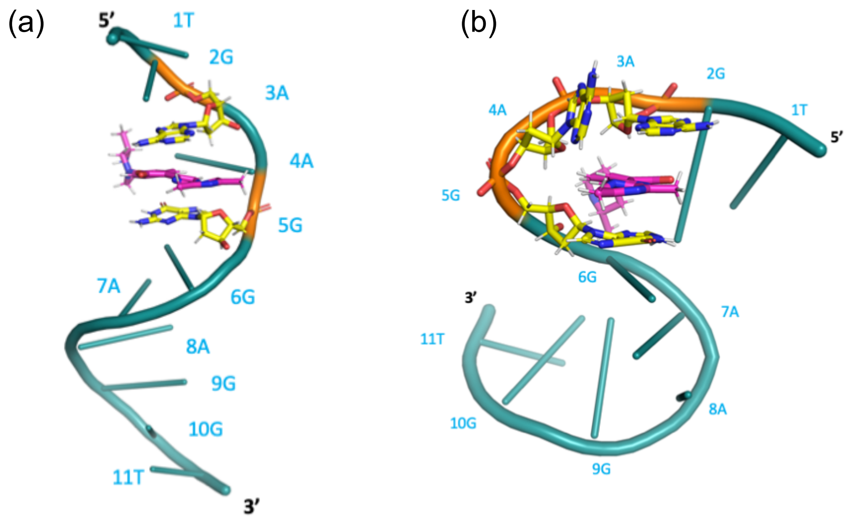

Figure S12. The (a) "Bound/Unfolded" and (b) "Intermediate" conformational states of DNA Seq6 during binding of compound **1** obtained from the GaMD simulations.

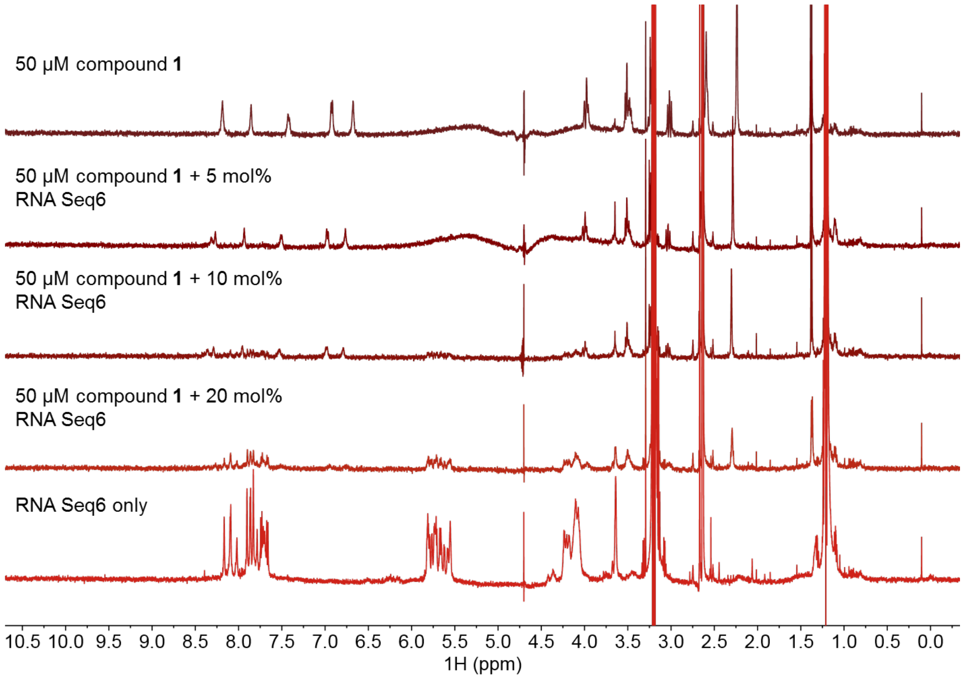

Figure S13. ^{1}H NMR spectra with ES (600 MHz) of 50 µM compound **1** solution and RNA Seq6 from 0-20 mol%.

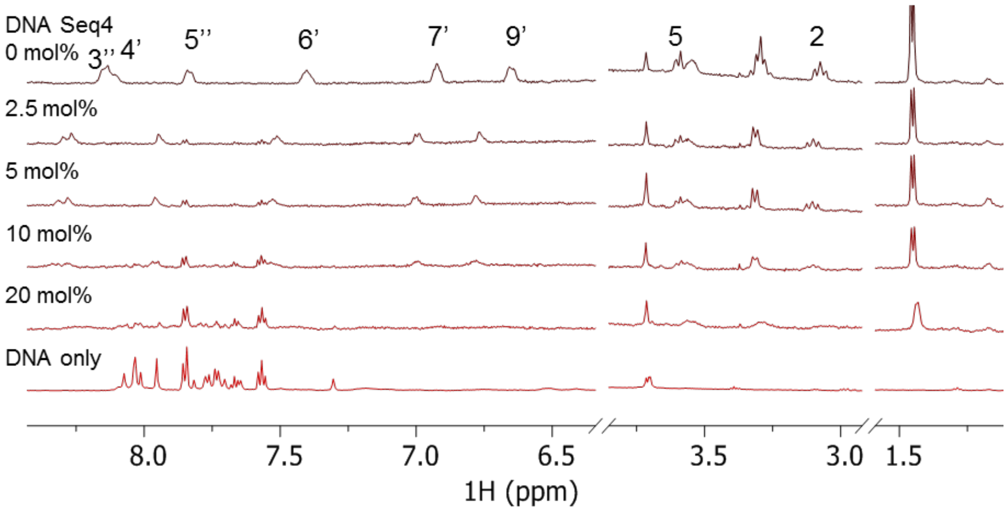

Figure S14. ^{1}H NMR titration of compound **1** (100µM) with DNA Seq4 at various concentrations, from top to bottom: 0 mol% (no DNA), 2.5 mol% DNA, 5 mol% DNA, 10 mol% DNA, 20 mol% DNA, and a control spectrum of DNA (20 µM) without compound **1**.

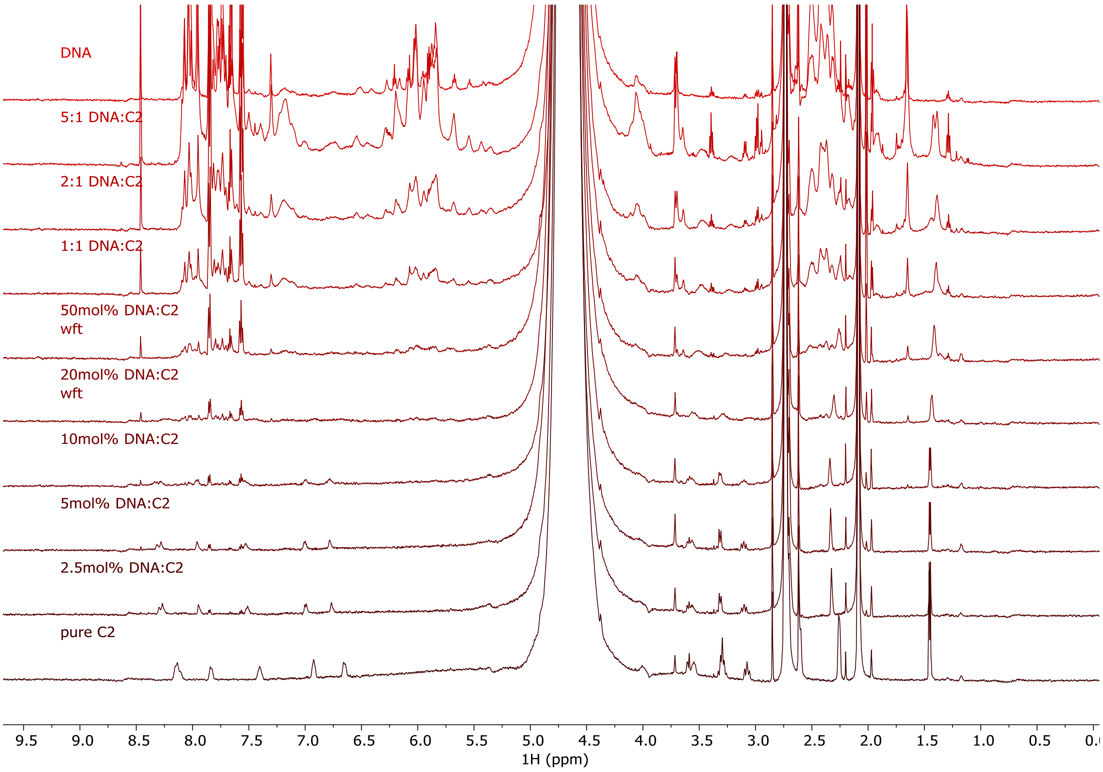

Figure S15. ^{1}H NMR spectra with wet1D (600 MHz) of 100 µM compound **1** solution and DNA Seq4 from 0-500 mol%.

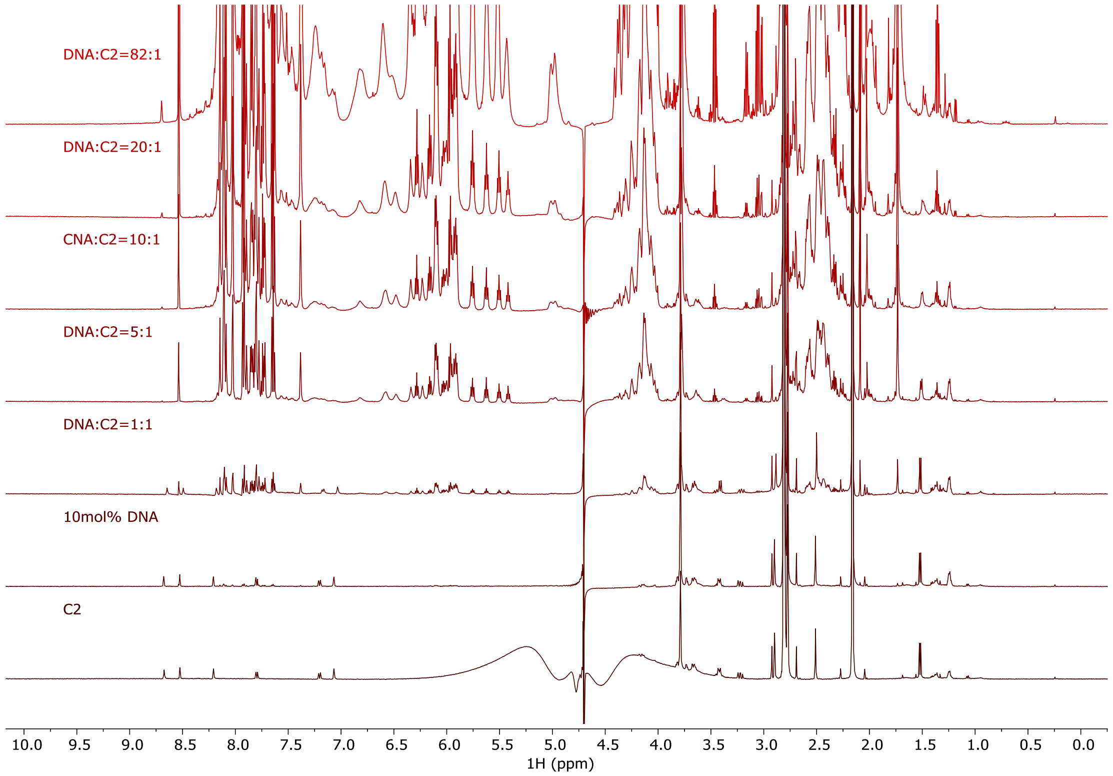

Figure S16. ^{1}H NMR spectra with ES (600 MHz) of 10 μM compound **1** solution with DNA Seq4 from 0-8200 mol%.

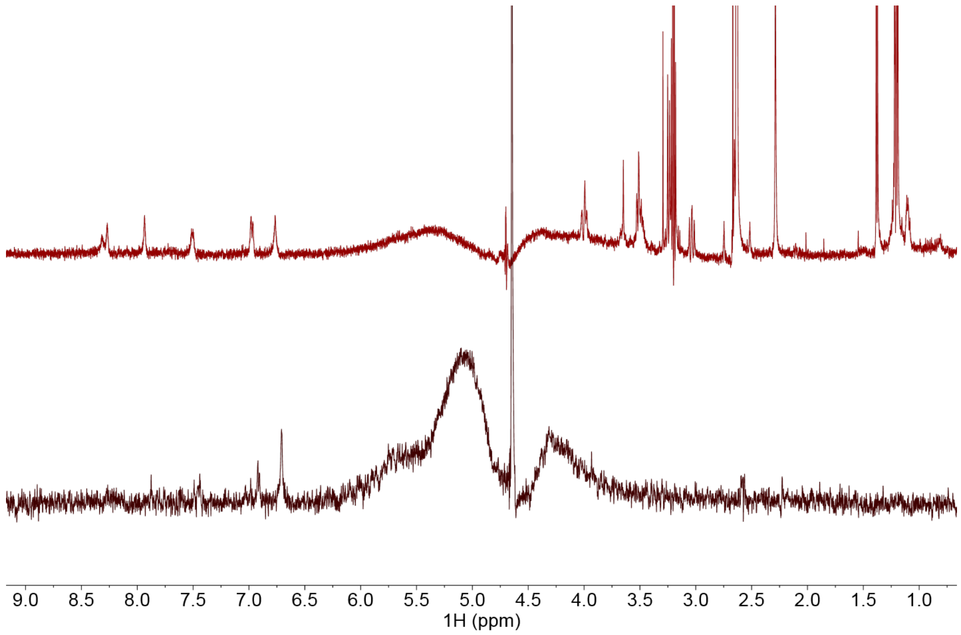

Figure S17. Comparison of STD (bottom) and ^{1}H NMR spectrum (top) of 50 μM compound **1** solution with 5 mol% RNA Seq6.

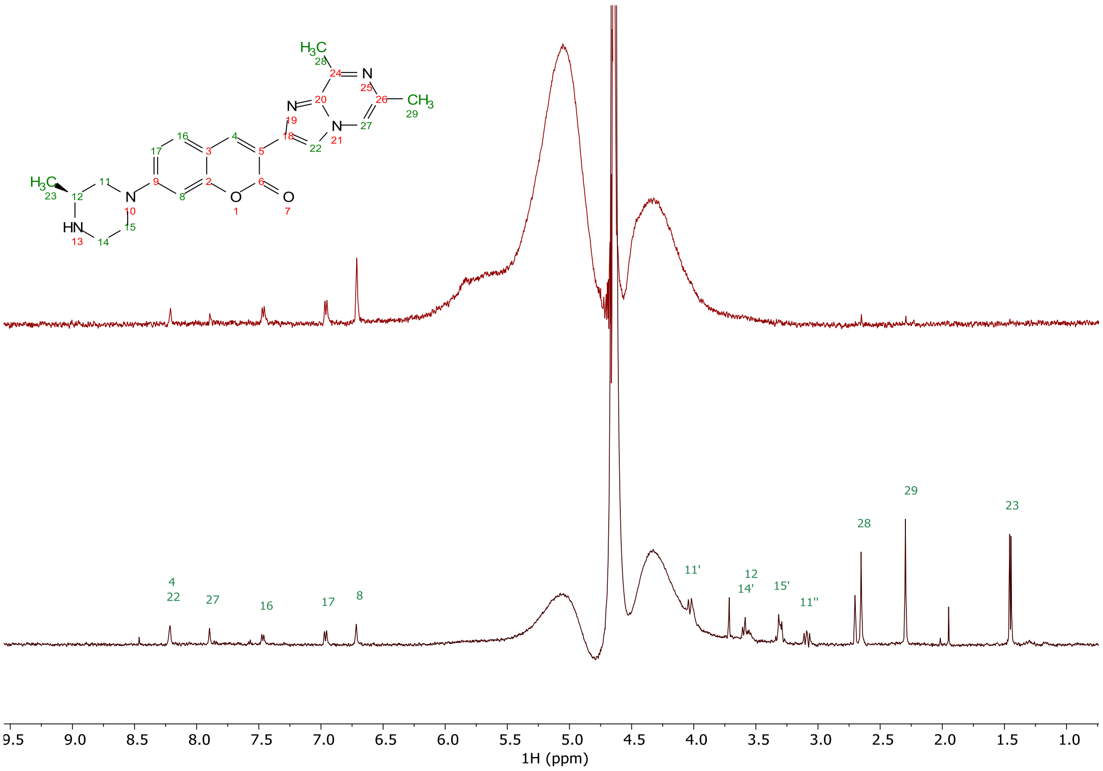

Figure S18. Comparison of STD (top) and ^{1}H NMR spectrum (bottom) of 10 µM compound **1** solution with 5 mol% DNA Seq 4.

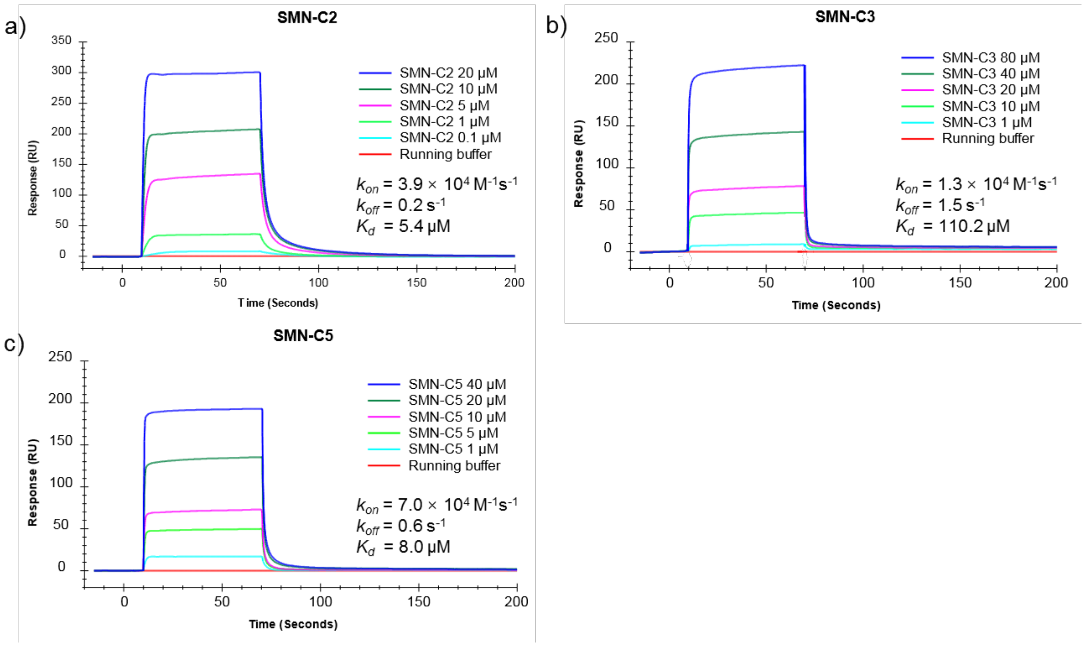

Figure S19. SPR kinetic evaluation results of (a) SMN-C2, (b) SMN-C3, and (c) SMN-C5 with RNA Seq4.

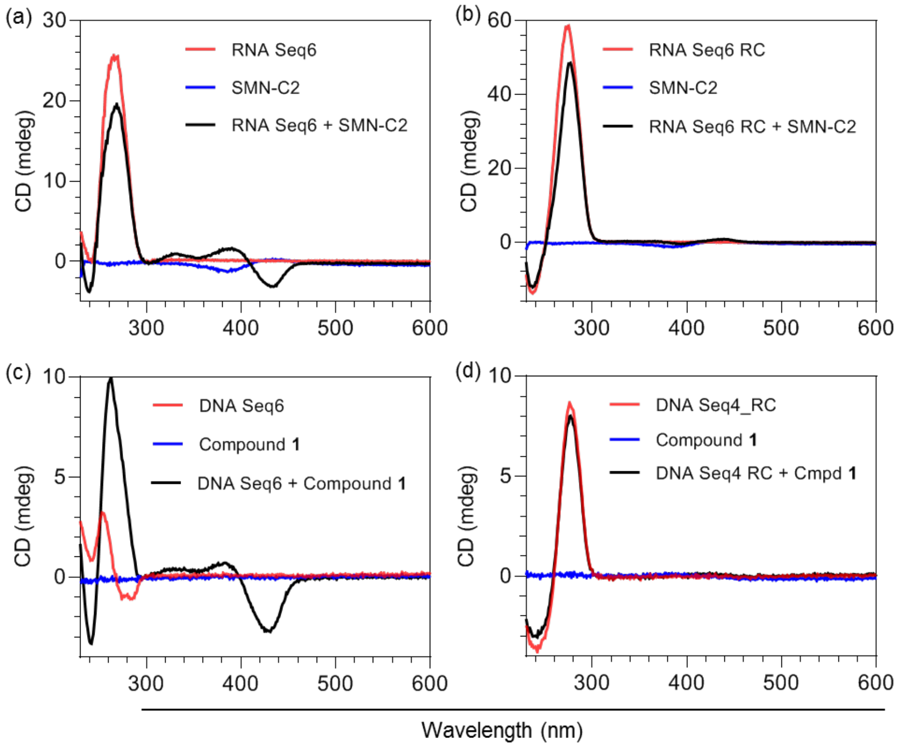

Figure S20. Circular dichroism of (a) RNA Seq6 and (b) reverse complement of RNA Seq6 (RNA Seq6 RC) in the presence or absence of SMN-C2 (262.5 μM); (c) DNA Seq6 and (d) reverse complement of DNA Seq4 (DNA Seq4 RC) in the presence or absence of compound **1** (60 μM). RNAs and DNAs were prepared at 175 μM, 40 μM respectively in 30 mM HEPES (pH 7.3) and 100 mM NaCl. HEPES = 4-(2-hydroxyethyl)-1-piperazineethanesulfonic acid.

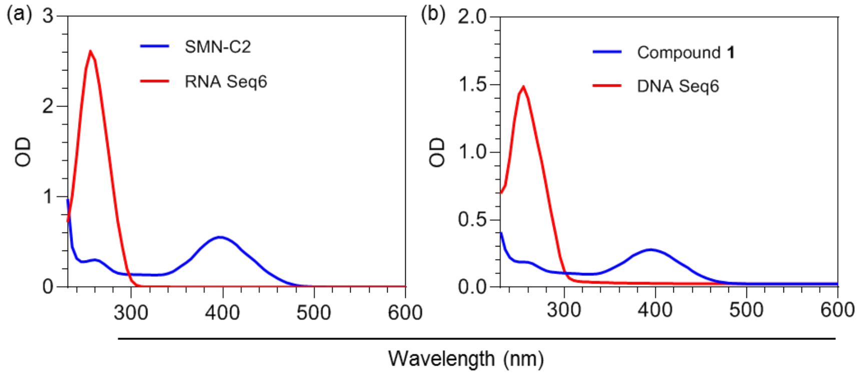

Figure S21. The ultraviolet-visible spectroscopy of (a) SMN-C2 (262.5 μM) and RNA Seq6 (175 μM); (b) compound **1** (60 μM) and DNA Seq6 (40 μM) in 30 mM HEPES (pH 7.3) and 100 mM NaCl.

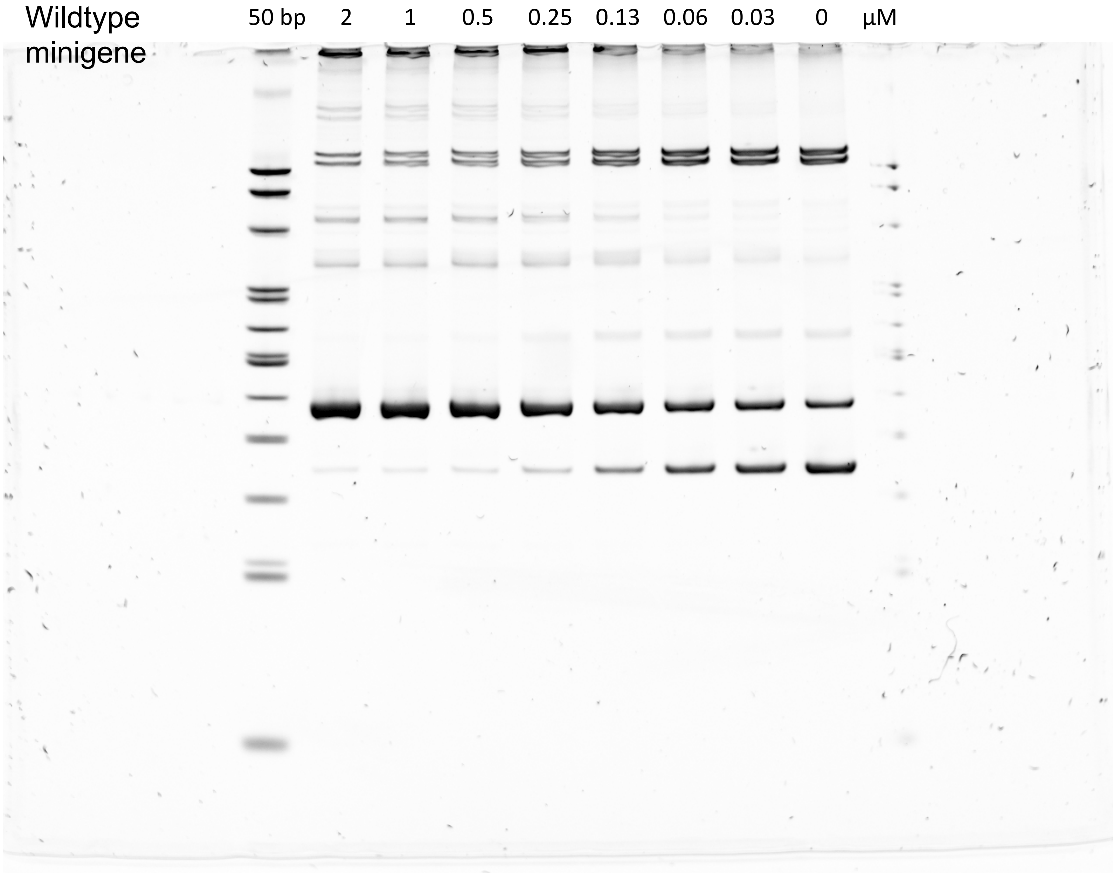
Wildtype minigene
50 bp
2
1
0.5
0.25
0.13
0.06
0.03
0
µM

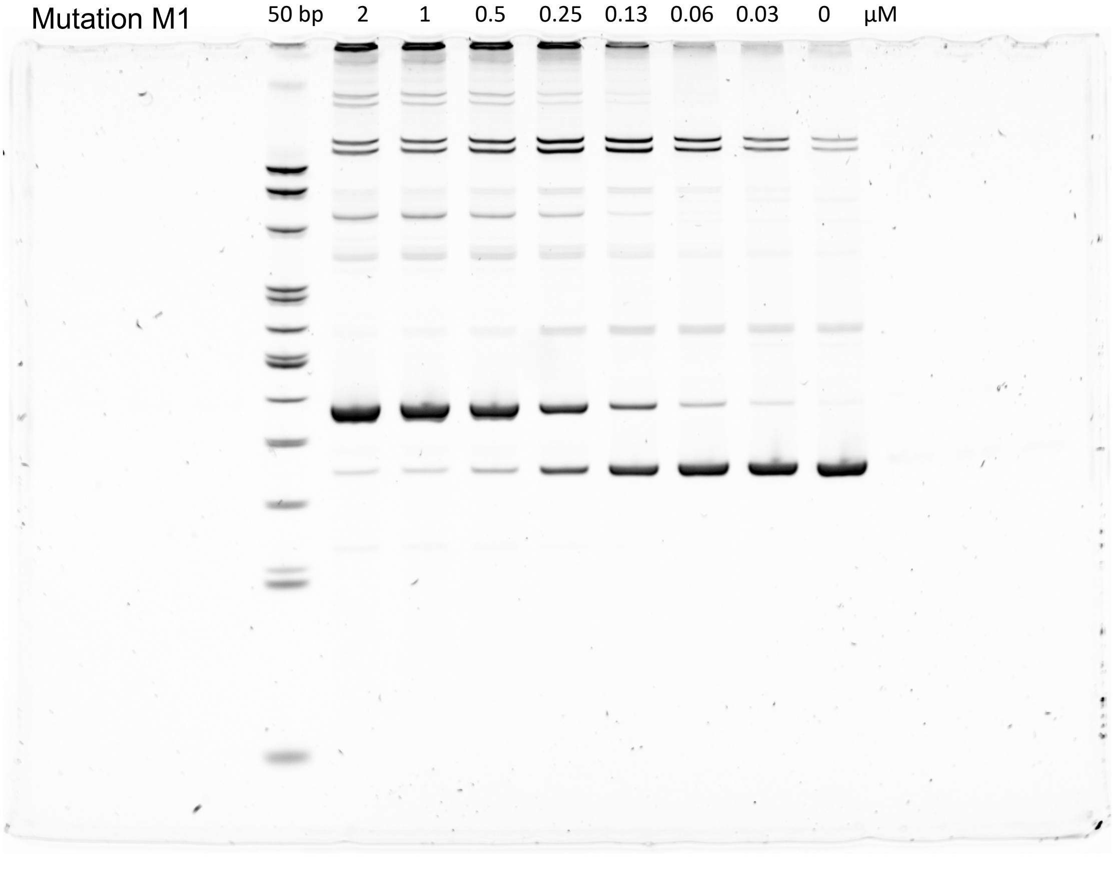
Mutation M1
50 bp
2
1
0.5
0.25
0.13
0.06
0.03
0
µM

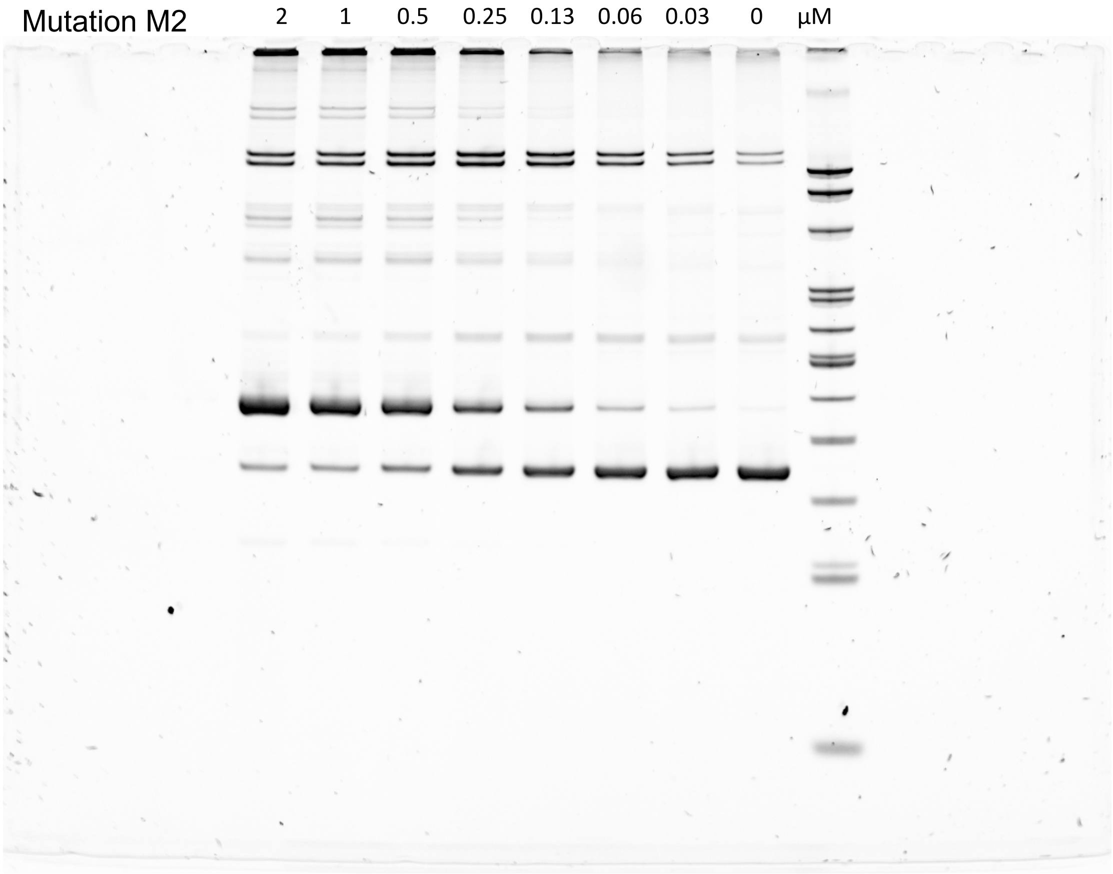

Figure S22. Full gel images for Figure S6.

8. ^{1}H and ^{13}C NMR Spectra and HPLC

Compound 2

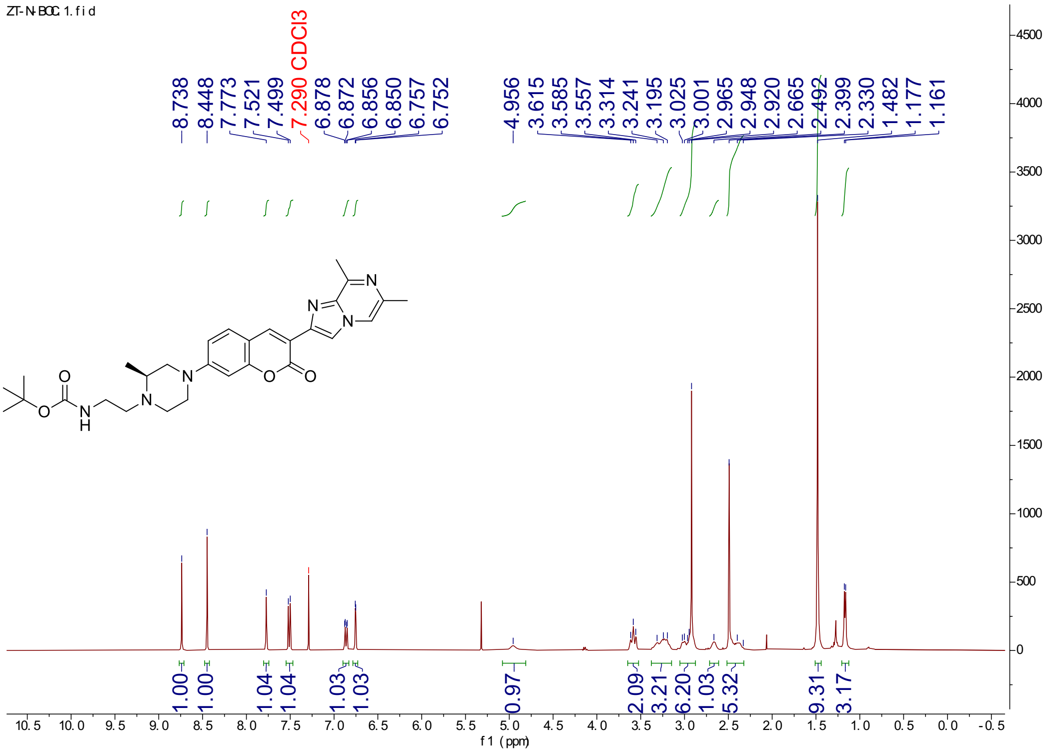
ZT-N-BOC 1.fid
8.738
8.448
7.773
7.521
7.499
7.290 CDCl3
6.878
6.872
6.856
6.850
6.757
6.752
4.956
3.615
3.585
3.557
3.314
3.241
3.195
3.025
3.001
2.965
2.948
2.920
2.665
2.492
2.399
2.330
1.482
1.177
1.161
1.00
1.00
1.04
1.04
1.03
1.03
0.97
2.09
3.21
6.20
1.03
5.32
9.31
3.17
f1 (ppm)

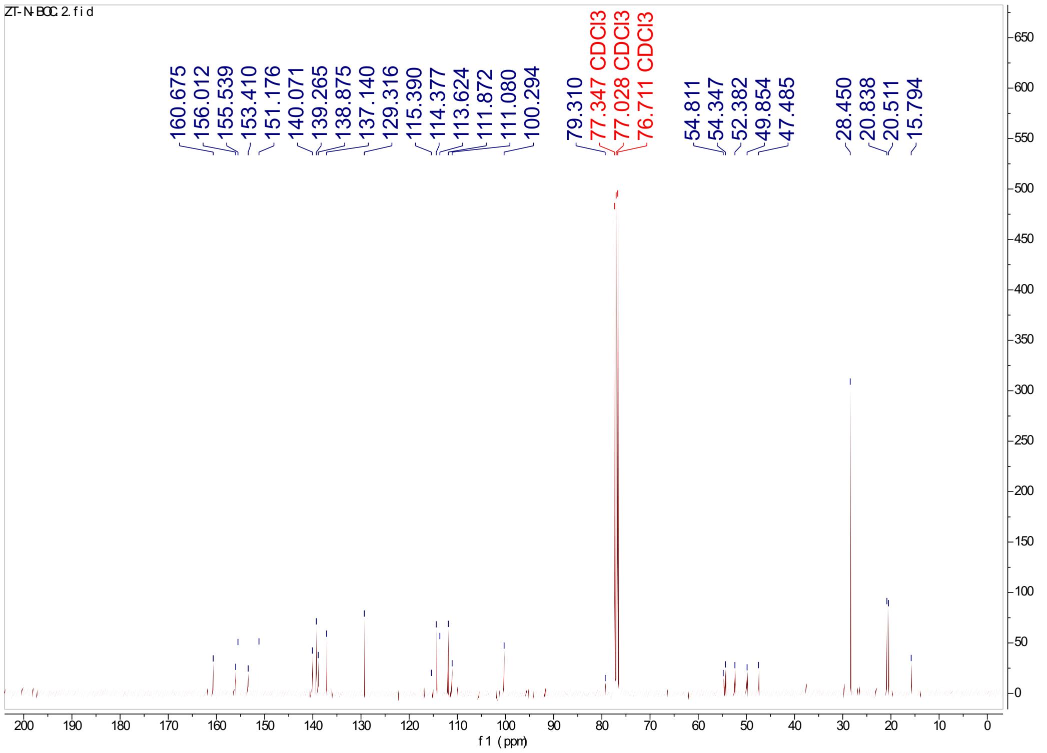
ZT-N-BOC 2.fid
160.675
156.012
155.539
153.410
151.176
140.071
139.265
138.875
137.140
129.316
115.390
114.377
113.624
111.872
111.080
100.294
79.310
77.347 CDCl3
77.028 CDCl3
76.711 CDCl3
54.811
54.347
52.382
49.854
47.485
28.450
20.838
20.511
15.794
f1 (ppm)

Compound 3

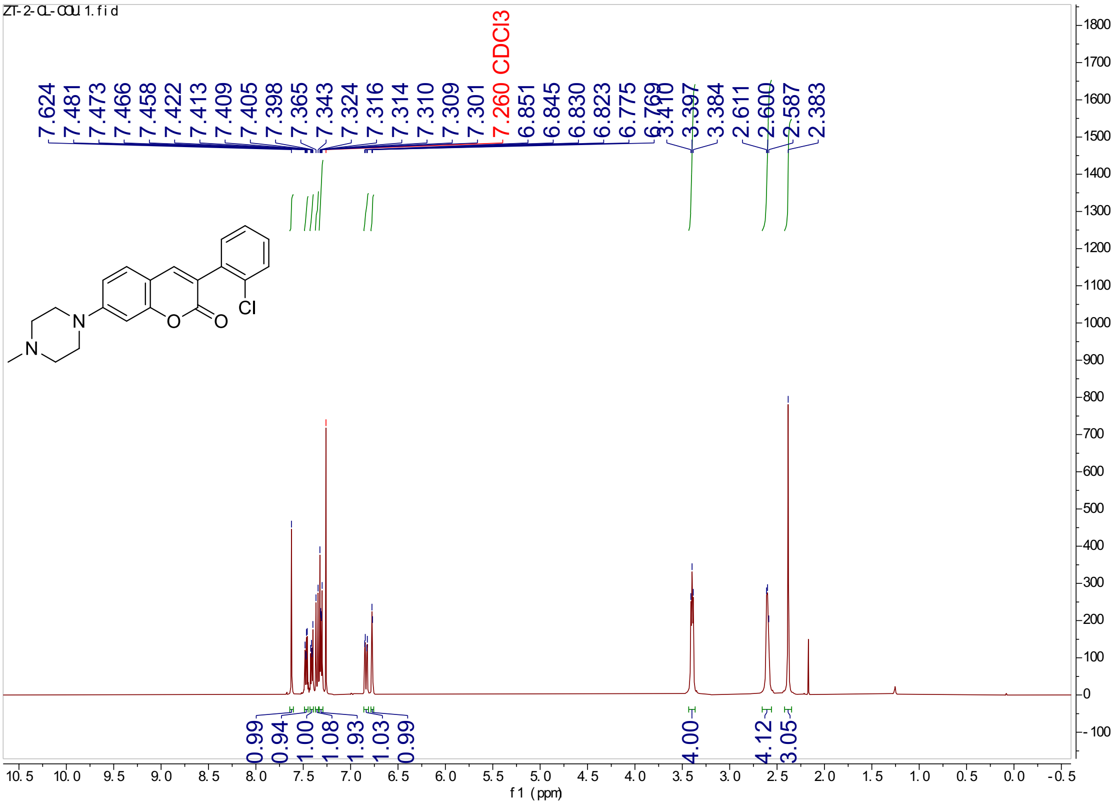

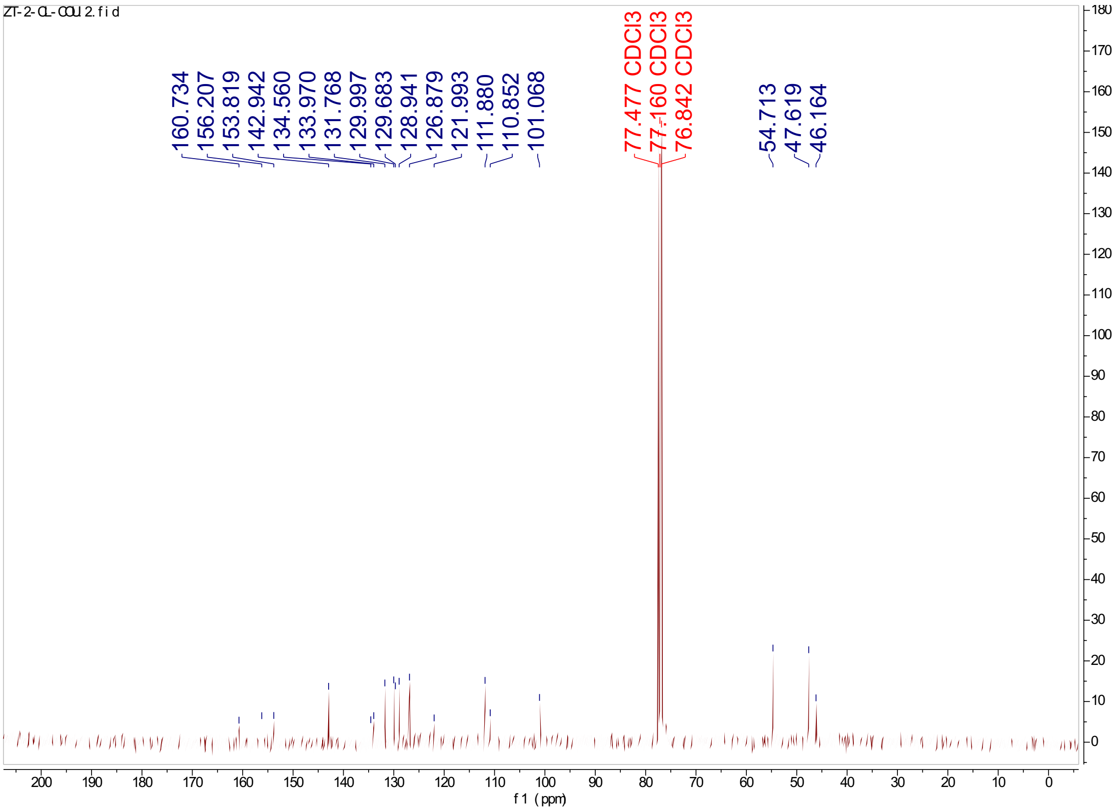

Compound 4

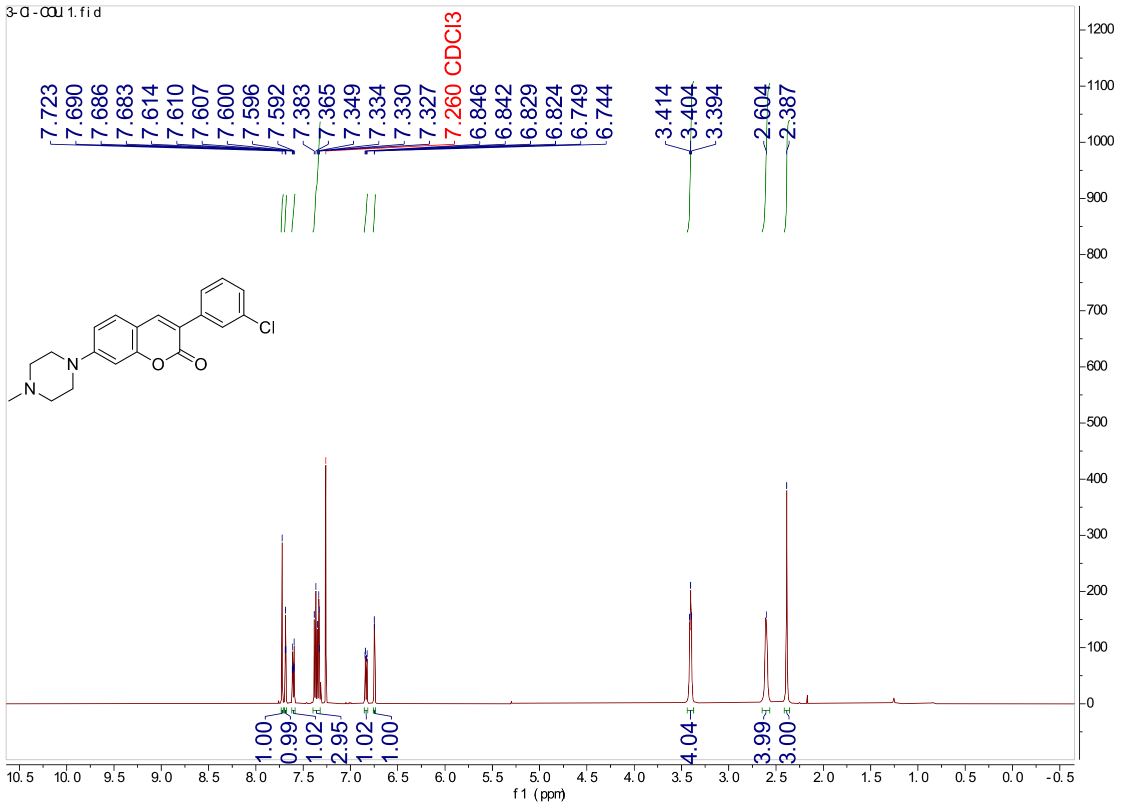
3-Cl-COU 1.fid
7.723 7.690 7.686 7.683 7.614 7.610 7.607 7.600 7.596 7.592 7.383 7.365 7.349 7.334 7.330 7.327 7.260 CDCl3 6.846 6.842 6.829 6.824 6.749 6.744
3.414 3.404 3.394
2.604 2.387
Cl
N
N
O
O
1.00 0.99 1.02 2.95 1.02 1.00
4.04 3.99 3.00
f1 (ppm)

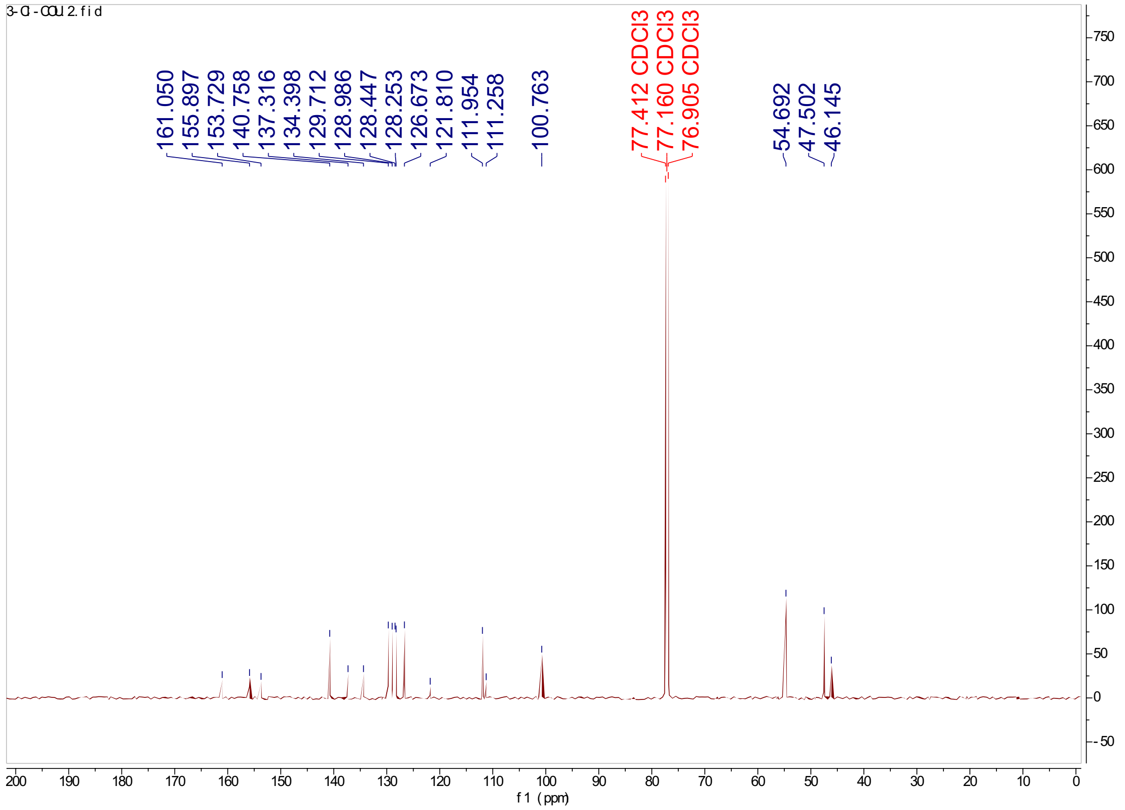
3-Cl-COU 2.fid
161.050 155.897 153.729 140.758 137.316 134.398 129.712 128.986 128.447 128.253 126.673 121.810 111.954 111.258 100.763
77.412 CDCl3 77.160 CDCl3 76.905 CDCl3
54.692 47.502 46.145
f1 (ppm)

Compound 5

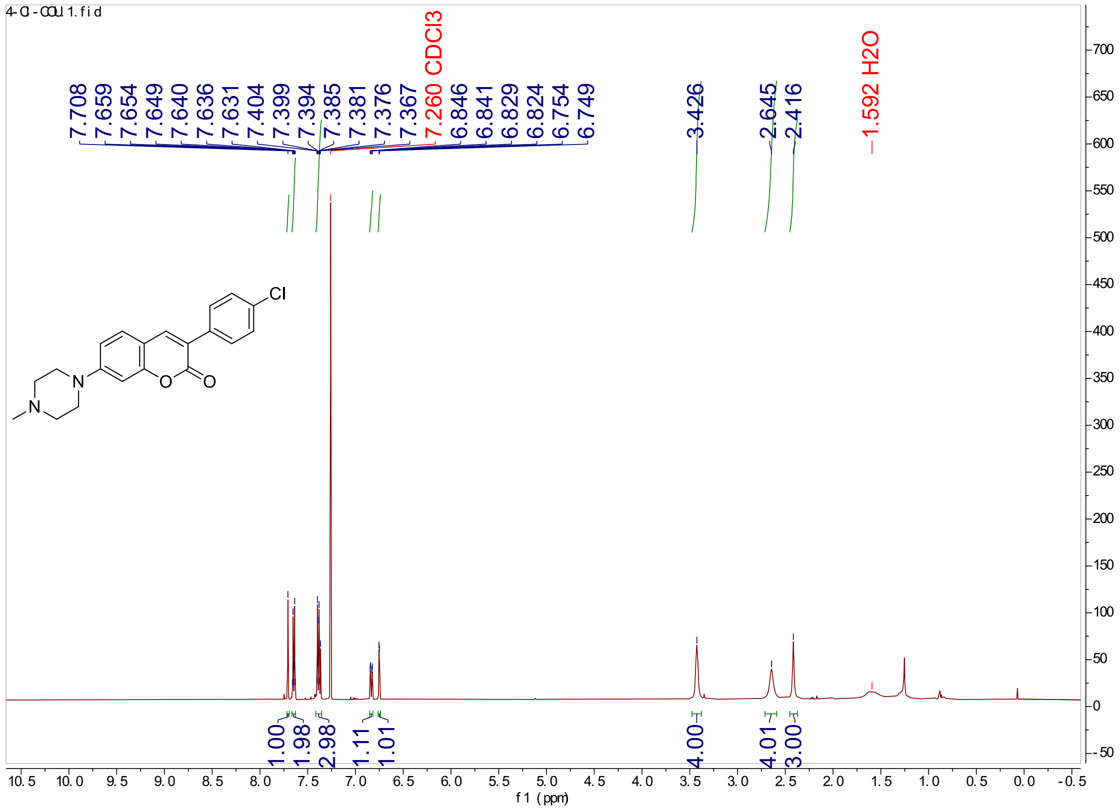
4-Cl-COU1.fid
7.708
7.659
7.654
7.649
7.640
7.636
7.631
7.404
7.399
7.394
7.385
7.381
7.376
7.367
7.260 CDCl3
6.846
6.841
6.829
6.824
6.754
6.749
3.426
2.645
2.416
1.592 H2O
Cl
N
N
O
O
1.00
1.98
2.98
1.11
1.01
4.00
4.01
3.00
f1 (ppm)

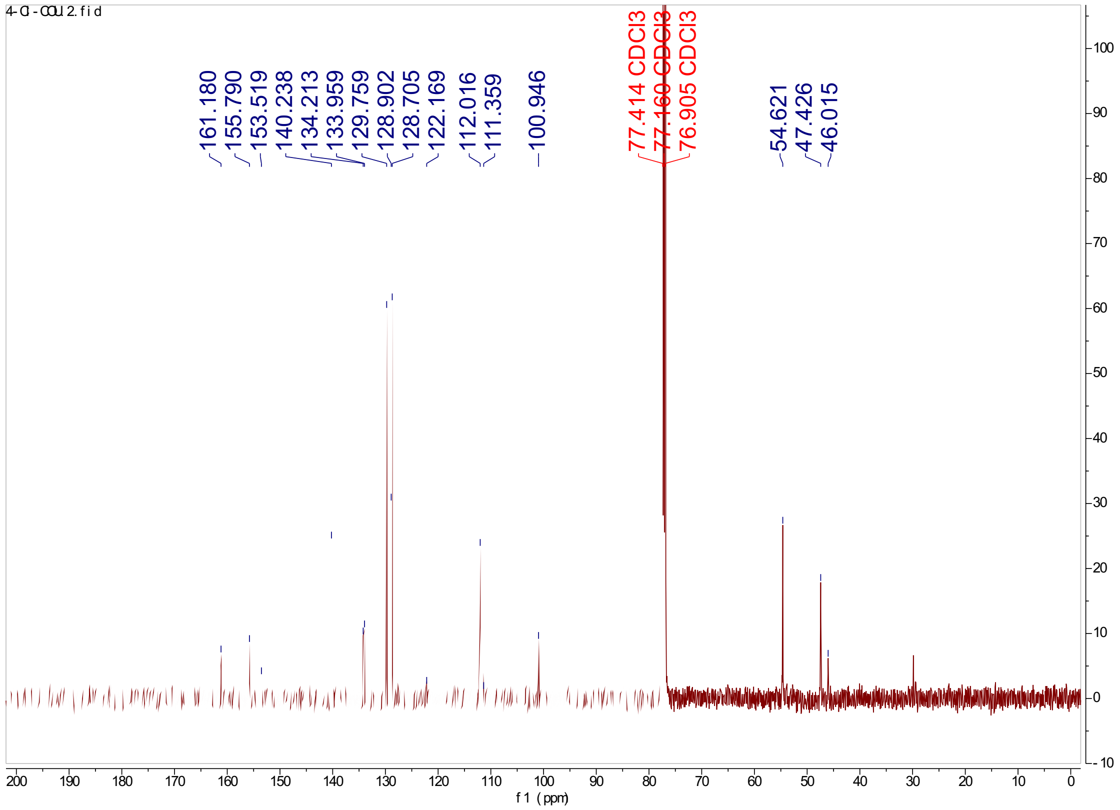
4-Cl-COU2.fid
161.180
155.790
153.519
140.238
134.213
133.959
129.759
128.902
128.705
122.169
112.016
111.359
100.946
77.414 CDCl3
77.160 CDCl3
76.905 CDCl3
54.621
47.426
46.015
f1 (ppm)

Compound 6

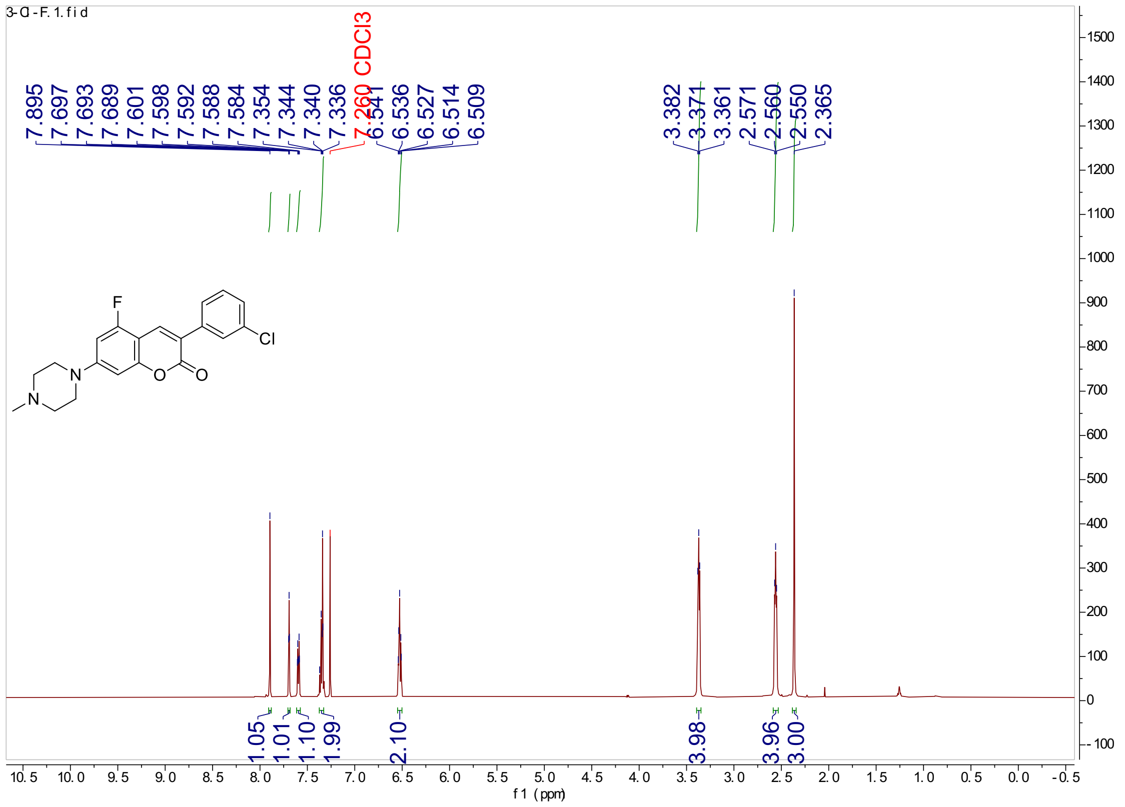
3-Cl-F.1.fid
7.895
7.697
7.693
7.689
7.601
7.598
7.592
7.588
7.584
7.354
7.344
7.340
7.336
7.260 CDCl3
6.541
6.536
6.527
6.514
6.509
3.382
3.371
3.361
2.571
2.560
2.550
2.365
F
Cl
N
N
O
O
1.05
1.01
1.10
1.99
2.10
3.98
3.96
3.00
f1 (ppm)

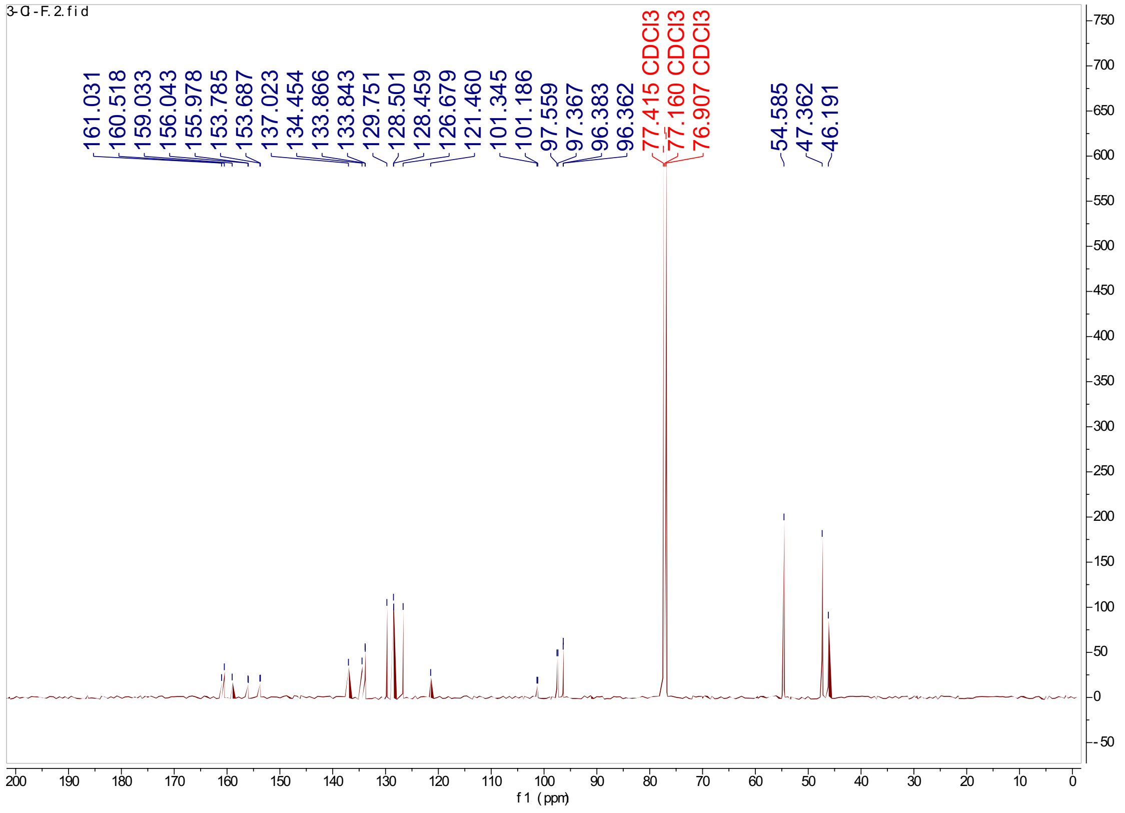
3-Cl-F.2.fid
161.031
160.518
159.033
156.043
155.978
153.785
153.687
137.023
134.454
133.866
133.843
129.751
128.501
128.459
126.679
121.460
101.345
101.186
97.559
97.367
96.383
96.362
77.415 CDCl3
77.160 CDCl3
76.907 CDCl3
54.585
47.362
46.191
f1 (ppm)

Compound 7

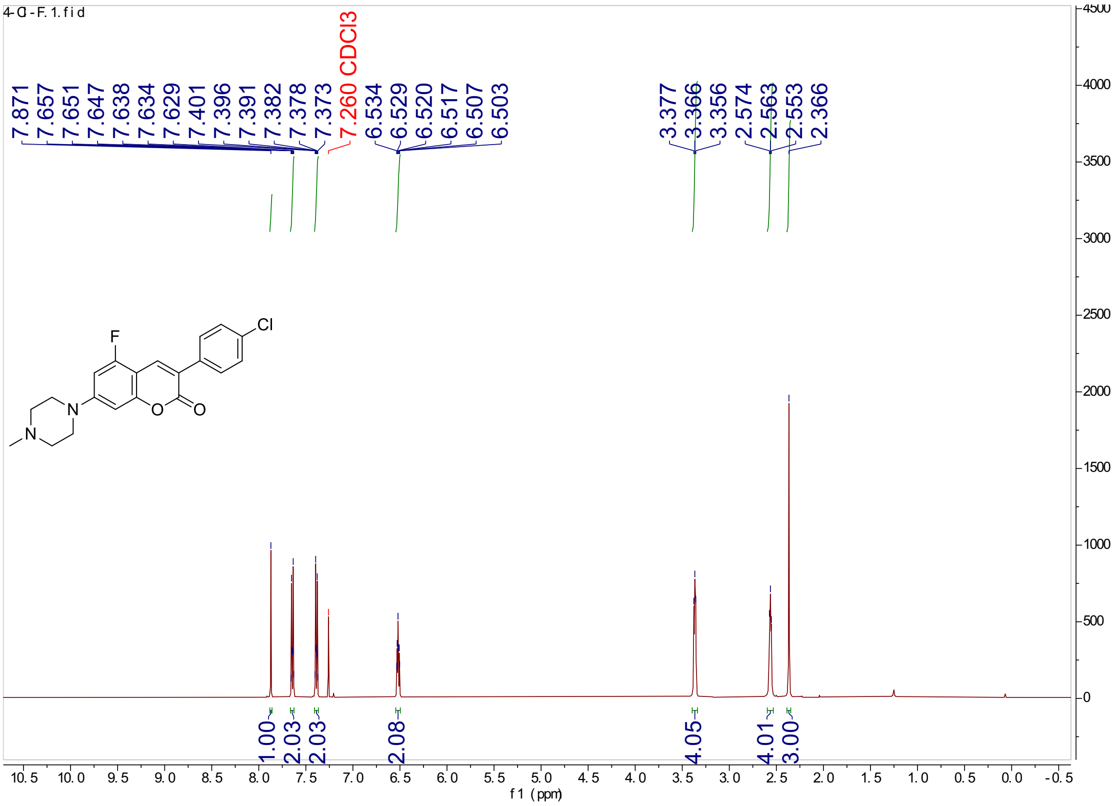
4-Cl-F.1.fid
7.871
7.657
7.651
7.647
7.638
7.634
7.629
7.401
7.396
7.391
7.382
7.378
7.373
7.260 CDCl3
6.534
6.529
6.520
6.517
6.507
6.503
3.377
3.366
3.356
2.574
2.563
2.553
2.366
1.00
2.03
2.03
2.08
4.05
4.01
3.00
F
Cl
N
N
O
O
f1 (ppm)

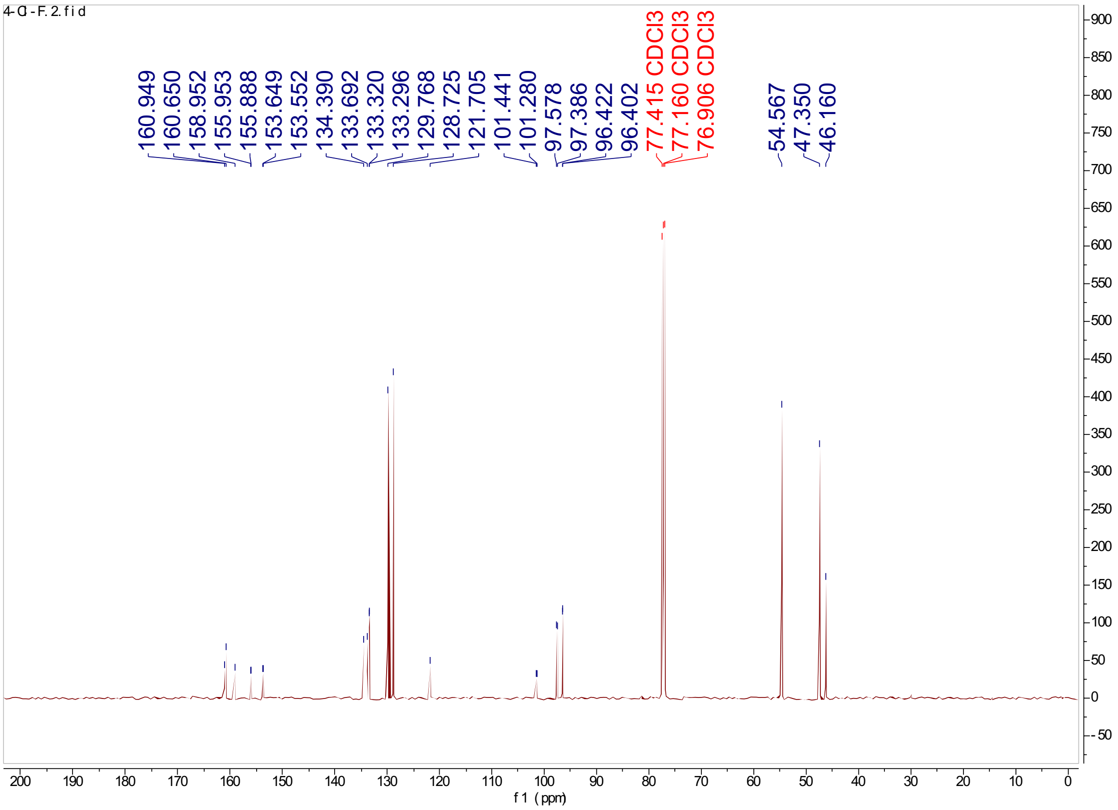
4-Cl-F.2.fid
160.949
160.650
158.952
155.953
155.888
153.649
153.552
134.390
133.692
133.320
133.296
129.768
128.725
121.705
101.441
101.280
97.578
97.386
96.422
96.402
77.415 CDCl3
77.160 CDCl3
76.906 CDCl3
54.567
47.350
46.160
f1 (ppm)

Compound 8

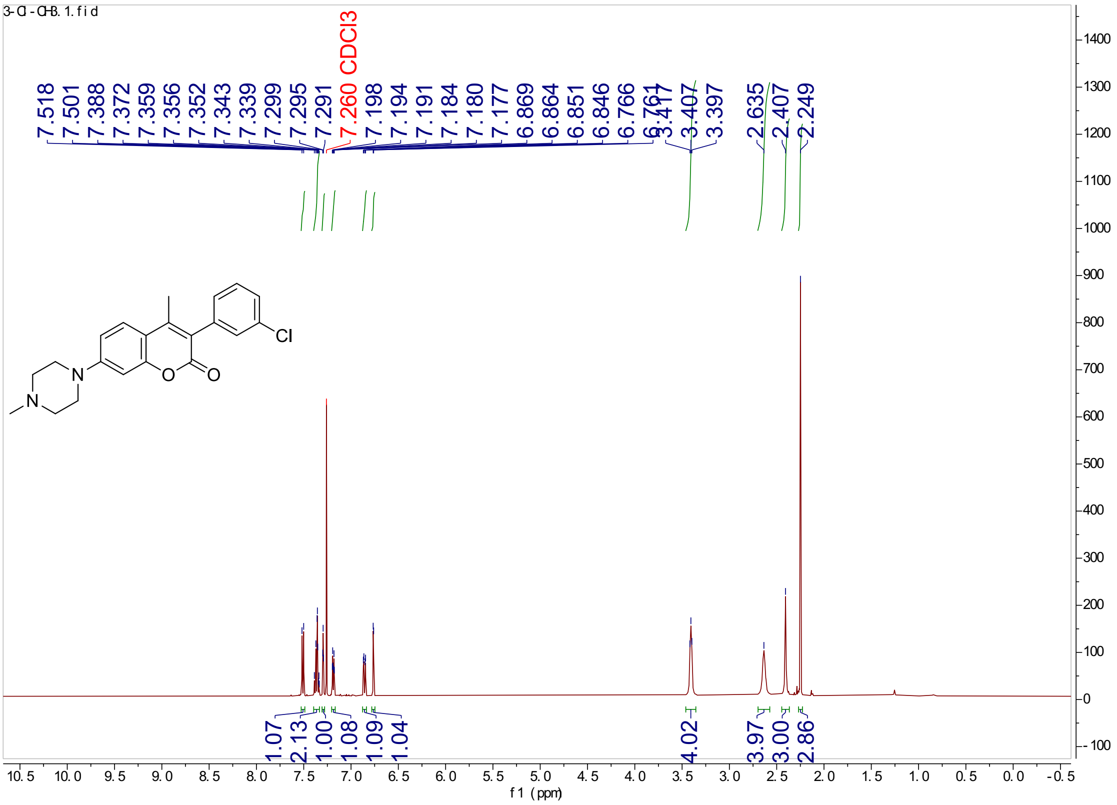
3-Cl-CH3.1.fid
7.518
7.501
7.388
7.372
7.359
7.356
7.352
7.343
7.339
7.299
7.295
7.291
7.260 CDCl3
7.198
7.194
7.191
7.184
7.180
7.177
6.869
6.864
6.851
6.846
6.766
6.761
3.417
3.407
3.397
2.635
2.407
2.249
Cl
N
N
O
O
1.07
2.13
1.00
1.08
1.09
1.04
4.02
3.97
3.00
2.86
f1 (ppm)

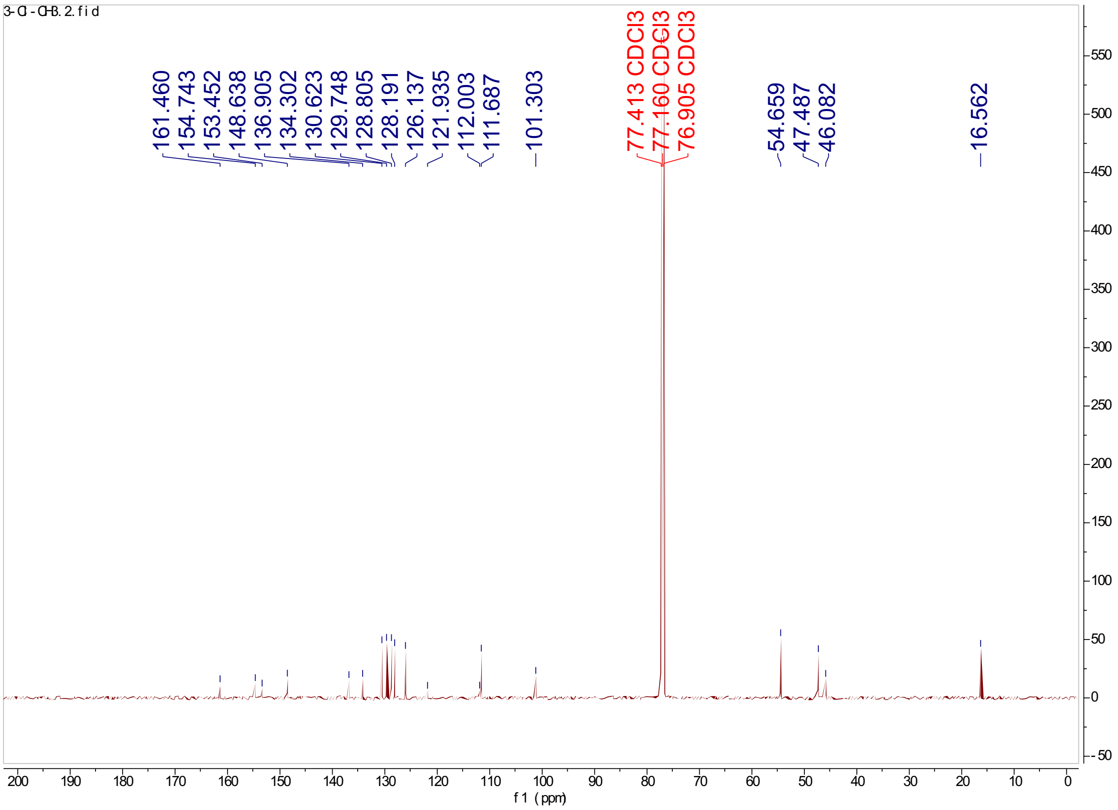
3-Cl-CH3.2.fid
161.460
154.743
153.452
148.638
136.905
134.302
130.623
129.748
128.805
128.191
126.137
121.935
112.003
111.687
101.303
77.413 CDCl3
77.160 CDCl3
76.905 CDCl3
54.659
47.487
46.082
16.562
f1 (ppm)

Compound 9

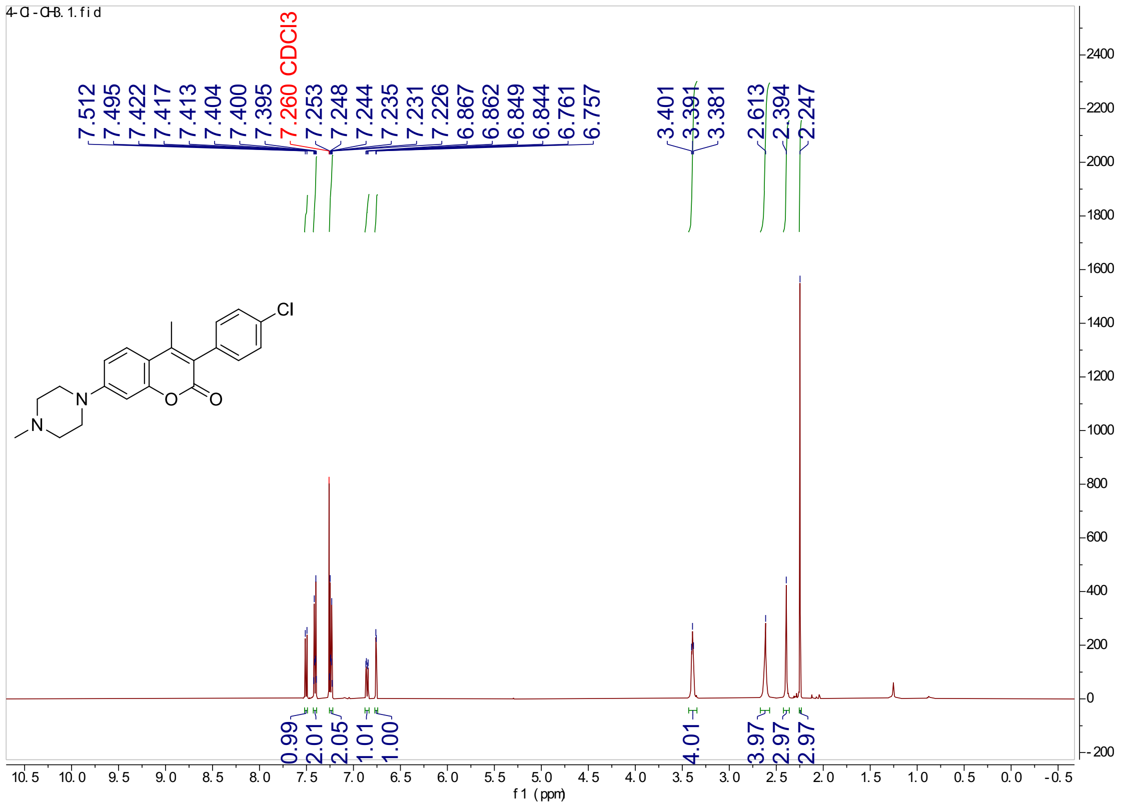
4-Cl-CHB.1.fid
7.512
7.495
7.422
7.417
7.413
7.404
7.400
7.395
7.260 CDCl3
7.253
7.248
7.244
7.235
7.231
7.226
6.867
6.862
6.849
6.844
6.761
6.757
3.401
3.391
3.381
2.613
2.394
2.247
0.99
2.01
2.05
1.01
1.00
4.01
3.97
2.97
2.97
f1 (ppm)

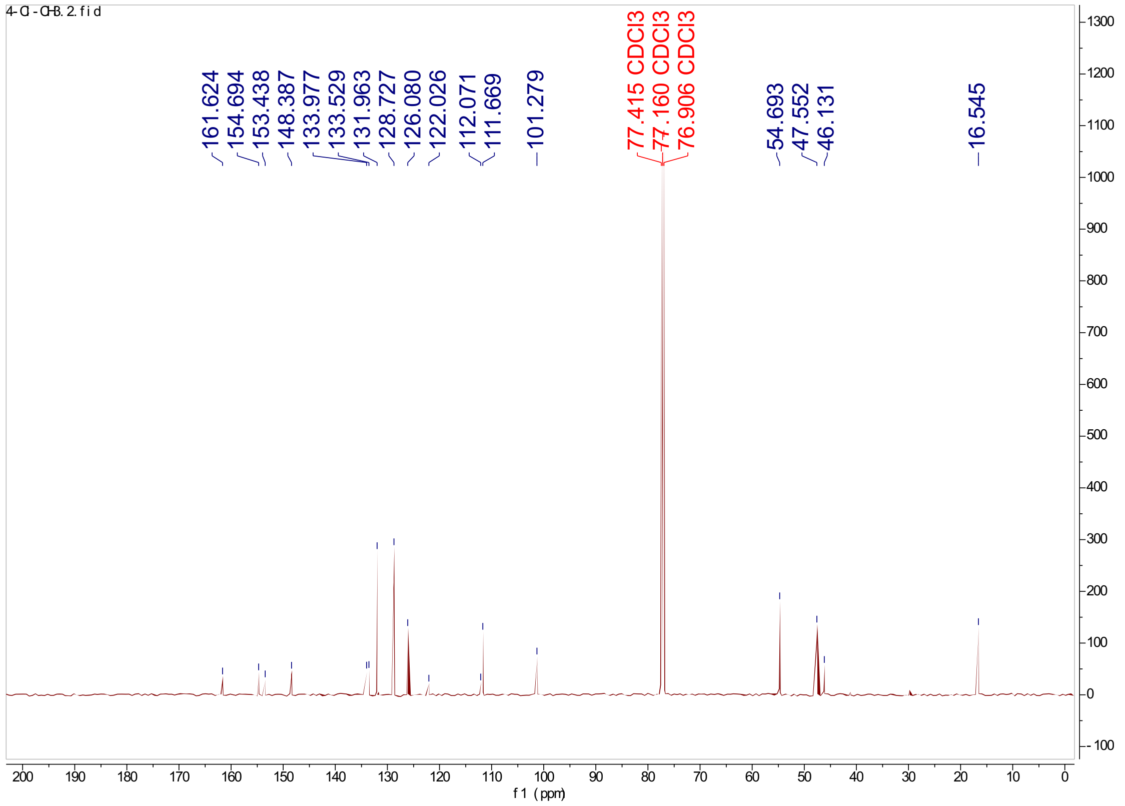
4-Cl-CHB.2.fid
161.624
154.694
153.438
148.387
133.977
133.529
131.963
128.727
126.080
122.026
112.071
111.669
101.279
77.415 CDCl3
77.160 CDCl3
76.906 CDCl3
54.693
47.552
46.131
16.545
f1 (ppm)

Compound **10**

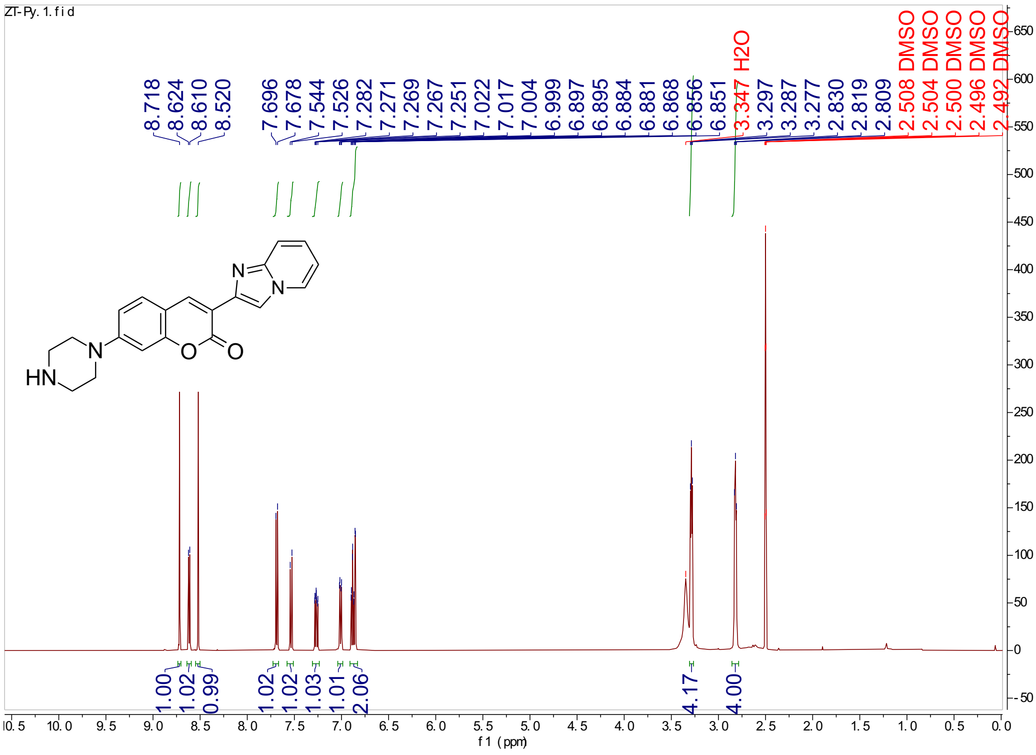

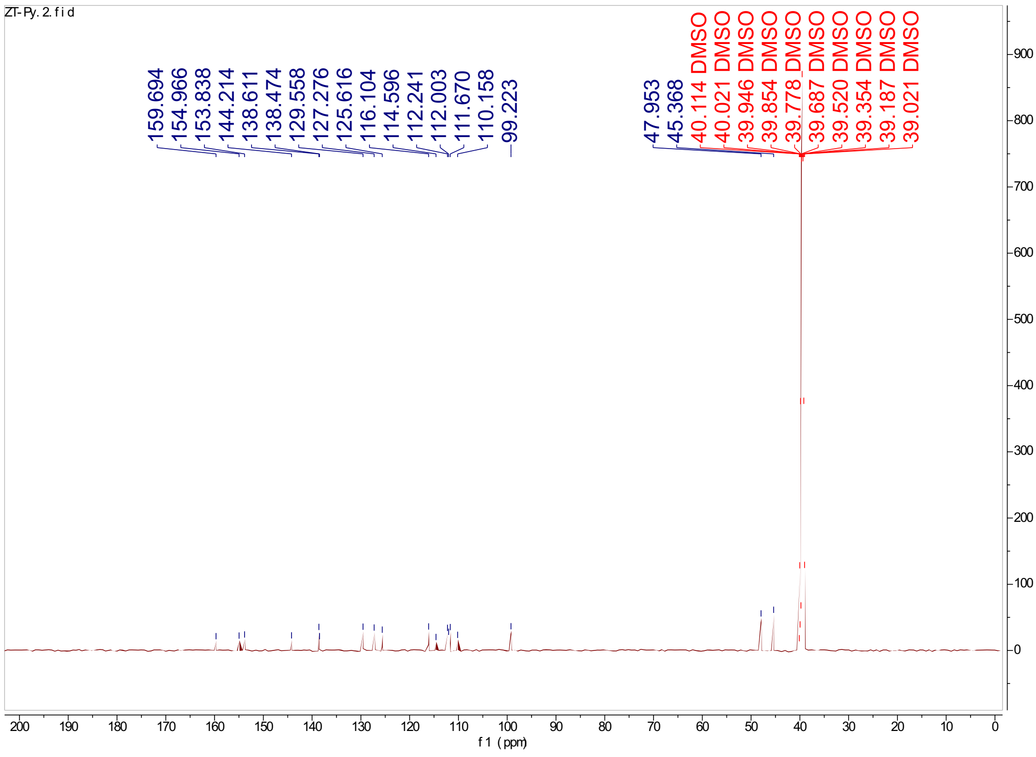

Compound **11**

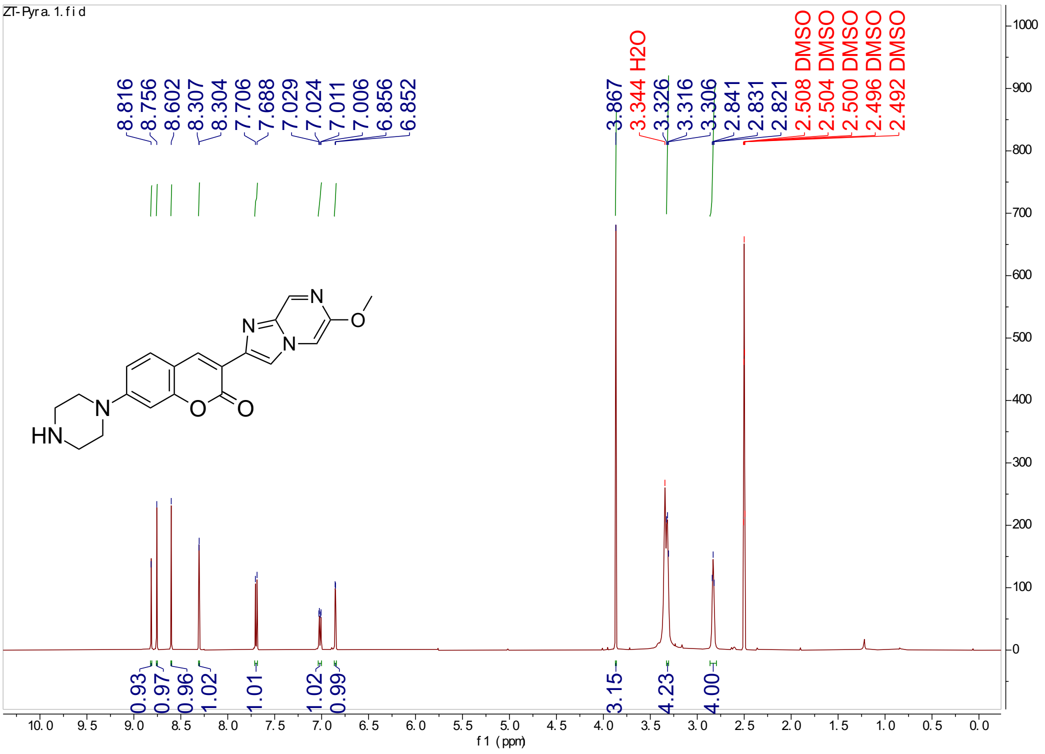

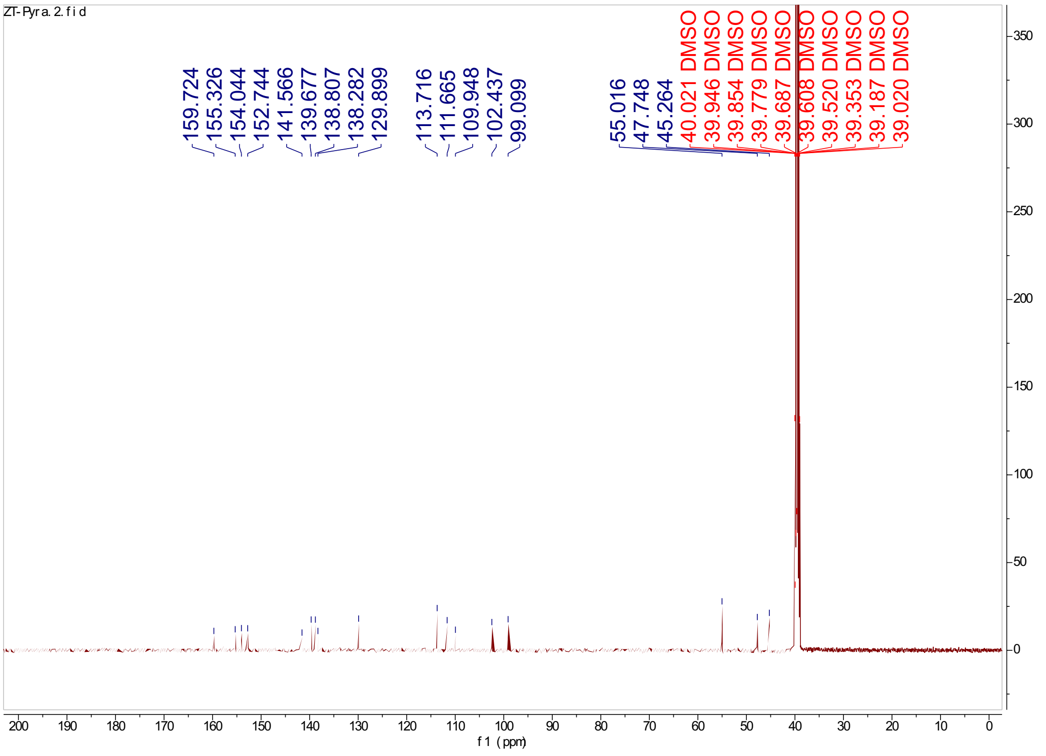

HPLC analysis

SMN-C2

Empower 3

Default Individual Report

SAMPLE INFORMATION

Sample Name:	SMN-C2	Acquired By:	System
Sample Type:	Unknown	Sample Set Name	1220
Vial:	1:B,1	Acq. Method Set:	L4ABT2
Injection #:	1	Processing Method	11
Injection Volume:	3.00 ul	Channel Name:	PDA Ch1 254nm@4.8nm
Run Time:	23.0 Minutes	Proc. Chnl. Descr.:	PDA Ch1 254nm@4.8nm
Date Acquired:	12/20/2020 1:31:31 PM CST		
Date Processed:	12/20/2020 6:14:43 PM CST		

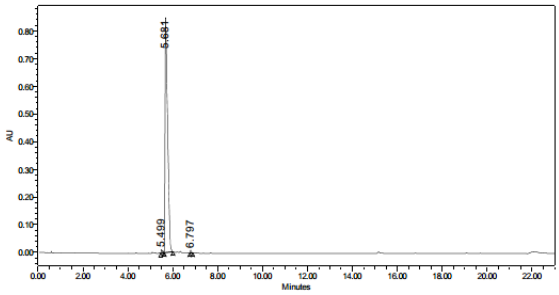

	RT	Area	% Area	Height
1	5.499	47226	0.71	13047
2	5.681	6612904	98.79	848656
3	6.797	34027	0.51	10574

Reported by User: System
Report Method: Default Individual Report
Report Method ID: 1003
Page: 1 of 1

Project Name: UPLC
Date Printed:
12/20/2020
6:14:58 PM US/Central

SMN-C3

Empower 3

Default Individual Report

SAMPLE INFORMATION

Sample Name:	SMN-C3	Acquired By:	System
Sample Type:	Unknown	Sample Set Name	1220
Vial:	1:B,2	Acq. Method Set:	L4ABT2
Injection #:	1	Processing Method	1
Injection Volume:	3.00 ul	Channel Name:	PDA Ch1 254nm@4.8nm
Run Time:	23.0 Minutes	Proc. Chnl. Descr.:	PDA Ch1 254nm@4.8nm
Date Acquired:	12/20/2020 1:55:10 PM CST		
Date Processed:	12/20/2020 6:11:34 PM CST		

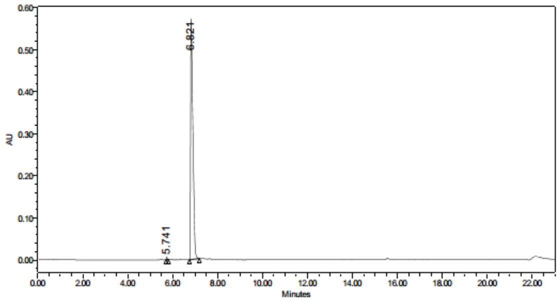

	RT	Area	% Area	Height
1	5.741	16472	0.41	7071
2	6.821	4033175	99.59	571605

Reported by User: System
Report Method: Default Individual Report
Report Method ID: 1003
Page: 1 of 1

Project Name: UPLC
Date Printed:
12/20/2020
6:12:04 PM US/Central

SMN-C5

Empower 3 — Default Individual Report

SAMPLE INFORMATION

Sample Name:	SMN-C5	Acquired By:	System
Sample Type:	Unknown	Sample Set Name	1220
Vial:	1:B,3	Acq. Method Set:	L4ABT2
Injection #:	1	Processing Method	11
Injection Volume:	3.00 ul	Channel Name:	PDA Ch1 254nm@4.8nm
Run Time:	23.0 Minutes	Proc. Chnl. Descr.:	PDA Ch1 254nm@4.8nm
Date Acquired:	12/20/2020 2:18:48 PM CST		
Date Processed:	12/20/2020 6:13:05 PM CST		

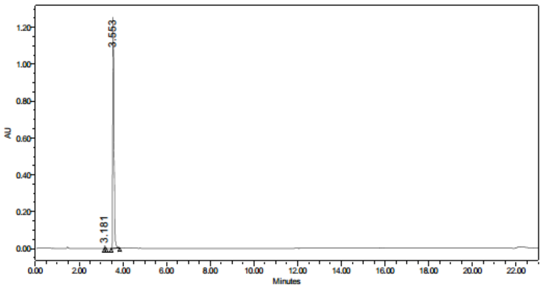

	RT	Area	% Area	Height
1	3.181	41175	0.80	15399
2	3.553	5118735	99.20	1253750

Reported by User: System
Report Method: Default Individual Report
Report Method IC1003
Page: 1 of 1

Project Name: UPLC
Date Printed:
12/20/2020
6:13:26 PM US/Central

Compound 1

Empower 3 — Default Individual Report

SAMPLE INFORMATION

Sample Name:	A1	Acquired By:	System
Sample Type:	Unknown	Sample Set Name	1220
Vial:	1:B,4	Acq. Method Set:	L4ABT2
Injection #:	1	Processing Method	A1
Injection Volume:	5.00 ul	Channel Name:	PDA Ch1 254nm@4.8nm
Run Time:	23.0 Minutes	Proc. Chnl. Descr.:	PDA Ch1 254nm@4.8nm
Date Acquired:	12/20/2020 2:42:32 PM CST		
Date Processed:	12/20/2020 6:09:46 PM CST		

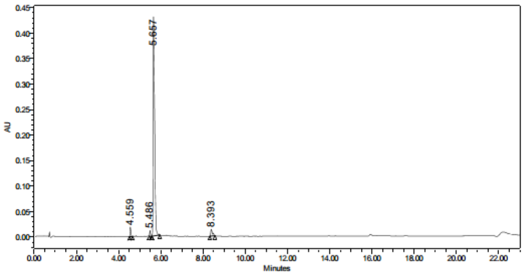

	RT	Area	% Area	Height
1	4.559	56769	2.42	19029
2	5.486	29994	1.28	11523
3	5.657	2187525	93.11	429587
4	8.393	75047	3.19	13379

Reported by User: System
Report Method: Default Individual Report
Report Method IC1003
Page: 1 of 1

Project Name: UPLC
Date Printed:
12/20/2020
6:10:04 PM US/Central

REFERENCES

1. Miao,Y., Feher,V.A. and McCammon,J.A. (2015) Gaussian Accelerated Molecular Dynamics: Unconstrained Enhanced Sampling and Free Energy Calculation. *J. Chem. Theory Comput.*, **11**, 3584–3595.
2. Miao,Y., Sinko,W., Pierce,L., Bucher,D., Walker,R.C. and McCammon,J.A. (2014) Improved Reweighting of Accelerated Molecular Dynamics Simulations for Free Energy Calculation. *J. Chem. Theory Comput.*, **10**, 2677–2689.
3. Woll,M.G., Qi,H., Turpoff,A., Zhang,N., Zhang,X., Chen,G., Li,C., Huang,S., Yang,T., Moon,Y.C., *et al.* (2016) Discovery and Optimization of Small Molecule Splicing Modifiers of Survival Motor Neuron 2 as a Treatment for Spinal Muscular Atrophy. *J. Med. Chem.*, **59**, 6070–6085.
